# Adjunctive ibuprofen in pre-extensively drug-resistant and extensively drug-resistant tuberculosis: a phase IIA open-label pilot clinical trial

**DOI:** 10.1038/s41467-026-75148-9

**Published:** 2026-07-01

**Authors:** Kaori L. Fonseca, Judith Farrés, Ketevan Barbakadze, Iza Jikia, Malkhaz Tsotskhalashvili, Juan M. García-Illarramendi, Lilibeth Arias, Kennedy Otwombe, Chiara Sopegno, Albert Despuig, Neil Martinson, Nadiia Buhiichyk, Tamta Korinteli, Émilie Layre, Jérôme Nigou, Zaza Avaliani, Nestani Tukvadze, Sergo Vashakidze, Cristina Vilaplana

**Affiliations:** 1 Germans Trias i Pujol Research Institute (IGTP), Badalona, Catalonia Spain; 2 Centro de Investigación Biomédica en Red de Enfermedades Respiratorias (CIBERES), Madrid, Spain; 3 National Center for Tuberculosis and Lung Diseases, Tbilisi, Georgia; 4 Institute of Biotechnology and Biomedicine, Universitat Autònoma de Barcelona, Barcelona, Spain; 5 Vall d’Hebron Institut de Recerca (VHIR), Barcelona, Spain; 6 Perinatal HIV Research Unit (PHRU), Faculty of Health Sciences, University of the Witwatersrand, Johannesburg, South Africa; 7 School of Public Health, Faculty of Health Sciences, University of the Witwatersrand, Johannesburg, South Africa; 8 Universitat Pompeu Fabra, Barcelona, Spain; 9 Germans Trias i Pujol Hospital, Fundació Lluita contra la Sida i les Malalties Infeccioses, Barcelona, Spain; 10 Institut de Pharmacologie et de Biologie Structurale (IPBS), Université de Toulouse, CNRS, UPS, Toulouse, France; 11 Swiss Tropical and Public Health Institute, Basel, Switzerland; 12 The University of Georgia, Tbilisi, Georgia; 13 Microbiology Department, Northern Metropolitan Clinical Laboratory, Germans Trias i Pujol University Hospital, Badalona, Catalonia Spain

**Keywords:** Tuberculosis, Biomarkers, Clinical trials

## Abstract

Drug-resistant tuberculosis remains a major global health challenge, and excessive inflammation may contribute to tissue damage and poor outcomes. We conducted a phase IIA prospective open-label pilot trial (ClinicalTrials.gov: NCT02781909) evaluating adjunctive ibuprofen in adults with pulmonary pre-extensively drug-resistant (pre-XDR) or extensively drug-resistant (XDR) tuberculosis in Georgia. Twenty-eight participants received individualized background anti-tuberculosis regimens alone (*n* = 14) or with ibuprofen 400 mg daily for 2 months (*n* = 14) and were followed for 6 months. The primary efficacy outcome was the proportion of participants showing clinical and/or microbiological benefit, assessed by sputum culture conversion, radiological evolution, and WHO-defined treatment outcomes. Secondary outcomes were safety and tolerability, health-related quality of life, and inflammatory and transcriptomic responses. Adding adjunctive ibuprofen did not improve month-2 sputum culture negativity, time to stable culture conversion, radiological evolution, or final treatment outcomes. Safety and tolerability were similar between groups. Adverse events were recorded and reviewed, but not formally graded for severity. Ibuprofen treatment was associated with numerically greater reductions in several blood-based inflammatory measures and in transcriptomic signature scores associated with poor tuberculosis outcomes. These findings are exploratory and do not demonstrate clinical benefit but indicate biological activity and support evaluation of anti-inflammatory host-directed therapies in larger controlled trials.

## Abstract (web summary)

This pilot study reports the use of ibuprofen, an anti-inflammatory drug, as an adjunctive tuberculosis (TB) treatment in adults with extensive drug-resistant pulmonary TB, which shows a reduction of several blood-based inflammatory measures.

## Introduction

Tuberculosis (TB) disease is still claiming millions of lives annually, while imposing economic and social burdens, being one of the major problems for health systems worldwide. In 2024, nearly 400,000 TB cases emerged as multi-drug resistant (MDR) or extensively drug-resistant (XDR), with concerningly low success rates in treatment^1^. Drug-resistant (DR)-TB is a major global public health challenge. Countries of Eastern Europe and Central Asia are home to 24% of the global cases of MDR-TB and 47% of the pre-extensively DR-TB cases (pre-XDR-TB)^2^. This poses a problem for European health systems, which suffer from the increased complications associated with these forms of the disease. In Georgia, the National Center for Tuberculosis and Lung Diseases (NCTLD) serves as a national referral centre for the management of complex drug-resistant TB, where patients with pre-XDR-TB and XDR-TB represent a substantial proportion of admissions. Treatment for MDR/XDR-TB is associated with poor outcomes and high mortality rates^3^. Given their greater disease severity, limited treatment options, and worse prognosis compared with rifampicin-resistant TB overall, this study focused on patients with pre-XDR-TB and XDR-TB. Despite recent advances and new treatment guidelines introducing new regimens, novel approaches to reduce morbidity and mortality, shorten treatment duration, and prevent the emergence of new drug resistance are still urgently needed.

Host immune responses and cellular processes are key to disease progression, making them valuable targets for developing new therapies that hinder pathogen survival and pathogenesis. Host-directed therapies (HDT) have emerged as a promising alternative to enhance treatment outcomes by modulating the immune system, tackling tissue damage and hyperinflammation, reducing treatment duration, and minimising drug-resistance risk^4,5^. As hyperinflammation causes lung damage associated with worse outcomes and the presence of sequelae, the use of repurposed anti-inflammatory drugs as adjuncts to standard treatment is a promising option^6^.

Several drugs have been proposed as HDTs, based on in vitro studies and/or theoretical rationale. However, few have been tested in clinical trials and some -such as thalidomide, etanercept, and others-are associated with severe adverse effects^7^. Despite the urgency of the TB epidemic and its high mortality rate, low-risk HDT options remain largely unexplored in clinical trials.

Ibuprofen is a non-steroidal anti-inflammatory drug (NSAID) and a non-selective cyclooxygenase inhibitor (prostaglandin [PG]H1, PGH2, commonly known as cyclooxygenase [COX]−1 and COX-2) that limits the biosynthesis of prostaglandins. It has been widely used for decades to relieve pain and fever, as well as to treat inflammatory diseases. Ibuprofen has a well-established safety profile and is highly affordable^8^.

Pre-clinical studies with over-the-counter NSAIDs have shown beneficial effects in a murine model of active TB. Ibuprofen has previously been shown to reduce lung pathology and mycobacterial burden in a highly susceptible mouse model of TB, increasing its survival. In the absence of TB treatment, ibuprofen markedly and significantly increased survival and decreased both the bacillary load and granuloma in mice treated with ibuprofen^9^. This effect is likely due to NSAIDs reducing pro-inflammatory mediators (tumour necrosis factor [TNF], interleukin [IL]−17, IL-6 and C-X-C motif chemokine ligand 5 [CXCL5]) release rather than a direct toxic effect on mycobacteria. Previous clinical studies on NSAIDs as HDT have also shown promising benefits, mainly in individuals suffering from tuberculous meningitis, though ibuprofen has never previously been tested^10–12^. We hypothesized that adjunctive ibuprofen could improve outcomes in patients with pre-XDR/XDR-TB. We focused on pre-XDR/XDR-TB as a high-morbidity population managed at the national referral centre and to evaluate feasibility, safety and biological activity of adjunctive ibuprofen in the context of complex second-line regimens. To explore this, we conducted a pilot study evaluating the effect of adding 400 mg of ibuprofen daily to background anti-TB regimens on early sputum culture conversion, radiological changes, and inflammatory markers, as well as safety and tolerability over two months. As a pilot study, this trial aimed to assess the magnitude and direction of possible responses rather than to detect small differences. It was not powered for formal hypothesis testing; effect estimates are presented descriptively and intended to guide the design of a subsequent larger trial.

## Results

### Participant characteristics and clinical data on TB episode

**Fig. 1:**
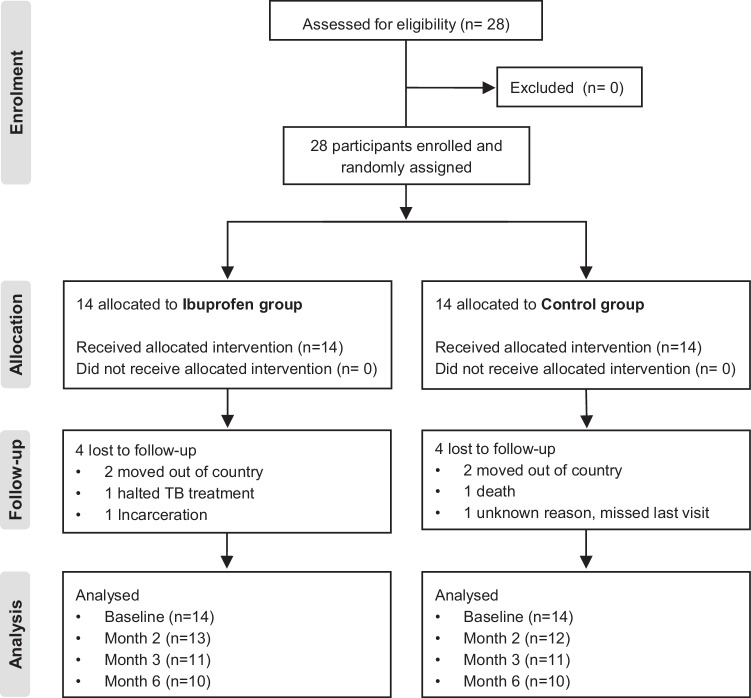
Study flow diagram. CONSORT flow diagram of the NSAIDS-XDR-TB pilot trial. Participants were assessed for eligibility and allocated in a 1:1 ratio to either the control group, receiving the background anti-TB regime, or the Ibuprofen-treated group, receiving the background anti-TB regime plus ibuprofen 400 mg daily for 2 months, as an adjunctive therapy. Analyses included all available participant data at each time point.

A total of 28 participants diagnosed with a recurrent episode of TB were screened and assessed for eligibility between October 2016 and February 2018. All participants were enroled and allocated by alternation to either receive the background anti-TB treatment alone (control group, *n* = 14) or plus Ibuprofen (ibuprofen-treated group, *n* = 14). Background regimens were individualised and heterogeneous (Supplementary Table 2). Linezolid-containing regimens were slightly more frequent in the ibuprofen arm (11/14 vs 9/14), whereas bedaquiline use was similar between arms (7/14 in each). The NSAIDS-XDR-TB flow diagram is showed in Fig. 1.

The demographic and clinical baseline characteristics of the participants enroled are presented in Table 1 and Supplementary Table 9. Participants’ median age was 44 years, and 75% of the participants were male. The ibuprofen-treated group had a higher proportion of female participants.

**Table 1:** Baseline characteristics by experimental group

	Overall (*N* = 28)	Control (*N* = 14)	Ibuprofen (*N* = 14)
Demography			
Sex, *N* (%)^b^			
Male	21 (75%)	13 (92%)	8 (57%)
Female	7 (25%)	1 (7%)	6 (43%)
Age (years), Median (IQR)	44 (34 –55)	53 (32 –57)	40 (32 –46)
Risk factors			
Smoking, *N* (%)			
Never	7 (25%)	2 (14%)	5 (36%)
Less than monthly	5 (18%)	3 (21%)	2 (14%)
Daily or almost daily	16 (57%)	9 (64%)	7 (50%)
Alcohol, *N* (%)			
Never	12 (43%)	6 (43%)	6 (43%)
Less than monthly	14 (50%)	6 (43%)	8 (57%)
Monthly	1 (4%)	1 (7%)	0 (0%)
Weekly	1 (4%)	1 (7%)	0 (0%)
Co-morbidities			
Diabetes, *N* (%)	4 (14%)	2 (14.3%)	2 (14.3%)
COPD previous to the TB, *N* (%)	1 (4%)	1 (7)	0 (0%)
Hepatitis C Virus, *N* (%)	8 (29%)	4 (28%)	4 (29%)
Concomitant medication			
Antidiabetics, *N* (%)	4 (14%)	2 (14%)	2 (14%)
TB medical history			
New patient, *N* (%)	9 (32%)	4 (29%)	5 (36%)
Relapse, *N* (%)	1 (4%)	0 (0%)	1 (7%)
Treatment after loss to follow-up, *N* (%)	15 (54%)	9 (64%)	6 (43%)
Unknown previous TB treatment, *N* (%)	3 (11%)	1 (7%)	2 (14%)
TB drug sensitivity			
Pre-XDR, *N* (%)	8 (29%)	5 (38%)	3 (21%)
XDR, *N* (%)	20 (71%)	9 (64%)	11 (79%)
TB episode–Clinical data			
BMI (kg/m2), Median (IQR)	21 (19–26)	21 (19–24)	21 (18–30)^a^
Weight (kg), Median (IQR)	64 (57–77)	64.5 (55.75–78)	64 (55.75–75.5)
Presence of fever, *N* (%)^b^	24 (83%)	10 (67%)	14 (100%)
Presence of shortness of breath, *N* (%)^b^	17 (59%)	6 (40%)	11 (79%)
Presence of cavities, *N* (%)	25 (89%)	12 (86%)	13 (93%)
TB severity score, Median (IQR)^b^	6.0 (4.0–8.0)	5.5 (3.0–6.0)	7 (5.5–8.0)^a^
X-ray score, Median (IQR)	5.5 (5.0–7.0)	5.5 (4.0–7.0)	5.5 (5.0–7.0)
SGRQ score, Median (IQR)^b^	87 (46–93)	59 (37–87)	90 (84–95)
K10 score, Median (IQR)	30 (26–35)	30 (17–35)	30 (30–35)

*COPD* chronic obstructive pulmonary disease, *BMI* body mass index, *IQR* interquartile range, *XDR* extensively drug-resistant, *TB* tuberculosis, *SGRQ* Saint George Respiratory Questionnaire, *K10* Kessler Psychological Distress Scale.

^a^*n* = 13 for the Ibu-treated group.

^b^Indicates notable between-arm difference at baseline (descriptive only; no formal statistical testing of baseline characteristics).

Risk factors were related to smoking and alcohol consumption, with the proportions equivalent between experimental groups. The most frequently reported comorbidities, present in at least 14% of participants, were diabetes and hepatitis C virus co-infection. Considering the overall TB medical history of the participants, the 54% (15 out of 28) were treated for recurrent TB following a previous episode complicated by loss to follow-up, and TB drug resistance profiles were comparable between the treatment groups. In the control group, five participants (38%, 5 out of 14) had pre-XDR-TB, and nine participants (64%, 9 out of 14) had XDR-TB. In the ibuprofen-treated group, three participants (21%, 3 out of 14) had pre-XDR-TB, and eleven participants (79%, 11 out of 14) had XDR-TB.

The ibuprofen-treated group exhibited evidence of worse disease severity at baseline compared to the control group (Table 1, Supplementary Table 9). Specifically, the control group had a baseline median TB severity score of 5.5 (interquartile range [IQR] 3–6.25), whereas participants in the ibuprofen-treated group had a score of 7 (IQR 5.5–8). The TB severity score is a clinical tool that quantifies TB disease severity based on various signs and symptoms, including fever and shortness of breath. Notably, these two symptoms also showed an imbalance between groups, with fever present in 67% (10 out of 14) of the control group vs. 100% of the ibuprofen-treated group and shortness of breath in 40% (6 out of 14) vs. 79% (11 out of 14), respectively. Baseline albumin levels were also significantly lower in the ibuprofen-treated group compared with the control group (mean 29.7 g/L [SD 5.3] vs. 34.6 g/L [SD 4.8]). The baseline severity differences were consistent with the Saint George Respiratory Questionnaire (SGRQ) score, which was 59 (IQR 36–88.25) in the control group, compared to 90 (IQR 80–95.25) in the ibuprofen-treated group.

### Microbiological changes during follow-up

**Fig. 2:**
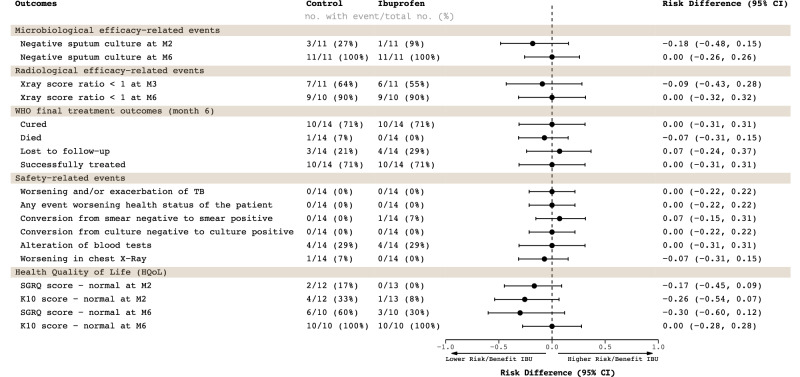
Primary and secondary outcomes: risk difference. Risk difference is represented by the dot, and the 95% confidence intervals (CIs) for risk difference are represented by the error bars and were calculated using the Newcombe method for differences in proportions. Sample sizes vary by outcome and time point: total *n* = 28 participants (Control: *n* = 14; Ibuprofen: *n* = 14), with available data varying at M2, M3, and M6 follow-up. Each participant represents an independent biological unit of study. X-ray, a score that accounted for key radiological signs, including primary and secondary parenchymal abnormalities, nodular lesions, and pleural abnormalities; SGRQ, Saint George Respiratory questionnaire; K10, Kessler Psychological Distress Scale; M2, month 2; M6, month 6.

**Fig. 3:**
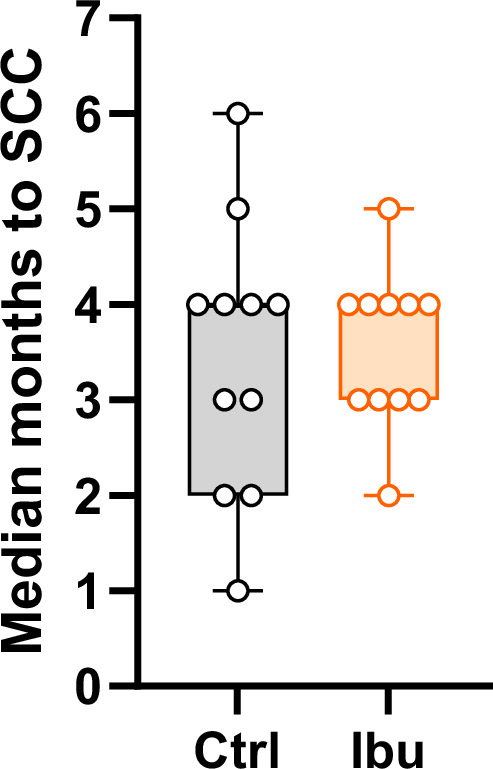
Primary outcome, time to stable sputum conversion. The median time to sputum culture conversion (SCC) in months for each treatment group is shown. Box plots depict the median (centre line), interquartile range (IQR; box boundaries represent the 25th and 75th percentiles), and minimum/maximum values (whiskers extending to the smallest and largest observed values). Each dot represents the month of SCC for each patient. Statistical analysis was performed using an unpaired two-tailed Mann–Whitney test, *p* value = 0.92. Ctrl: Control group *n* = 11, Ibu: Ibuprofen group *n* = 11.

Microbiological efficacy was assessed as the proportion of participants with a stable negative sputum culture at month 2 among those with available culture data (Supplementary Table 10). No significant differences were observed: 27% (3/11) of the control group versus 9% (1/11) of the ibuprofen group achieved culture negativity at month 2. The wide confidence interval for the risk difference (RD = 18%, 95% CI −13 to 50) indicates substantial imprecision, and the small sample size limits meaningful between-group comparisons. By month 6, all participants in both groups had negative sputum cultures (Fig. 2). The median time to sputum culture conversion was four months in both groups, IQR (2.5–4) for the control group and IQR (3–4) for the ibuprofen group (Fig. 3 and Supplementary Table 11).

### Radiological changes during follow-up

Radiological efficacy was assessed using an X-ray score that accounted for key radiological signs, including primary and secondary parenchymal abnormalities, nodular lesions, and pleural abnormalities. No differences were observed between groups in the proportion of participants showing improvement over time. At month 3, 7/11 (64%) participants in the control group and 6/11 (55%) in the ibuprofen group showed radiographic improvement, corresponding to an RD of 9% (95% CI −32 to 50; control minus ibuprofen). By month 6, all participants had improved compared to baseline, and there was no between-group difference (RD 0%, 95% CI − 26 to 26) (Fig. 2 and Supplementary Fig. 1a, Supplementary Table 11). The wide confidence intervals for the risk difference indicate substantial imprecision, and the small sample size limits meaningful between-group comparisons.

### Final treatment outcomes according to WHO definitions

When evaluating the proportion of participants on final treatment outcomes based on the WHO definition, no differences were observed between experimental groups, with 71% achieving successful treatment. In the control group, 71% of participants were classified as cured (10 out of 14), 21% (3 out of 14) were lost to follow-up, and 8% (1 out of 13) died. In the ibuprofen-treated group, 71% (10 out of 14) of participants were cured, 29% (4 out of 14) were lost to follow-up, and no participant died (Fig. 2 and Supplementary Table 10).

### Safety-related events

The incidence of safety-related events was similar between the control and ibuprofen groups (Fig. 2), the most frequent being alterations in blood tests. All events were transient and none required intervention. A complete list of safety-related events (recorded throughout the study period) is provided in Table 2.

**Table 2:** Safety-related events

Arm	Patient ID	Timepoint	Adverse Event	Short justification with likeliest explanation	Intervention required and time of Recovery
Control	NSAIDS-XDR-TB-CT-2	Month 1	Laboratory test alteration (Platelets ↓; AST ↑)	Platelet fall, uncertain reason; not attributable to Lzd.	No intervention required. recovered by Month 2
Control	NSAIDS-XDR-TB-CT-5	Month 1	Laboratory test alteration (Platelets ↓); Worsening of the CXR	Platelet fall likely multifactorial (resolving inflammation or other regimen effects); patient not on Lzd.	No intervention required. Recovered by Month 2.
Control	NSAIDS-XDR-TB-CT-12	Month 1	Laboratory test alteration (Platelets ↓; Creatinine ↑)	Platelet change likely multifactorial; AKI likely multifactorial.	No intervention required. Recovered by Month 2
Control	NSAIDS-XDR-TB-CT-13	Month 1	Laboratory test alteration (Platelets ↓; modest AST ↑)	Large platelet decline plausibly related to Lzd exposure; AST change modest and nonspecific.	No intervention required. Recovered by Month 2
Ibuprofen	NSAIDS-XDR-TB-IBU-1	Month 1	Laboratory test alteration (Platelets ↓, AST ↑)	Likely Lzd-related (platelets). Regimen includes Lzd; AST rise nonspecific, likely TB treatment.	No intervention required.Lab results: Partial improvement on follow-up visit (Month 2); recovered by Month 3.
Ibuprofen	NSAIDS-XDR-TB-IBU-3	Month 1	Laboratory test alteration (Haemoglobin ↓)	Anaemia likely multifactorial; no bleeding recorded.	No intervention required. Recovered by Month 2
Ibuprofen	NSAIDS-XDR-TB-IBU-5	Month 1	Laboratory test alteration (Clinician-flagged minor fluctuations)	Clinician-flagged minor laboratory changes	No intervention required
Ibuprofen	NSAIDS-XDR-TB-IBU-12	Month 1	Laboratory test alteration (Creatinine ↑; AST ↑)	Acute Kidney Injury (AKI) and transient transaminitis observed; AKI appears multifactorial (illness/volume/drug interactions)	No intervention required. Recovered by Month 2

Safety-related events were recorded throughout the study period. Safety-related events comprised: (1) clinical worsening of TB, (2) failure to achieve expected sputum conversion, (3) worsening on chest X-ray, (4) clinically significant vital-sign abnormalities, (5) clinically relevant laboratory test alterations, and (6) any other event worsening the participant’s health status. For each affected participant, we report the study arm, timepoint of the event, a brief justification, the likeliest explanation, and any intervention undertaken with the timing of recovery. Events were recorded at routine study visits and reviewed by the trial clinical team, but were not formally graded for severity. AKI, acute kidney injury; AST, aspartate aminotransferase; CXR, chest X-ray; Lab, laboratory; Lzd, linezolid; ↓ decrease from the previous timepoint; ↑ increase from the previous timepoint. Laboratory test alteration refers to clinically relevant changes in laboratory parameters detected on routine monitoring, as specified for each participant.

### Changes in health-related quality of life

Health-related quality of life (HQoL) was assessed by the SGRQ^13^ and Kessler-10 (K10)^14^ scores. Participants who showed a score within the normal range (Supplementary Tables 6 and 7) at months 2 and 6 after treatment initiation were considered responders. No differences between study groups were observed at either time point for both scores (Fig. 2). The proportion of participants showing a normal SGRQ score at month 2 were 17% (2 out of 12) in the control group, while none of the participants in the ibuprofen-treated group reached the normal score (0 out of 13) (Fig. 2). At month 6, 60% (6 out of 10) of the control group and 30% (3 out of 10) of the ibuprofen-treated group reached a normal score.

The proportion of participants not experiencing psychological distress at month 2 was 33% (4 out of 12) in the control group and 8% (1 out 13) in the ibuprofen-treated group. At month 6, all participants reached a normal score (Fig. 2). Median values are reported in Supplementary Table 11 and ratios relative to baseline are presented in Supplementary Fig. 1b, c.

### Impact on the immune and lipid mediated responses

The impact on the immune response was assessed by comparing the proportion of study participants showing changes in immune responses at months 2 and 6 relative to baseline. Three types of measures were used to evaluate the immune response: blood cell-derived inflammation index parameters (the neutrophil-to-lymphocyte ratio (NLR), the monocyte-to-lymphocyte ratio (MLR), and the systemic immune-inflammation index (SII)), plasma biomarkers related to immunological processes, and whole blood RNA transcripts.

**Fig. 4:**
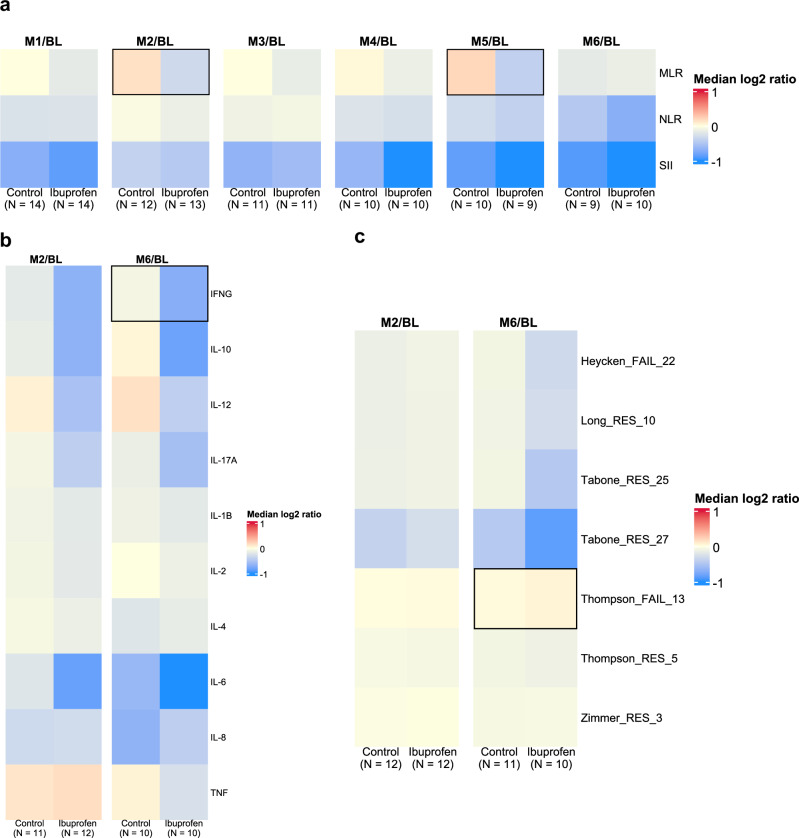
Immune response changes over time. Heat maps depict the median change with respect to baseline. Encapsulated cells denote *p* value < 0·05 in an unpaired two-tailed Mann–Whitney test. Reported p-values are unadjusted and exploratory and are provided for descriptive purposes only. M2, month 2; M6, month 6; Bl, baseline. **a** Blood cell-derived inflammation index parameter changes from baseline. MLR: monocyte-to-lymphocyte ratio; NLR: neutrophil-to-lymphocyte ratio; SII: systemic immune-inflammation index. **b** Plasma biomarkers change **c**. Enrichment score RNA-based response signatures change.

Blood cell-derived inflammation indices generally decreased over time in both arms; numerically larger decreases were observed in the ibuprofen arm than in the control arm for some indices at some timepoints (e.g., MLR at month 2; Fig. 4a and Supplementary Fig. 2). Among the ten plasma biomarkers measured, interferon gamma (IFNG), IL-10, IL-12, IL-17, and IL-6 declined over time in both arms, with numerically larger decreases in the ibuprofen arm for several markers at months 2 and 6 (Fig. 4b and Supplementary Fig. 3). For IFNG, the largest between-arm difference was observed at month 6. IL-1B, IL-2 and IL-4 showed little change in either group. At month 6, IL-8 declined more in the control group, whereas TNF declined more in the ibuprofen group. These between-group patterns should be interpreted cautiously, as the small sample size and imbalance in baseline severity and background anti-TB regimens preclude attribution of immunological differences to ibuprofen.

Analysis of RNA transcript levels over time did not reveal differences between the study groups. However, when comparing the gene expression profile to host blood RNA signatures that predict TB treatment outcomes, we observed a trend like the other immune measures, with the ibuprofen-treated group showing a numerically greater reductions in transcriptomic signature scores associated with risk of treatment failure over time, particularly at month 6. We selected seven published TB gene expression signatures (Supplementary Table 8) that identified patients at risk of treatment failure. Using ssGSEA, we calculated a score for each participant that reflects the resemblance of their gene expression profile to each signature, with higher scores indicating stronger similarity. The biological interpretation of these scores depends on the context of each gene set. For all signatures except Thompson_FAIL_13, higher scores are associated with more severe disease. For Thompson_FAIL_13, the relationship is reversed: higher scores reflect reduced disease severity. We compared changes in signature scores over time between the experimental groups. At month 2, neither study group showed a decrease in the score for the analysed signatures, except for Tabone_RES_2, where both experimental groups showed a reduction in the score (Fig. 4c and Supplementary Fig. 4). By month 6, differences between the experimental groups became more pronounced, with the ibuprofen-treated group showing a greater reduction in disease severity, reflected by a higher decrease in scores for all signatures, and an increase for Thompson_FAIL_13 where the less severity corresponds to a higher score. While small sample sizes and variability preclude definitive conclusions, the most pronounced difference between groups at month 6 was observed for Thompson_FAIL_13.

Finally, in both the control and ibuprofen arms, 13‑HODE, 9‑HODE, 12‑HETE, 8-HETE and 5‑HETE levels increased progressively from baseline to month 6, while 15-HETE, 14‑HDoHE and 17‑HETE rose steadily during treatment (Supplementary Fig. 5). These parallel lipid-mediator shifts likely reflect broad remodelling of arachidonic‑acid metabolism with TB therapy rather than a direct COX‑inhibitory effect of ibuprofen.

## Discussion

HDTs have emerged as promising adjuncts to standard TB therapy, with the potential to reduce inflammation, enhance antibiotic efficacy, and improve patient outcomes. This pilot trial represents the first clinical evaluation of adjunctive ibuprofen for pre-XDR and XDR-TB. Beyond exploring preliminary signals of efficacy and safety, its primary aim was to generate scientific and operational evidence to guide a subsequent larger trial -specifically regarding safety, tolerability, biological activity, and dose selection. Given the limited sample size and budget, we focused on patients with the highest inflammatory burden and most complex treatment regimens to maximise the chance of detecting an early signal while confirming safety in the population most in need.

In terms of clinical efficacy, our study found comparable outcomes between patients treated with 400 mg of adjunctive ibuprofen and those receiving standard of care alone. Similar percentages of participants in both treatment arms achieved negative sputum cultures at months 2 and 6. The median time to stable sputum culture conversion was four months in each group, which is slower than typically reported with contemporary standardised DR-TB regimens, particularly those containing bedaquiline^15,16^. However, this finding may be consistent with patients with advanced drug resistance managed with individualised longer regimens and a high baseline disease burden, which may contribute to longer times to culture conversion^15^. Radiological assessments revealed similar results in chest X-ray evolution, despite the ibuprofen-treated group initially presenting with more severe disease. Likewise, WHO-defined treatment outcomes indicated that 71% of participants in both arms were classified as cured or successfully treated, and no differences were found in HQoL outcomes.

The frequency of protocol-defined safety-related events was similar between arms, and no event required intervention. Laboratory test alterations were observed during follow-up in both arms, including during the first 2 months when ibuprofen was administered; however, adverse events were not formally graded for severity and causality was not formally adjudicated, limiting attribution to ibuprofen versus background regimens or intercurrent illness. Ibuprofen is known for its strong anti-inflammatory properties and effective pain relief, with a relatively low risk of adverse effects, particularly in comparison to other NSAIDs^8^. The rationale for administering ibuprofen only during the intensive phase is twofold. First, inflammation peaks early in tuberculosis disease (when bacterial burden is highest and immune‑mediated tissue damage is most severe), so dampening this initial surge may limit pathology and boost treatment response^17^. Second, in clinical practice, anti‑inflammatories are most often used at the onset of treatment, and extending ibuprofen throughout the intensive phase ensured consistency across patients and simplified logistics for both participants and providers.

The low daily dose in this pilot was selected based on preclinical data, published safety profiles, and NSAID dose–tolerability relationships, as well as to minimise potential drug–drug interactions with intensive second-line TB regimens. While this conservative dose may partly explain the absence of measurable improvement in clinical endpoints, it provides a rationale for evaluating higher doses or alternative dosing strategies in subsequent adequately powered trials.

Exploratory immunological readouts showed trajectories consistent with reduced systemic inflammation in the ibuprofen arm, although causal attribution remains uncertain given the pilot design and baseline and regimen imbalances. A higher decrease in MLR was observed in the ibuprofen-treated group at several time points. MLR, NLR, and SII have been explored as markers of progression in TB^18^, and elevated MLR values have been linked to increased TB severity^19^, and have been used in clinical trials to evaluate HDTs^20^. Additionally, a study assessing a PGH2 inhibitor in TB demonstrated reduced pro-inflammatory responses evidenced by diminished phosphorylation potential and signal transduction in monocytes^21^- supporting the potential relevance of our findings. Plasma cytokines similarly showed numerically larger reductions in the ibuprofen arm at some timepoints, although the largest between-arm difference was observed for IFNG at month 6. Interestingly, both pro-inflammatory cytokines (e.g., IFNG) and anti-inflammatory cytokines (e.g., IL-10) followed a similar downward trajectory. These immunological patterns are hypothesis-generating and cannot be attributed specifically to ibuprofen in this small pilot trial. Prostaglandins, particularly PGE2, play a central role in regulating the production of both pro-inflammatory and anti-inflammatory cytokines. One possible explanation is that COX inhibition may modulate prostaglandin-mediated feedback loops and thereby influence cytokine trajectories during treatment. In contrast, cytokines such as IL-1B, IL-2, and IL-4 remained largely unchanged, likely due to their regulation via alternative signalling pathways that are independent of prostaglandin-mediated mechanisms^22^. Finally, we evaluated participants’ transcriptional profiles using seven established blood RNA signatures predictive of TB treatment failure. At month 6, the ibuprofen-treated group demonstrated a greater reduction in risk scores for most signatures compared to the control group, with the Thompson_FAIL_13 signature showing the strongest evidence of between-group differences. The genes involved in this signature are enriched in neutrophil-associated transcripts and expressed higher in treatment failures at baseline, suggesting the presence of a state of enhanced inflammation prior to treatment in patients who will ultimately not be cured^23^.

Despite reductions in immune activation, these changes did not translate into improved microbiological or clinical outcomes between groups. Several factors may contribute to this observation, including the small sample size and baseline differences. Additionally, the degree of inflammation reduction achieved by ibuprofen might have been insufficient to influence the course of TB progression, possibly due to the low dose used or the specific NSAID selected, as other HDTs have been shown to contribute to inflammation resolution and at the same time to significantly improve sputum conversion rates and chest radiological outcomes^20^.

In addition to our study, several trials have evaluated adjunct anti-inflammatory treatments (aspirin, dexamethasone and prednisolone) in TB for tuberculous meningitis (NCT04145258, NCT03927313 and NCT03092817), for tuberculous pericarditis (NCT00810849) and pulmonary TB and type 2 diabetes^12^. Moreover, two registered clinical trials on ClinicalTrials.gov have focused on NSAIDs for pulmonary TB: the TBCOX2 Trial (NCT02503839)^24^, which evaluated etoricoxib (a prostaglandin-endoperoxide synthase 2 (PTGS2) selective inhibitor), and the SMA-TB RCT (NCT04575519)^25^, which evaluated a higher dose of ibuprofen. Although results from the SMA-TB trial are not yet available, etoricoxib appears to be safe; however, the data did not support the use of adjunctive PTGS2 inhibitors, with the authors acknowledging the heterogeneity among TB patients and suggesting that cyclooxygenase inhibitors may benefit specific subgroups^24^. Optimizing dosing or exploring alternative anti-inflammatory agents may be necessary to achieve a more pronounced clinical effect. Moreover, longer follow-up periods may be required to assess whether immune modulation confers sustained benefits in TB treatment and if any of these prevent TB relapse.

We acknowledge several limitations of this pilot trial, most of them due to being conducted as a small-scale, open‑label pilot under severe funding constraints. First, the small sample size and absence of a placebo control preclude formal hypothesis testing; estimates of effect (or lack of effect) are therefore imprecise and should be interpreted descriptively and cautiously. Second, allocation was implemented by simple alternation rather than concealed randomisation. Although the alternating sequence was applied as recorded, this approach is predictable and does not ensure allocation concealment; therefore, selection bias cannot be excluded. This may have contributed to the observed baseline imbalance in disease extent and severity between arms. However, given the very small sample size of this pilot study, such imbalances are also plausibly attributable to chance variation. Third, background anti-TB regimens were heterogeneous and showed some imbalance between arms, which may have influenced sputum culture conversion and other outcomes and increased uncertainty in attributing observed clinical or immunological differences specifically to ibuprofen. Future trials using unified background regimens would better isolate adjunctive drug-specific effects.

Although generating operational lessons was not the pilot’s primary aim, the study yielded practical insights that informed the design of subsequent work. Although no formal numeric progression thresholds were prespecified, advancement to a definitive trial was based on pragmatic go/no-go considerations (acceptable safety and tolerability with intensive second-line regimens, feasibility of recruitment and sample handling at the site, and evidence of a biological signal), all of which were met and directly informed the design and funding of the subsequent placebo-controlled SMA-TB RCT (NCT04575519)^25^. Despite its limitations, the pilot provided useful preliminary insights into safety, tolerability and immunomodulation, and informed dose, outcome and operational choices for the larger trial, where we tested higher ibuprofen doses, explored alternative NSAIDs, and extended follow-up to assess early relapse and post-TB sequelae.

In conclusion, this pilot trial indicates that adjunctive ibuprofen 400 mg once daily during the intensive phase of treatment in adults with pre-XDR/XDR TB was feasible and did not raise major short-term safety concerns. Across multiple inflammatory readouts, we observed immunological trajectories consistent with immunomodulatory activity, but no measurable clinical benefit. These findings, together with the operational lessons generated, support further evaluation of NSAID-based host-directed therapy in adequately powered, placebo-controlled trials with standardised background regimens, including the subsequent SMA-TB RCT (NCT04575519), and exploration of alternative doses and dosing strategies, additional HDT candidates, and combination approaches. Finally, any consideration of incorporating such strategies into clinical recommendations will require robust evidence of clinical benefit and a clearer definition of appropriate patient populations.

## Methods

### Ethics statement

This study was conducted in accordance with the Declaration of Helsinki and all relevant ethical regulations. The study protocol and associated documents (approval code 870/01-17) were reviewed and approved by the Ethics Review Committee of the National Center for Tuberculosis and Lung Diseases (NCTLD), Georgia (IRB00007705, IORG0006411). All study participants received and reviewed the patient information sheet and provided written informed consent prior to enrolment. Participants were not compensated financially or otherwise for their participation in the study.

### Study design and participants

NSAIDS-XDRTB was designed as a phase IIA prospective, interventional, controlled, open-label, pilot trial to estimate the potential efficacy and safety of using adjunctive ibuprofen for the treatment of pre-XDR and XDR-TB (ClinicalTrials.gov NCT02781909). The study protocol and associated documents are publicly available at 10.5281/zenodo.17422437. The trial was conducted in the NCTLD (Georgia).

Eligible participants included males and females aged 16 or older with bacteriologically confirmed pulmonary pre-XDR or XDR-TB, with or without extrapulmonary involvement. Pre-XDR-TB was defined as rifampicin-resistant TB with additional resistance to any fluoroquinolone, while XDR-TB was defined as rifampicin-resistant TB with resistance to any fluoroquinolone and at least one additional Group A drug (bedaquiline or linezolid), in accordance with WHO definitions. Patients were excluded if they had a previous history of TB or major comorbid conditions. Detailed inclusion and exclusion criteria are available in Supplementary Table 1. Before enrolment, all participants provided written informed consent. Sex was recorded as a binary variable (male/female) based on self-report at enrolment. Sex was not prespecified as an analytical variable in the study protocol, given the small sample size and exploratory nature of the pilot; no sex-stratified analyses were carried out. Race and ethnicity were not collected as variables in this study. No analyses were performed based on race, ethnicity, or other socially defined groupings.

### Sample size

As a pilot study, no formal sample size calculation was conducted. The current trial was designed to assess the magnitude and direction of possible responses and was not powered to show smaller differences. The total n per treatment arm was defined based on feasibility, gains in the precision about the mean and variance, and regulatory considerations, according to the literature on pilot studies^26,27^.

### Participant allocation

At diagnosis, participants were enroled and allocated to one of the two experimental groups in a 1:1 ratio. Although the approved protocol specified central concealed randomisation, allocation was implemented at the recruiting site using simple alternation due to an operational implementation error at the single study site (protocol deviation). Site enrolment logs were reviewed and confirmed that the alternating sequence was applied consistently as recorded. Because allocation by alternation is predictable and does not ensure allocation concealment, the study was open-label and selection bias cannot be excluded. No placebo was used, and allocation was therefore unblinded to participants and investigators.

### Procedures and interventions

Participants were categorized as pre-XDR-TB if they had MDR/RR-TB and resistance to any fluoroquinolone. They were categorized as XDR-TB if they met the criteria for pre-XDR-TB and also showed resistance to at least one additional Group A drug, namely bedaquiline or linezolid. Background TB regimens were individualised and tailored using optimised treatment guided by either molecular drug-resistance testing (Line-Probe assays) or phenotypic drug susceptibility testing, in accordance with guidelines for pre-XDR and XDR-TB (Supplementary Table 2). Because regimens were prescribed by the treating physician based on contemporaneous national and WHO guidance during a period of evolving access to newer agents, a uniform BPaL backbone was not implemented in this pilot. Participants were allocated in a 1:1 ratio to receive an individualised background DR-TB regimen alone (control; *n* = 14) or the same background regimen plus ibuprofen 400 mg (one tablet) orally once daily for the first 2 months of therapy (ibuprofen arm; *n* = 14). The study medication was provided by the Pharmacy Department of the NCTLD and administered under directly observed therapy (DOT) according to routine clinical practice. Adherence to ibuprofen was monitored via DOT, and completion or non-completion of the 2-month ibuprofen course is summarised in Supplementary Table 10. From allocation, participants were followed for up to 6 months through scheduled clinical visits and sputum examinations, as per clinical routine (Supplementary Table 3).

### Study Outcomes

The primary efficacy outcome, as prespecified in the protocol, was the proportion of pilot study participants showing clinical and/or microbiological benefits from the new regimen relative to controls, evaluated using three prespecified outcome measurement sets: (i) microbiological (proportion with negative sputum culture at months 2 and 6, and time to stable sputum culture conversion up to month 6); (ii) radiological (chest X-ray changes at months 3 and 6); and (iii) final treatment outcomes per WHO definitions at month 6. Participants were considered cured and successfully treated when showing evidence of bacteriological clearance with no reversion and no evidence of failure. Participants whose treatment was interrupted for two or more consecutive months were considered lost to follow-up^28^. However, the protocol did not define a rule for integrating these measurement sets into a single patient-level endpoint or for assigning an overall benefit. Accordingly, no composite endpoint was constructed, and each measurement set was analysed and reported independently. As a pilot study, all analyses were prespecified as exploratory and intended to indicate direction and magnitude of potential effects rather than to support formal hypothesis testing. Outcome measurements were entered as separate items in the ClinicalTrials.gov registry for administrative reasons; collectively, they constitute the prespecified measurement framework for the primary efficacy outcome, though no formal rule for combining them into a single assignable value was specified.

The secondary outcomes were to compare the proportion of study participants 1) who tolerated the new regimen, 2) showing differences in HQoL, and 3) showing differences in immune responses.

Tolerance of the new regimen, as defined in the protocol, was defined as the absence, over the six-month study period, of any of the following protocol-specified safety events: (1) worsening or exacerbation of TB; (2) conversion from smear-negative to smear-positive or from culture-negative to culture-positive; (3) worsening on chest X-ray; (4) clinically significant vital-sign abnormalities; (5) clinically relevant laboratory test alterations, defined using NCTLD laboratory reference values; or (6) any other event worsening the participant’s health status. Participants experiencing any such event were classified as not tolerating the regimen. Events were recorded at routine study visits, reviewed by the trial clinical team, but were not formally graded for severity. Safety monitoring was performed within the existing clinical governance structure of the NCTLD, specifically the Central Clinical Council for the Treatment of Drug-Resistant Tuberculosis, which oversees MDR/XDR-TB management and safety-related monitoring.

Differences in HQoL and in immune responses were measured as changes detected at months 2 and 6 compared to baseline. Outcome measurements: changes detected in immune responses at months 2 and 6. Immunological studies were assessed in all allocated participants with available samples.

### Demographic and clinical data collection

Once the informed consent was obtained, data from the study were recorded in a case report form (CRF) created ad hoc. The recruiting site used paper source documents to record the data and periodically entered it into an eCRF (REDCap version 12.2.11, hosted at the University of the Witwatersrand, South Africa). Data collected included demographic information, medical history, and concomitant medications. Radiological efficacy was assessed blindly to the treatment group using a scoring system that aggregates all X-ray findings (X-ray score) reported in the CRF. Parenchymal abnormalities, both primary and secondary, along with nodular and pleural abnormalities were considered (Supplementary Table 4). Microbiological confirmation was based on sputum culture, and follow-up culture results were recorded as part of routine care. Baseline smear grading and baseline time-to-positivity were not recorded. TB disease severity was evaluated at baseline using a score adapted from the Bandim TB score^29^ (Supplementary Table 5). The SGRQ and K10 questionnaire were used to measure patients’ health and well-being and psychological distress, through anxiety and depressive symptoms measurements, respectively^13,14^ (Supplementary Tables 6-7).

### Immunological response analysis

Composite inflammation indices were calculated using eCRF data. These indices integrate independent white blood cell subsets, including the NLR, the MLR, and the SII. SII was calculated as follows: neutrophil count × platelet count/lymphocyte count. These indices were selected because they are derived from routine full blood counts available within the programmatic setting and have been previously associated with TB severity and treatment response. C-reactive protein (CRP) was not protocol-specified as it was not routinely available at the study site during the study period. Peripheral whole blood samples were collected at baseline and at months 2 and 6 of treatment. Plasma was isolated from whole blood collected in sodium citrate CPT tubes (BD Vacutainer® CPT^TM^). Whole blood collected in PAXgene® tubes (PreAnalytiX GmbH, Hombrechtikon, Switzerland) were used for the RNA-sequencing analysis. Samples were stored at −80⁰ until analysis.

Levels of circulating pro- and anti-inflammatory cytokines in plasma were assessed in 73 plasma samples from the 28 enroled participants at baseline, months 2 and 6. Levels of TNF, IFNG and IL IL-1B, IL-2, IL-4, IL-6, IL-8, IL-10, IL-12, and IL-17A were measured using Millipore’s MILLIPLEX MAP human high sensitivity T cell magnetic Bead panel (cat#HSTMAG-28SK, EMD Millipore Corporation). This multiplex approach was selected to enable simultaneous measurement of key inflammatory, effector, and immunoregulatory cytokines relevant to TB immunopathogenesis from limited plasma volumes. Assays were performed according to the manufacturer’s instructions, and plates were read using a Luminex® 200 System.

Total RNA was extracted from 73 whole blood samples using the PAXgene Blood miRNA Kit (PreAnalitiX, Hombrechtikon, Switzerland) procedure, according to the manufacturer’s protocol. Total RNA quality was quantified using the Quant-iT Broad-Range dsDNA Assay Kit (Thermo Fisher Scientific), and RNA integrity was assessed using the Fragment Analyzer system HS RNA Kit (15NT) (Agilent). RNA-Seq libraries were prepared with the Illumina Stranded Total RNA Prep with Ribo-Zero Plus (Illumina), following the manufacturer’s recommendations, starting with 0.5 μg of total RNA as the input material. The final library was validated on an Agilent 2100 Bioanalyzer using the DNA 7500 assay. The libraries were sequenced on the NovaSeq 6000 (Illumina) with a read length of 2 × 101 bp following the manufacturer’s protocol for dual indexing. Image analysis, base calling, and quality scoring of the run were performed using the manufacturer’s software Real Time Analysis (RTA v3.4.4), followed by the generation of FASTQ sequence files.

### RNA-Seq data analysis

To identify differentially expressed genes (DEGs) between treatment groups across time points, we used the log-likelihood ratio test implemented in the DESeq2 R package (v1.38.3)^30^. A full model including treatment, time point, and their interaction was compared to a reduced model containing only treatment and time point. DEGs were defined using an adjusted p-value threshold of ≤ 0.05, corrected via the Benjamini-Hochberg method. This approach identified genes whose expression varied significantly between the ibuprofen and control groups over time. Additionally, seven external predictive gene signatures related to TB treatment (Supplementary Table 8) were extracted from the TBSignatureProfiler R package^31^. An enrichment score for each signature was calculated for each sample using single-sample gene set enrichment analysis (ssGSEA).

### Lipid mediators’ analysis

Plasma samples collected at baseline, month 2, and month 6 were analysed by targeted LC‑MS/MS for eight lipid mediators (13‑HODE, 9‑HODE, 15‑HETE, 17‑HETE, 14‑HDoHE, 12‑HETE, 8‑HETE, and 5‑HETE) to capture shifts in arachidonic‑acid metabolism; detailed methods are provided in Supplementary Fig. 5 legend.

### Statistical analysis

All hypothesis tests in this pilot were prespecified as exploratory: p-values are two-sided and reported for descriptive purposes only (not for formal inference), and effect estimates with 95% confidence intervals are presented to indicate direction and magnitude. Both primary and secondary analyses followed an intention-to-treat (ITT) approach, including all allocated participants with available data. Continuous variables are reported as medians and IQR and compared with the Mann–Whitney *U* test; categorical data are presented as counts and percentages. Absolute risk differences with 95% CIs were calculated using the Newcombe method. For radiological outcomes, we calculated ratios at month 3 (M3/BL) and month 6 (M6/BL), considering ratios <1 as favourable. Health-related quality-of-life good responders were defined a priori as SGRQ ≤ 7 and K10 < 20 at both months 2 and 6 (see Supplementary Tables 6-7). Immune response changes were expressed as log2 ratios to baseline and compared with the Mann–Whitney U test. Statistical analyses were performed using GraphPad Prism (version 10.0), R studio v.4.3.1 and Python v3.11.6.

### Reporting summary

Further information on research design is available in the Nature Portfolio Reporting Summary linked to this article.

## Supplementary information

Supplementary Information

Reporting summary

Transparent Peer Review file

## Source data

Source data

## Data Availability

Protocol, including Patient information sheet, Consent form, and Statistical Analysis Plan, is available at 10.5281/zenodo.20342393. RNA-seq data have been deposited in the NCBI Gene Expression Omnibus (GEO) database: GSE298762. Metabolomics data have been deposited in the EMBL-EBI MetaboLights database: MTBLS13376. Individual-level patient data cannot be made available due to patient confidentiality requirements, the conditions of ethics approval granted by the Ethics Review Committee of the National Center for Tuberculosis and Lung Diseases (NCTLD), Georgia, the terms of written informed consent provided by study participants, and applicable data protection legislation. Source data are provided with this paper.
