# Supplementary material for: Adjunctive ibuprofen in pre-extensively drug-resistant and extensively drug-resistant tuberculosis: a phase IIA open-label pilot clinical trial
Source: Nat Commun. 2026 Jul 1;17:8185. doi: 10.1038/s41467-026-75148-9 (PMC13462894; doi:10.1038/s41467-026-75148-9)
Supplement: Supplementary file 1 [file 41467_2026_75148_MOESM1_ESM.pdf]

Supplementary information

Adjunctive ibuprofen in pre-extensively drug-resistant and extensively drug-resistant tuberculosis: a Phase IIA open-label pilot clinical trial

Kaori L. Fonseca[1,2,$], Judith Farrés[1,$], Ketevan Barbakadze[3], Iza Jikia[3], Malkhaz Tsotskhalashvili[3], Juan M. García-Illarramendi [4,5], Lilibeth Arias [1,2], Kennedy Otwombe[6,7], Chiara Sopegno[1,8], Albert Despuig[1], Neil Martinson[6], Nadiia Buhiichyk[1,9], Tamta Korinteli[3], Émilie Layre[10], Jérôme Nigou[10], Zaza Avaliani[3], Nestani Tukvadze[3,11], Sergo Vashakidze[3,12,&], Cristina Vilaplana[1,2,13,&,*]

1. Germans Trias i Pujol Research Institute (IGTP), Badalona, Catalonia, Spain
2. Centro de Investigación Biomédica en Red de Enfermedades Respiratorias (CIBERES), Madrid, Spain
3. National Center for Tuberculosis and Lung Diseases, Tbilisi, Georgia
4. Institute of Biotechnology and Biomedicine, Universitat Autònoma de Barcelona, Barcelona, Spain
5. Vall d'Hebron Institut de Recerca (VHIR), Barcelona, Spain.
6. Perinatal HIV Research Unit (PHRU), Faculty of Health Sciences, University of the Witwatersrand, Johannesburg, South Africa.
7. School of Public Health, Faculty of Health Sciences, University of the Witwatersrand, Johannesburg, South Africa.
8. Universitat Pompeu Fabra, Barcelona, Spain
9. Germans Trias i Pujol Hospital, Fundació Lluita contra la Sida i les Malalties Infeccioses, Barcelona, Spain,
10. Institut de Pharmacologie et de Biologie Structurale (IPBS), Université de Toulouse, CNRS, UPS, Toulouse, France
11. Swiss Tropical and Public Health Institute, Basel, Switzerland
12. The University of Georgia, Tbilisi, Georgia
13. Microbiology Department, Northern Metropolitan Clinical Laboratory, Germans Trias i Pujol University Hospital, Badalona, Catalonia, Spain

[$] These authors contributed equally
[&] These authors jointly supervised this work

Contents

Supplementary Table 1 – Detailed list of the inclusion and exclusion criteria 2

Supplementary Table 2 –Treatments scheme for all enrolled patients 3

Supplementary Table 3 – Summary of planned visits and study procedures. 4

Supplementary Table 4 – NSAIDS X-ray score 5

Supplementary Table 5 – TB Severity score. 5

Supplementary Table 6 – Saint George Respiratory Questionnaire scores in healthy subjects. 5

Supplementary Table 7 –K10 score groupings and categories. 6

Supplementary Table 8 – Gene TB Signatures of treatment failure. 7

Supplementary Table 9 – Laboratory data at baseline 7

Supplementary Table 10 – Treatment status by visit, treatment completion and inclusion in the time-to-stable-culture-conversion (tSCC) analysis. 8

Supplementary Table 11 – Efficacy Scores per study group 9

Supplementary Figure 1. Radiological, HQoL and clinical changes a long time relative to baseline per experimental group. 10

Supplementary Figure 2. Relative change over time in blood cell-derived inflammation index parameters compared to baseline. 11

Supplementary Figure 3. Relative change over time in plasma biomarkers associated with inflammation compared to baseline. 12

Supplementary Figure 4. Relative change over time in signature scores derived from whole blood RNA transcripts, compared to baseline. 13

Supplementary Figure 5. Relative change over time in lipid mediators measured in plasma samples, compared to baseline. 14

References 15

Protocol and Statistical Analysis Plan 16

Supplementary Table 1 – Detailed list of the inclusion and exclusion criteria

INCLUSION CRITERIA	EXCLUSION CRITERIA
1. Females and males aged ≥ 16<br>2. The patient must provide written informed consent<br>3. Females of childbearing potential (including females less than 2 years postmenopausal) must have a negative pregnancy test at enrolment and must agree to use highly effective methods of birth control<br>4. *M. tuberculosis* (Mtb) detected by culture with available drug susceptibility results for current isolate.<br>5. New confirmed pre-XDR and XDR- TB confirmed by drug susceptibility testing (DST, or any other drug susceptibility test used as per routine in the NCTLD laboratory).	1. Inability to provide written informed consent<br>2. First line drug treatment susceptible Mtb strain<br>3. Pregnancy/Breastfeeding at inclusion<br>4. Any of the following laboratory parameters: Aspartate aminotransferase (AST) or alanine aminotransferase (ALT) > 5 x upper limit of normal (ULN); total bilirubine > 2 x ULN; estimated glomerular filtration rate (eGFR) <60ml/hr; Neutrophil count ≤ 500 neutrophils / mm3; Platelet count < 50,000 cells / mm3<br>5. Receiving or anticipated to receive a daily dose of ≥ 10 mg of systemic prednisone or equivalent within the period starting 14 days prior to enrolment, or > 5 doses per week of any NSAID for ≥2 weeks in the month prior to randomisation.<br>6. History of sensitivity or allergy to ibuprofen.<br>7. A history suggestive of any of the following in past two years: peptic ulcer disease, gastro-intestinal bleeding, coagulopathy, or renal disease requiring hospitalisation, history of severe cardiovascular disease or symptoms suggestive of severe cardiovascular disease.<br>8. Co-treatment in the three months prior to randomisation with anticoagulant therapy or immune modulating therapy (cancer treatments, oral or inhaled steroids; etc.).<br>9. Patients with HIV infection<br>10. Alcohol use: Drinks more than an average of three units/day over a usual week's consumption.<br>11. Any other major co-morbid conditions precluding full evaluation or any other finding which in the opinion of the investigator would compromise the protocol compliance or significantly influence the interpretation of results.

Supplementary Table 2 –Treatments scheme for all enrolled patients

Patient ID	Study group	TB drug sensitivity	Treatment scheme
NSAIDS-XDR-TB-CT-1	Control	Pre-XDR	Cm+Pto+Cs+Bdq+Lzd+Cfz
NSAIDS-XDR-TB-CT-2	Control	XDR	Z+Cs+Bdq+Cfz+Imp+Amx/Clv
NSAIDS-XDR-TB-CT-3	Control	XDR	Cfz+Dlm+Imp+Amx/Clv+Km
NSAIDS-XDR-TB-CT-4	Control	XDR	Z+Cm+Cs+Bdq+Lzd+PAS
NSAIDS-XDR-TB-CT-5	Control	Pre-XDR	Z+Cm+Pto+Cs+Mfx+PAS
NSAIDS-XDR-TB-CT-6	Control	XDR	Cm+Pto+Cs+Lzd +Cfz+Dlm
NSAIDS-XDR-TB-CT-7	Control	Pre-XDR	Z+Pto+Cs+Bdq+Lzd+Mfx
NSAIDS-XDR-TB-CT-8	Control	Pre-XDR	Z+Cm+Pto+Cs+Lfx
NSAIDS-XDR-TB-CT-9	Control	Pre-XDR	Z+Cm+Pto+Cs+Bdq+Lzd
NSAIDS-XDR-TB-CT-10	Control	XDR	Pto+Cs+Bdq+Lzd+Cfz
NSAIDS-XDR-TB-CT-11	Control	XDR	Z+Cm+Cs+Lzd+Cfz+Dlm
NSAIDS-XDR-TB-CT-12	Control	XDR	Z+Pto+Cs+Cfz
NSAIDS-XDR-TB-CT-13	Control	XDR	Cm+Pto+Cs+Lzd+Cfz+Dlm
NSAIDS-XDR-TB-CT-14	Control	XDR	Bdq+Lzd+Dlm+Imp+Amx/clv
NSAIDS-XDR-TB-IBU-1	Ibuprofen	XDR	Cm+Pto+Cs+Bdq+Lzd+Cfz
NSAIDS-XDR-TB-IBU-2	Ibuprofen	XDR	Cm+Cs+Lzd+Cfz+Dlm+lmp+Amx/Clv
NSAIDS-XDR-TB-IBU-3	Ibuprofen	XDR	Pto+Cs+Cfz+Dlm+Imp
NSAIDS-XDR-TB-IBU-4	Ibuprofen	Pre-XDR	Z+E+Cm+Cs+Bdq+Lzd
NSAIDS-XDR-TB-IBU-5	Ibuprofen	XDR	Cm+Cs+Imp+Amx/Clv+PAS
NSAIDS-XDR-TB-IBU-6	Ibuprofen	XDR	Cs+Bdq+Lzd+Cfz+Imp+Amx/Clv+Mfx
NSAIDS-XDR-TB-IBU-7	Ibuprofen	Pre-XDR	Z +Cm+Pto+Cs+Mfx
NSAIDS-XDR-TB-IBU-8	Ibuprofen	XDR	Cm+Pto+Cs+Bdq+Lzd
NSAIDS-XDR-TB-IBU-9	Ibuprofen	XDR	Cm+Pto+Cs+Bdq+Lzd+Cfz
NSAIDS-XDR-TB-IBU-10	Ibuprofen	XDR	Z+Pto+Cs+Lzd+Cfz+Imp+Amx/Clv
NSAIDS-XDR-TB-IBU-11	Ibuprofen	XDR	Z+Pto+Cs+Lzd+Cfz+Dlm
NSAIDS-XDR-TB-IBU-12	Ibuprofen	XDR	Z+Cm+Pto+Cs+Bdq+Lzd
NSAIDS-XDR-TB-IBU-13	Ibuprofen	XDR	Cm+Pto+Bdq+Lzd+Cfz
NSAIDS-XDR-TB-IBU-14	Ibuprofen	Pre-XDR	Z+Cm+Pto+Cs+Lzd+Cfz+Lfx

Amx/Clv, amoxicillin/clavulanate; **Bdq**, bedaquiline; **E**, ethambutol; **Cfz**, clofazimine; **Cm**, capreomycin; **Cs**, cycloserine; **Dlm**, delamanid; **Km**, kanamycin; **Imp**, imipenem; **Lfx**, levofloxacin; **Lzd**, linezolid; **Mfx**, moxifloxacin; **PAS**, para-aminosalicylic acid; **Pto**, pretomanid; **Z,** pyrazinamide.

Supplementary Table 3 – Summary of planned visits and study procedures.

	Baseline	Follow up					
Month	0	1	2	3	4	5	6
Visit	1	2	3	4	5	6	7
Informed consent	X	..	..	..	..	..	..
Inclusion	X	..	..	..	..	..	..
Clinical examination	X	X	X	X	X	X	X
Sputum culture	X	X	X	X	X	X	X
Rx assay	X	..	..	X	..	..	X
Blood tests	X	X	X	X	X	X	X
Adverse events recording	X	X	X	X	X	X	X
HQoL questionnaires	X	..	X	..	..	..	X
Sample collection for evaluation of immune responses	X	..	X	..	..	..	X
Interim treatment outcomes assessment	..	..	..	X	..	..	X
Final outcome assessment	..	..	..	X	..	..	X

HQoL, Health Quality of Life; Rx, radiological.

Supplementary Table 4 – NSAIDS X-ray score

Score variables	Signs	Scores
Primary Parenchymal abnormalities	Cavities, fibrosis, infiltrates (left, right)	Sum events. Max 8 points
Secondary parenchymal abnormalities	Cavities, fibrosis, infiltrates (left, right)	Sum events. Max 8 points
Nodular abnormalities		Nodule size:<br>• Small – 1<br>• Medium – 2<br>• Big – 3
Pleural abnormalities		Presence – 1<br>Absence – 0
X-ray sum	Sum all scores	Max 20

Xray, a score that accounted for key radiological signs, including primary and secondary parenchymal abnormalities, nodular lesions, and pleural abnormalities.

Supplementary Table 5 – TB Severity score.

This score is an adaptation of the Bandim TB score first introduced in a 2008 study by Wejse et al.[1] no mid-upper arm circumference measurements and positive finding at lung auscultation were available in our study.

Score variables	Score
Cough	1
Haemoptysis	1
Shortness of breath	1
Chest pain	1
Night sweats	1
Anaemia	1
Pulse > 90 beats/min	1
Temperature > 37ºC	1
BMI < 18	1
BMI < 16	1
Max number of points	10

BMI, Body mass index.

Supplementary Table 6 – Saint George Respiratory Questionnaire scores in healthy subjects.

SGRQ component	Mean (95% CI) SGRQ normal subjects
Symptoms score	12 (9-15)
Activity score	9 (7-12)
Impacts score	2 (1-3)
Total score	6 (5-7)

From Jones et al.[2] (2). SGRQ, Saint George Respiratory Questionnaire.

Supplementary Table 7 –K10 score groupings and categories.

K10 Total Score Levels	Level of psychological distress
10-19	are likely to be well
20-24	are likely to have a mild mental disorder
25-29	are likely to have a moderate mental disorder
30-50	are likely to have a severe mental disorder

K10, Kessler Psychological Distress Scale. The 2001 Victorian Population Health Survey[3] adopted a set of cut-off scores that may be used as a guide for screening for psychological distress.

Supplementary Table 8 – Gene TB Signatures of treatment failure.

Signature name	Gene names	Efficacy criteria
Heycken_FAIL_22[4]	CD274, FAM20A, LPCAT2, TRIM27, GYG1, HIST1H1B, RPAP3, A_33_P3281041, BATF2, C2, GK, IFIT2, IFITM1, KREMEN1, PDE4D, GBP5, IL27, KCNJ2-AS1, SERPING, STAT1, TNFRSF21, VAMP5	Negative *M. tuberculosis* culture status at 6 months after treatment initiation without positive cultures thereafter and no disease recurrence during the follow-up period of 1 year after therapy end.
Long_RES_10[5]	CD274, KIF1B, IL15, TLR1, TLR5, FCGR1A, GBP1, NOD2, GBP2, EGF	Response criteria defined in the Thompson TB population.
Tabone_RES_25[6]	IFITM1, UBE2L6, EFCAB2, DUSP3, VAMP5, SEPT4, RP11-468E2.5, PDCD1LG2, SOCS1, IRF1, APOL1, ANXA3, POLB, SORT1, TIMM10, RARRES3, CASP5, CD274, FCER1G, TIFA, PML, GPR84, PSME2, RP11-476010.1, APOL6	Response criteria defined in the Thompson TB population.
Tabone_RES_27[6]	BAK1, SCARF1, ICAM1, LIMK1, SEPT4, FAM20A, CD274, NRN1, IGF2BP3, CASP5, TIFA, FCGR1A, GBP5, C1QC, SDC3, CDCP1, C1QB, P2RY14, GRAMD1C, GBP6, SOCS3, PRTN3, PDCD1LG2, FCGR1B, GRIN3A, GBP1P1, FCGR1CP	Response criteria defined in the Thompson TB population.
Thompson_FAIL_13[7]	ATP1A1, CXorf40B, DARS2, EXOC2, EMC1, KIAA0100, VWA8, MAP7D3, PSMD1, SEC31A, SND1, STT3A, UCP2	Proven and maintained sputum culture negativity by month 6.
Thompson_RES_5[7]	SMARCD3, UCP2, MAP7D3, STT3A, RP11-295G20.2	Proven and maintained sputum culture negativity by month 6.
Zimmer_RES_3[8]	GBP5, DUSP3, KLF2	Negative microbiological test by month 6 or latest month 12 and improved or resolved symptoms.

Supplementary Table 9 – Laboratory data at baseline

Variables	Overall Average (Std.Dev.)	Control Average (Std.Dev.)	Ibuprofen Average (Std.Dev.)	P-Value
Segmented neutrophiles (50-70%)	77.4 (5.3)	76.4 (6.0)	78.4 (4.5)	0.33
Monocytes (4-9%)	4.9 (1.4)	4.7 (1.5)	5.1 (1.3)	0.51
Platelets (140-440x109/1)	382.3 (113.6)	376.6 (136.6)	388.5 (87.7)	0.79
Lymphocytes count (18-40%)	17.6 (4.5)	19.0 (4.6)	16.27 (4.1)	0.11
Hb - Men (14-18g/dL) -Women (12-15g/dL)	125.3 (18.6)	129.8 (20.0)	120.8 (16.6)	0.21
ESR - Men (< 15mm/hour) - Women (< 20mm/hour)	37.9 (14.3)	34.21 (17.7)	41.9 (8.4)	0.17
Total protein (6,5-8,0 gr/dL)	6.3 (0.6)	6.27 (0.7)	6.40 (0.6)	0.74
Albumin (40-60%)	32.3 (5.6)	34.58 (4.8)	29.7 (5.3)	0.03
Glucose (4,2-6,4mmol/L)	5.4 (3.6)	4.5 (0.7)	6.5 (5.2)	0.18
K^+ (3.3-5.1mmol/L)	4.5 (0.6)	4.4 (0.5)	4.6 (0.6)	0.46
Creatinine – Men (50-120mkmol/L)-Women (40-90mkmol/L)	75.4 (14.2)	74.8 (13.7)	75.9 (15.2)	0.84
Total bilirubin (8-20mkmol/L)	9.3 (3.3)	9.1 (3.0)	9.4 (3.6)	0.82
ALT - Men (0-42v/L), Women (0-37v/L)	18.1 (10.8)	18.6 (9.0)	17.6 (12.6)	0.81
AST - Men (0-37v/L) - Women (0-31v/L)	20.7 (15.8)	19.9 (7.8)	21.6 (21.4)	0.78

Standard deviation (Std.Dev.). Hemoglobin (Hb), Erythrocytes sedimentation rate (ESR), Alanine transaminase (ALT), Aspartate transferase (AST). P-value corresponds to two-sided independent samples t-test, comparing Ibuprofen respect to Control.

Supplementary Table 10 – Treatment status by visit, treatment completion and inclusion in the time-to-stable-culture-conversion (tSCC) analysis.

Study group	Subject ID	Final status	Included in the tSCC analysis	tSCC
**Control**	NSAIDS-XDR-TB-CT-1	TT concluded	Yes	2
**Control**	NSAIDS-XDR-TB-CT-2	TT concluded	Yes	4
**Control**	NSAIDS-XDR-TB-CT-3	TT concluded	Yes	4
**Control**	NSAIDS-XDR-TB-CT-4	TT concluded	Yes	4
**Control**	NSAIDS-XDR-TB-CT-5	TT concluded	Yes	3
**Control**	NSAIDS-XDR-TB-CT-6	LoF-U (unknown reason, missed last visit)	Yes	3
**Control**	NSAIDS-XDR-TB-CT-7	LoF-U (left the country)	No	NA
**Control**	NSAIDS-XDR-TB-CT-8	TT concluded	Yes	6
**Control**	NSAIDS-XDR-TB-CT-9	TT concluded	Yes	2
**Control**	NSAIDS-XDR-TB-CT-10	LoF-U (left the country)	No	NA
**Control**	NSAIDS-XDR-TB-CT-11	Died	No	NA
**Control**	NSAIDS-XDR-TB-CT-12	TT concluded	Yes	1
**Control**	NSAIDS-XDR-TB-CT-13	TT concluded	Yes	4
**Control**	NSAIDS-XDR-TB-CT-14	TT concluded	Yes	5
**Ibuprofen**	NSAIDS-XDR-TB-IBU-1	Ibu completed, TT concluded	Yes	4
**Ibuprofen**	NSAIDS-XDR-TB-IBU-2	Ibu completed, TT concluded	Yes	4
**Ibuprofen**	NSAIDS-XDR-TB-IBU-3	Ibu completed, TT concluded	Yes	3
**Ibuprofen**	NSAIDS-XDR-TB-IBU-4	Ibu completed, TT concluded	Yes	3
**Ibuprofen**	NSAIDS-XDR-TB-IBU-5	Ibu completed, LoF-U (left the country)	No	NA
**Ibuprofen**	NSAIDS-XDR-TB-IBU-6	Ibu completed, TT concluded	Yes	4
**Ibuprofen**	NSAIDS-XDR-TB-IBU-7	Ibu not completed, LoF-U (abandoned treatment for family conditions)	No	NA
**Ibuprofen**	NSAIDS-XDR-TB-IBU-8	Ibu completed, LoF-U (left the country)	No	NA
**Ibuprofen**	NSAIDS-XDR-TB-IBU-9	Ibu completed, LoF-U (incarcerated)	Yes	2
**Ibuprofen**	NSAIDS-XDR-TB-IBU-10	Ibu completed, TT concluded	Yes	5
**Ibuprofen**	NSAIDS-XDR-TB-IBU-11	Ibu completed, TT concluded	Yes	4
**Ibuprofen**	NSAIDS-XDR-TB-IBU-12	Ibu completed, TT concluded	Yes	3
**Ibuprofen**	NSAIDS-XDR-TB-IBU-13	Ibu completed, TT concluded	Yes	3
**Ibuprofen**	NSAIDS-XDR-TB-IBU-14	Ibu completed, TT concluded	Yes	4

TT, treatment; LoF-U, lost to follow-up; tSCC, time to stable sputum conversion; NA, non-applicable.

Supplementary Table 11 – Efficacy Scores per study group

Efficacy Scores	Control	Ibuprofen	Mann-Whitney *U* test (p-value, two-sided test)
Microbiological efficacy-related events (N evaluated)	11	11	
Median Stable sputum conversion months (IQR)	4 (2.5 – 4)	4 (3 – 4)	0.95
Radiological efficacy-related events M3 (N evaluated)	11	11	
Median X-ray score (IQR)	5 (4 – 5.5)	6 (4 – 6)	0.40
Radiological efficacy-related events M6 (N evaluated)	*10*	*11*	
Median X-ray score (IQR)	1 (0 – 1)	1 (1 – 1.8)	0.44
Health Quality of Life (HQoL) M2 (N evaluated)	12	13	
Median SGRQ score (IQR)	35 (16 – 71)	64 (53 – 69)	0.20
Median K10 score (IQR)	23 (17 – 29)	30 (25 – 30)	0.05
Health Quality of Life (HQoL) M6 (N evaluated)	*10*	*10*	
Median SGRQ score (IQR)	5 (1 – 17)	14 (3 – 24)	0.28
Median K10 score (IQR)	12 (12 – 13)	13 (12 – 13)	0.19

IQR, Inter-quartile range; M2, month 2; M3, month 3; M6, month 6. X-ray score, a score that accounted for key radiological signs, including primary and secondary parenchymal abnormalities, nodular lesions, and pleural abnormalities. SGRQ, Saint George Respiratory questionnaire; K10, Kessler Psychological Distress Scale. Statistical analysis was performed using the Mann-Whitney U test (two-sided) comparing ibuprofen to the control group. *P* values are reported as exact values. No adjustments were made for multiple comparisons.

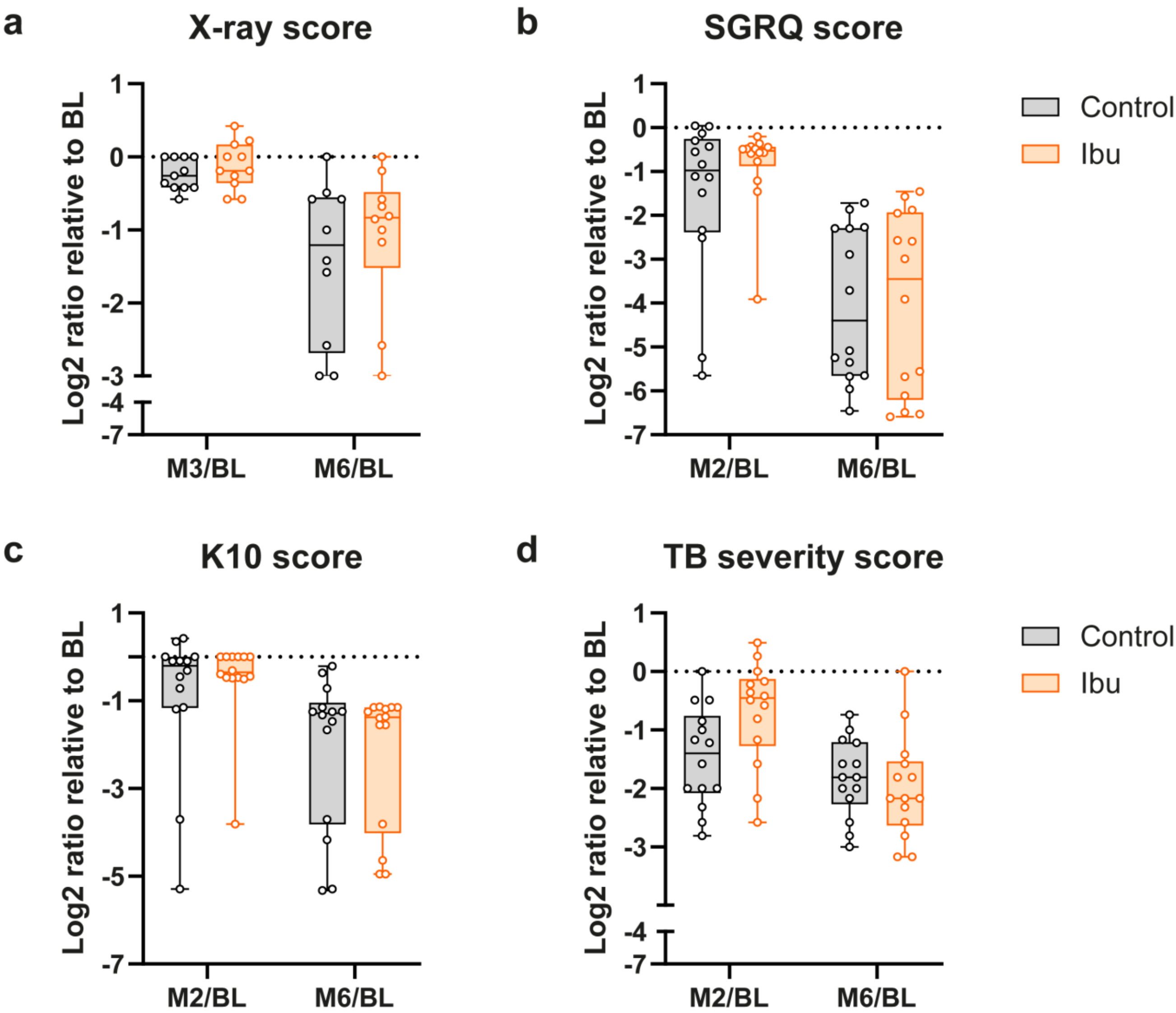

Supplementary Figure 1. Radiological, HQoL and clinical changes a long time relative to baseline per experimental group.

Panel a) X-ray score ratio assessed at month 3 and 6 after treatment initiation, relative to the baseline. Panel **b)** SGRQ score ratio assessed at month 2 and 6 after treatment initiation, relative to the baseline and **c)** K10 score ratio at month 2 and 6 after treatment initiation, relative to the baseline. **d)** TB severity score ratio assessed at month 2 and 6 after treatment initiation, relative to the baseline.

Boxplots depict the median (centre line), interquartile range (IQR; box boundaries represent the 25th and 75th percentiles) and minimum/maximum values (whiskers extending to the smallest and largest observed values). Each dot represents the log2 ratio for an individual patient. Statistical analysis was performed using unpaired two-tailed Mann-Whitney test. A zero-reference line indicates no change from baseline. Orange, ibuprofen group; grey, control group. Sample sizes per panel and time point are detailed in the Source Data file.

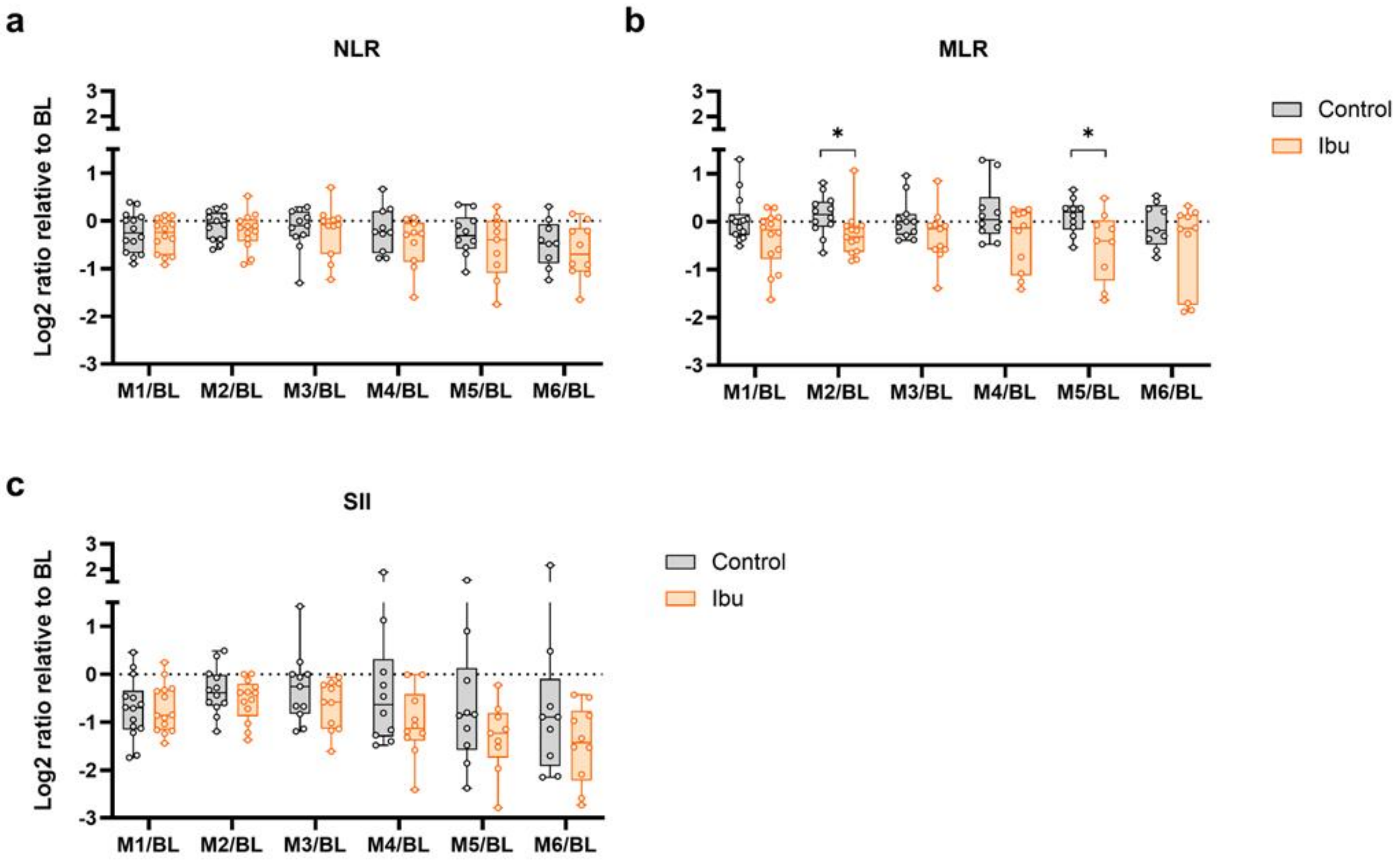

Supplementary Figure 2. Relative change over time in blood cell-derived inflammation index parameters compared to baseline.

Panel a) Neutrophil to lymphocyte ratio (NLR). Panel **b)** Monocyte to lymphocyte ratio (MLR). Panel **c)** Systemic immune-inflammation index (SII) platelet x NLR.

Ratio at month 2 and 6 after treatment initiation, relative to the baseline. Boxplots display the median (centre line), interquartile range (IQR; box boundaries represent the 25th and 75th percentiles), and minimum/maximum values (whiskers extending to the smallest and largest observed values). Each dot represents the log2 ratio for an individual patient. Statistical analysis was performed using an unpaired two-tailed Mann-Whitney test. A zero-reference line indicates no change from baseline. Reported p-values are unadjusted and exploratory and are provided for descriptive purposes only. Orange, ibuprofen group; grey, control group. Number of samples are listed in Figure4a of the paper.

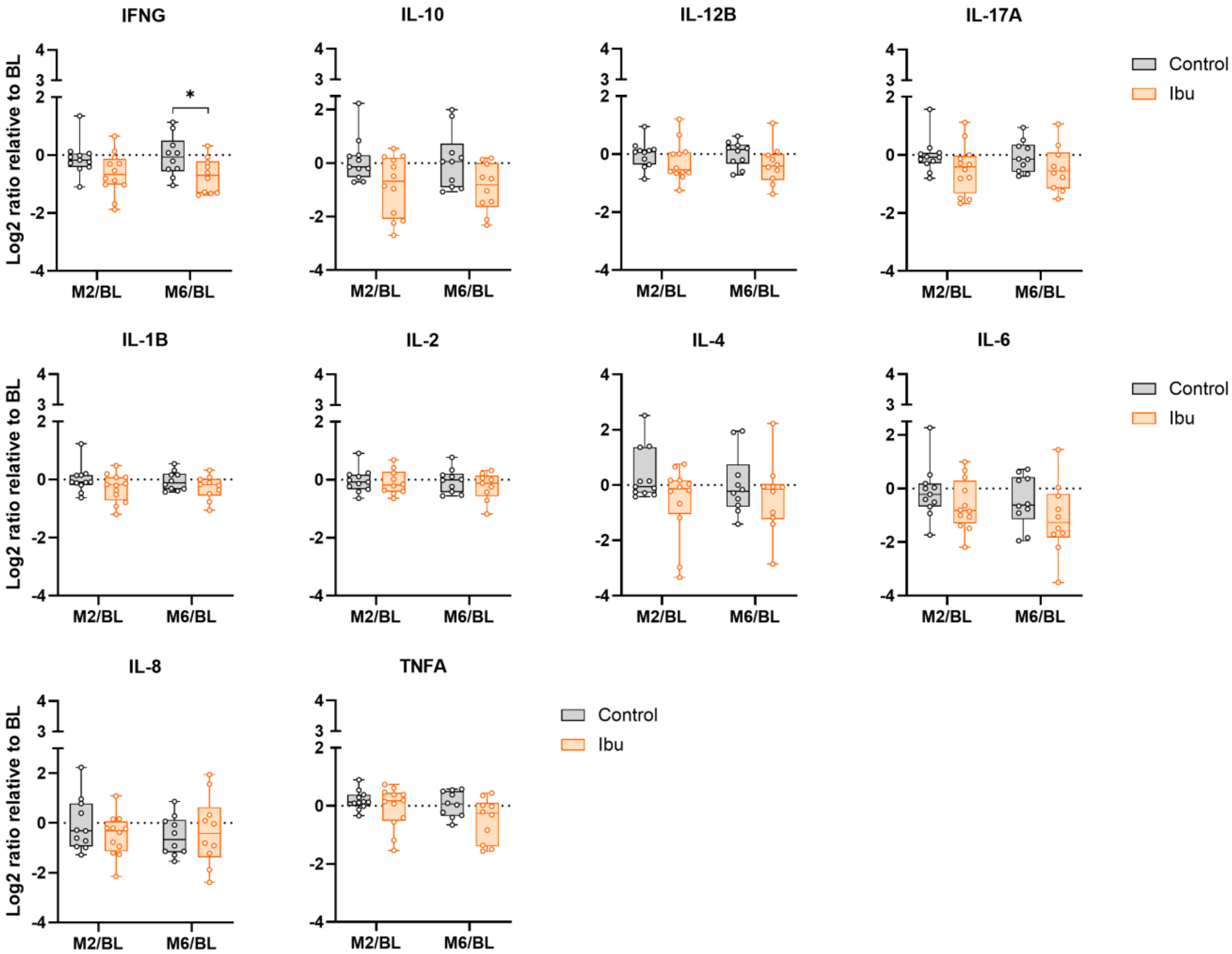

Supplementary Figure 3. Relative change over time in plasma biomarkers associated with inflammation compared to baseline.

Ratio at month 2 and 6 after treatment initiation, relative to the baseline. Boxplots display the median (centre line), interquartile range (IQR; box boundaries represent the 25th and 75th percentiles), and minimum/maximum values (whiskers extending to the smallest and largest observed values). Each dot represents the log2 ratio for an individual patient. Statistical analysis was performed using an unpaired two-tailed Mann-Whitney test. A zero-reference line indicates no change from baseline. Reported p-values are unadjusted and exploratory and are provided for descriptive purposes only. Orange, ibuprofen group; grey, control group. Number of samples are listed in Figure4b of the paper.

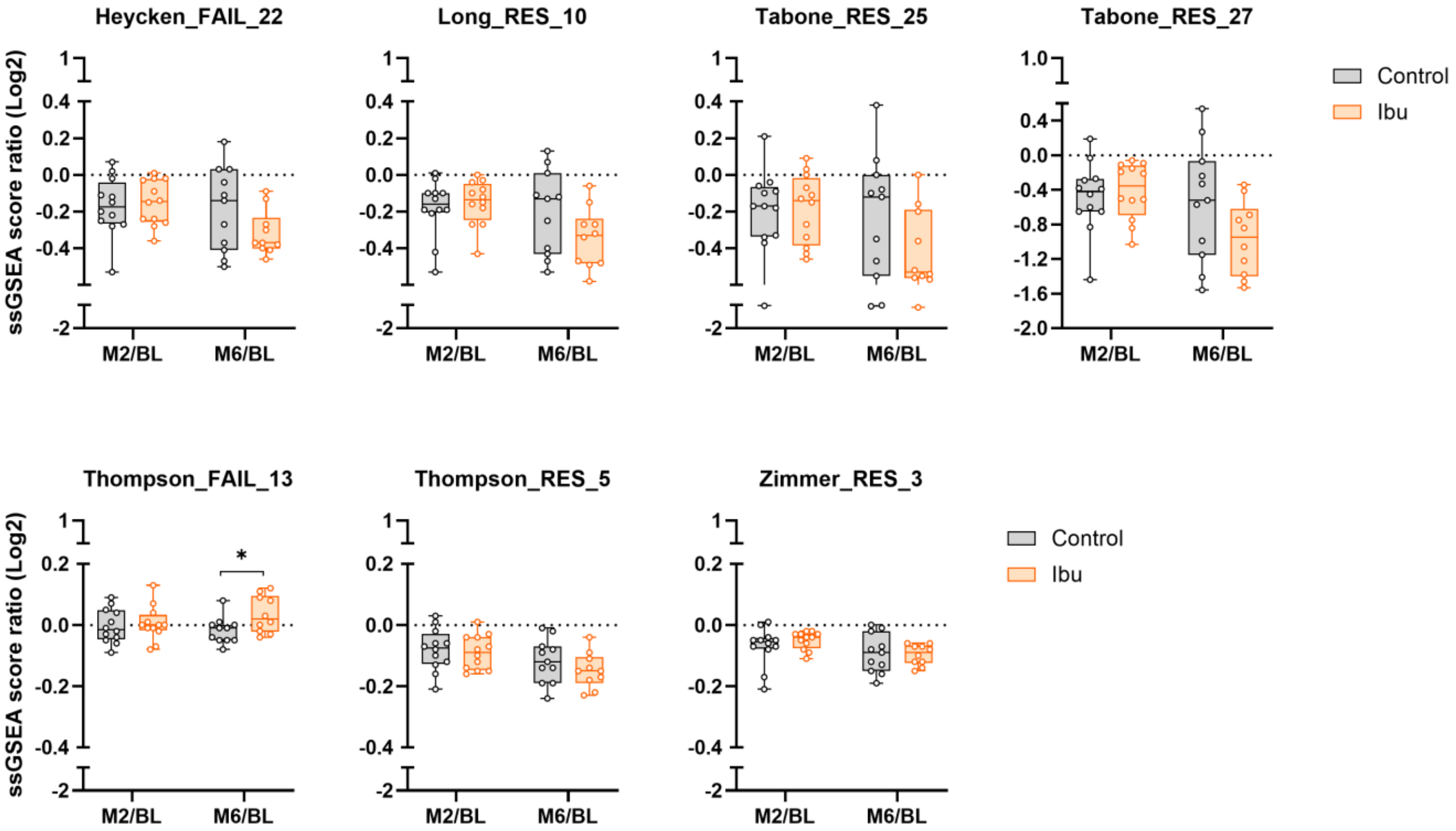

Supplementary Figure 4. Relative change over time in signature scores derived from whole blood RNA transcripts, compared to baseline.

Using the ssGSEA algorithm, we computed enrichment scores for seven published TB signature gene sets that predict the risk of treatment failure. Changes in these scores over time -expressed as the ratio at months 2 and 6 after treatment initiation relative to baseline- were compared between experimental groups. Boxplots depict the median (centre line), interquartile range (IQR; box boundaries represent the 25th and 75th percentiles) and minimum/maximum values (whiskers extending to the smallest and largest observed values). Each dot represents the log2 ratio for an individual patient. Statistical analysis was performed using an unpaired two-tailed Mann-Whitney test. A zero-reference line indicates no change from baseline. For all signatures, a decrease in score indicates a better response, except for *Thompson_FAIL_13*, where an increase in score indicates a better response. Reported p-values are unadjusted and exploratory and are provided for descriptive purposes only. Orange, ibuprofen group; grey, control group. Number of samples are listed in Figure4c of the paper.

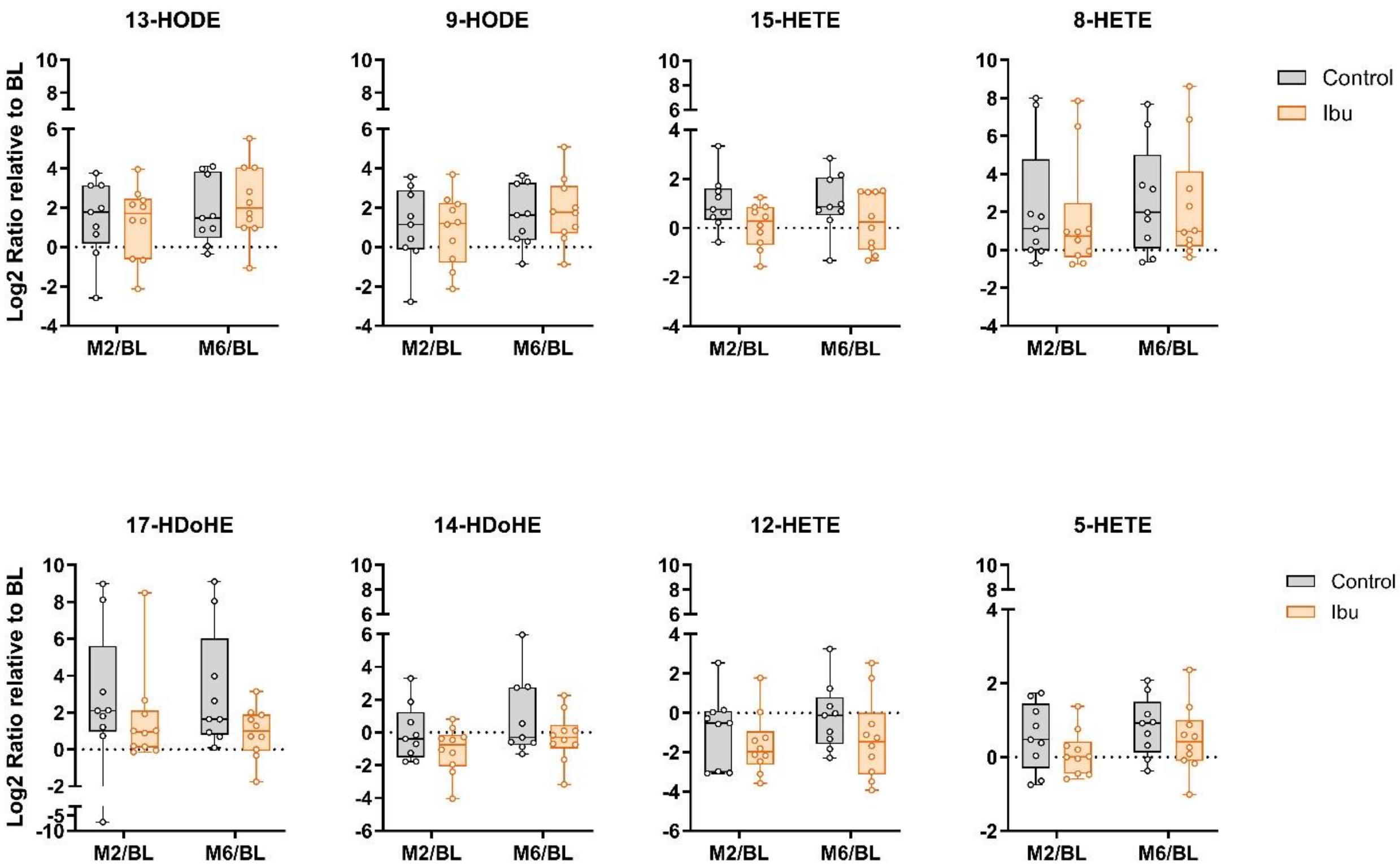

Supplementary Figure 5. Relative change over time in lipid mediators measured in plasma samples, compared to baseline.

Plasma concentrations of eight eicosanoids were quantified using liquid chromatography–mass spectrometry (LC–MS). Eicosanoid concentrations in plasma from patients were analysed at baseline, month 2 and month 6. Quantification of 5 – hydroxyeicosatetraenoic acid (HETE), 8-, 12, 15 and 17 – HETE, 9-hydroxyoctadecadienoic acid (HODE), 13 – HODE, 14 Hydroxydocosahexaenoic acid (HDoHe) using liquid chromatography – mass spectrometry (LC-MS) was performed as previously described (9) at the MetaToul Lipidomic Core Facility (I2MC, Inserm 1048, Toulouse, France, MetaboHUBANR-11-INSB- 0010). Briefly, methanol and internal standard (Deuterium labeled compounds) were added before centrifugation (2000 g for 15 min at 4°C). Supernatants were transferred into 96-well deep plates and diluted in H2O. Samples were then submitted to solid phase extraction (SPE) using OASIS HLB 96-well plate (30 mg/well, Waters) and reconstituted in MeOH. Lipid mediators were separated on a ZorBAX SB-C18 column (Agilent Technologies) using Agilent 1290 Infinity HPLC system (Technologies) coupled to an ESItriple quadruple G6460 mass spectrometer (Agilent Technologies). Data were acquired in Multiple Reaction Monitoring (MRM) mode with optimized conditions (ion optics and collision energy). Peak detection, integration and quantitative analysis were done using Mass Hunter Quantitative analysis software (Agilent Technologies) based on calibration lines built with commercially available eicosanoids standards (Cayman Chemicals).

Longitudinal changes in 5-HETE, 8-, 12-, 15-, and 17-HETE, 9-HODE, 13-HODE, and 14-HDoHE were assessed. Data are presented as ratio at months 2 and 6 relative to baseline and were compared between experimental groups. Boxplots depict the median (centre line), interquartile range (IQR; box boundaries represent the 25th and 75th percentiles) and minimum/maximum values (whiskers extending to the smallest and largest observed values). Each dot represents log2 ratio for an individual patient. Statistical analysis was performed using an unpaired two-tailed Mann-Whitney test. A zero-reference line denotes no change from baseline. Orange, ibuprofen group; grey, control group.

References

1. Wejse, C. *et al.* TBscore: Signs and symptoms from tuberculosis patients in a low-resource setting have predictive value and may be used to assess clinical course. *Scandinavian Journal of Infectious Diseases* **40**, 111–120 (2008).
2. Jones, P. W., Quirk, F. H., Baveystock, C. M. & Littlejohns, P. A self-complete measure of health status for chronic airflow limitation. The St. George's Respiratory Questionnaire. *Am Rev Respir Dis* **145**, 1321–7 (1992).
3. Kessler, R. C. *et al.* Short screening scales to monitor population prevalences and trends in non-specific psychological distress. *Psychological medicine* **32**, 959–976 (2002).
4. Heyckendorf, J. *et al.* Prediction of anti-tuberculosis treatment duration based on a 22-gene transcriptomic model. *Eur Respir J* **58**, (2021).
5. Long, N. P. *et al.* A 10-gene biosignature of tuberculosis treatment monitoring and treatment outcome prediction. *Tuberculosis (Edinb)* **131**, 102138 (2021).
6. Tabone, O. *et al.* Blood transcriptomics reveal the evolution and resolution of the immune response in tuberculosis. *Journal of Experimental Medicine* **218**, e20210915 (2021).
7. Thompson, E. G. *et al.* Host blood RNA signatures predict the outcome of tuberculosis treatment. *Tuberculosis (Edinb)* **107**, 48–58 (2017).
8. Zimmer, A. J. *et al.* A novel blood-based assay for treatment monitoring of tuberculosis. *BMC Res Notes* **14**, 247 (2021).

Protocol and Statistical Analysis Plan

CLINICAL TRIAL PROTOCOL

“Pilot Study to estimate the potential efficacy and safety of using Adjunctive Ibuprofen for the Treatment of pre-XDR and XDR Tuberculosis”.

23th October 2017

Version 3

23th October 2017

Code: NSAIDS-XDR-TB

PROTOCOL TITLE: **"Pilot Study to estimate the potential efficacy and safety of using Adjunctive Ibuprofen for the Treatment of XDR Tuberculosis".**

**Protocol ID**	**NSAIDS-XDR-TB**
**Short title**	**"Adjunctive Ibuprofen for pre-XDR and XDR Tuberculosis"**
**Version**	**3**
**Date**	**23th October 2017**
**Coordinating investigator/Principal Investigator**	**Cristina Vilaplana, MD PhD**<br>Fundació Institut Germans Trias i Pujol. Unitat de Tuberculosi Experimental. Crtra. De Can Ruti, Camí de les Escoles, ed. Recerca. 08916, Badalona. Catalonia. Spain. Tel.: +34630288933. Email: cvilaplana@gmail.com; cvilaplana@igtp.cat
**Trial Site Investigator Responsible**	Sergo Vashakidze,MD PhD<br>National Center for Tuberculosis and Lung Diseases<br>50, Maruashvili Str., Tbilisi, 0101, Georgia<br>Tel: +995 599 23 65 53; Email: sergovashakidze@yahoo.com
**Sponsor**	Fundació Institut Germans Trias i Pujol. Unitat de Tuberculosi Experimental. Institut Germans Trias i Pujol. Crtra. De Can Ruti, Camí de les Escoles, ed. Recerca. 08916, Badalona. Catalonia. Spain.
**Independent expert**	Dr. Neil Martinson<br>Perinatal HIV Research Unit<br>Chris Hani Baragwanath Hospital<br>P.O. Box 114, Diepkloof, 1864<br>Soweto, South Africa<br>Tel: +27 11 989 9700<br>Email: martinson@phru.co.za
**External Laboratory sites**	Routine evaluations will be conducted at NCTLD

	facilities as per routine, coordinated by Dr. Natalia Schubladze, National Center for Tuberculosis and Lung Diseases; 50, Maruashvili Str., Tbilisi, 0101, Georgia;Tel:+995599236553;Email: nata.64@gmail.com<br><br>Dr Cristina Vilaplana will be the responsible of the evaluation of the immune responses: Unitat de Tuberculosi Experimental. Fundació Institut Germans Trias i Pujol. Crtra. De Can Ruti, Camí de les Escoles, ed. Recerca. 08916, Badalona. Catalonia. Spain. Dr Cristina Vilaplana. Tel.: +34934978677. Email: cvilaplana@gmail.com; cvilaplana@igtp.cat<br><br>These analysis of the immune responses will be done in collaboration with Pr Helen Fletcher, Senior Lecturer in Immunology, London School of Hygiene & Tropical Medicine, Immunology and Infection Department, Keppel Street, London, W1CE 7HT.

PROTOCOL SIGNATURE SHEET

Name	Signature	Date
**Sponsor or legal representative:**<br>Fundació Institut Germans Trias i Pujol. Unitat de Tuberculosi Experimental.		
**[Coordinating Investigator/ Principal Investigator]:**<br>Dr. Cristina Vilaplana<br>Unitat de Tuberculosi Experimental<br>Fundació Institut Germans Trias i Pujol. Unitat de Tuberculosi Experimental.		
**Co-investigators:**		
**Trial Site Investigator Responsible:**<br>Dr. Sergo Vashakidze,<br>National Center for Tuberculosis and Lung Diseases<br>50, Maruashvili Str., Tbilisi, 0101, Georgia		
Study Doctor:<br>Dr. Ketevan Barbakadze<br>National Center for Tuberculosis and Lung Diseases<br>50, Maruashvili Str., Tbilisi, 0101, Georgia		
Study Doctor:<br>Dr. Iza Jikia<br>National Center for Tuberculosis and Lung Diseases<br>50, Maruashvili Str., Tbilisi, 0101, Georgia		
Operator:<br>Malkhaz Tsotskhalashvili<br>National Center for Tuberculosis and Lung Diseases<br>50, Maruashvili Str., Tbilisi, 0101, Georgia		

TABLE OF CONTENTS

1. SUMMARY 7
2. INTRODUCTION AND RATIONALE 9
3. OBJECTIVES 11
4. STUDY DESIGN & TIMELINE 11
5. STUDY POPULATION 12
   5.1 Inclusion criteria 12
   5.2 Exclusion criteria 12
6. INVESTIGATIONAL PRODUCT/TREATMENT 13
   6.1.1 Dose and dosage regimen: 13
   6.1.2 Summary of known and potential risks and benefits: 14
7. OUTCOMES AND OUTCOME MEASUREMENTS 15
8. STUDY PROCEDURES 16
9. SAMPLE SIZE CALCULATION AND OTHER STATISTICS CONSIDERATIONS 19
10. RANDOMIZATION, ALLOCATION AND BLINDING 20
11. ETHICAL CONSIDERATIONS 20
   11.1 Regulation statement 20
   11.2 Recruitment and consent 20
   11.3 Confidentiality 21
   11.4 Withdrawal of individual subjects 21
12. ADMINISTRATIVE ASPECTS, MONITORING AND PUBLICATION 21
   12.1 Handling and storage of data and documents, monitoring 21
   12.2 Monitoring and Quality Assurance 22
   12.3 Amendments 23
   12.4 End of study report 23
   12.5 Public disclosure and publication policy 23
13. REFERENCES 24
14. ANNEX 1 26

LIST OF ABBREVIATIONS AND RELEVANT DEFINITIONS

Adverse Events (AE)

Alanine transaminase (AST)

Aspartate transaminase (ALT)

Case Report Form (CRF)

Confidence Interval (CI)

Controlled Clinical Trials (RCT)

Drug Sensitive Tuberculosis (DS-TB)

Health Quality of Life (HQoL)

Host-Directed Therapies (HDT)

Month (M)

Multi-Resistant to Drugs strains (MDR/XDR-TB)

M. tuberculosis (Mtb)

National Center for Tuberculosis and Lung Diseases (NCTLD)

Non-Steroid AntiInflammatory Drugs (NSAIDS)

Standard of Care treatment (SoC)

US Food and Drug Administration (FDA)

Tuberculosis (TB)

World Health Organization (WHO)

1. SUMMARY

Rationale and aim: Novel approaches to improve TB treatment outcomes (to reduce morbidity, mortality, and the duration of TB treatment) and to treat XDR-TB cases are urgently required. Host-Directed therapies (especially repurposed drugs such as Non-Steroid AntiInflammatory Drugs NSAIDS) could be useful in this context, and therefore its the appropriateness and potential effect of this approach needs to be evaluated in humans. We do propose a pilot study to estimate the potential efficacy and safety of using adjunctive ibuprofen for the treatment of pre-XDR and XDR tuberculosis.

Trial design: This is a prospective, interventional, randomized, clinical trial, open, to estimate the potential efficacy and safety of using Adjunctive ibuprofen for the Treatment of pre-XDR and XDR Tuberculosis.

Methods:

Study population: A total number of 24 patients diagnosed with confirmed Pulmonary pre-XDR or XDR-Tuberculosis and admitted to the National Center for Tuberculosis and Lung Diseases (NCTLD), Georgia, will be asked to participate in this trial.

Intervention: All 24 patients enrolled will receive the appropriate Standard of Care (SoC). A total of n=12 (treatment group) will also receive ibuprofen, orally in a dose of 400mg, daily, during the first 2 months of Standard of Care (SoC) treatment.

Outcomes and Outcomes measurements:

Primary outcome:

Proportion of pilot study participants who show clinical or microbiological benefit of the new regimen relative to controls.

Outcome measurements:

- Percentage of negative sputum culture at M2 following inclusion and Time to stable culture conversion up to M6: (≥ two consecutive cultures negative for *M. tuberculosis*) including all time in follow-up to month six on TB treatment. Will be considered positive if any of them increased by more than a 50%.
- Changes detected by X-ray during follow-up: at baseline and every 3 months (as per routine)

- Final treatment outcomes according to the WHO definitions including all time in follow-up to M6 on TB treatment

Secondary outcomes:

1. Proportion of pilot study participants who tolerate the new regimen. Outcome measurements: incidence of safety-related events during the whole study period: clinical worsening of the disease, no sputum conversion (if AFS-), any worsening concerning vital parameters and routine blood work.
2. Proportion of pilot study participants showing differences in Health Quality of Life (HQoL). Outcome measurements: HQoL measures at M2 and M6 relative to baseline.
3. Proportion of pilot study participants showing differences in Immune Responses at M2 and M6 relative to baseline. Outcome measurements: changes detected in immune responses at M2 and M6.

2. INTRODUCTION AND RATIONALE

Nowadays, Tuberculosis (TB) is still an international public health problem, in spite of existing an effective treatment. Every year, 9 million people get sick and 1 million of them die. Recently, one of the major problems for health systems (in terms of both medical aspect and budget) of all countries are those cases due to Multi-Drug Resistant to Drugs strains (MDR/XDR-TB), which represented up to half a million cases last year(1). The average length of hospital stay and duration of treatment are increasing due to MDR/XDR-TB cases, with an associated increasing burden on National TB Control Programmes. Moreover, these cases have poor treatment outcomes associated and high percentage of death rate(1).

The threat and emergence of multidrug-resistant Tuberculosis (MDR-TB) is a European public health priority. Georgia has a high burden of MDR/XDR-TB. According to WHO country profiles, the estimates of MDR-TB burden in 2013 were 49% of TB cases (considering new plus retreatment cases)(2).

In this context, new approaches to ameliorate the response to TB treatment are needed; especially those aiming to reduce morbidity, mortality and treatment duration, all taking into account the MDR/XDR-TB cases. Moreover, these new options are required to be worldwide available, to be used immediately and for any patient, implying them needed to be cheap(3).

During the last 20 years, a lot of progress has been made in the design and development of new drugs and vaccine candidates, but this is a hard, time and cost-consuming activity with low or none output at in the short-term and doubtful output at in the long-term either (only one out of 10.000 new compounds will arrive to the market and only after 15 years of a rigorous pre-clinical and clinical development)(4).

Meanwhile, physicians deal with clinical TB management based on old premises, most of them strictly clinical and empiric approaches, and the TB drug pipeline stills remains sparse. The recent failure of the most advanced TB vaccine candidate (MVA85A), with a solid 10-years preclinical history has demoralized the scientific community, but has opened new research lines in TB field(4). Within this context, novel approaches to improve TB treatment outcomes (to reduce morbidity, mortality, and the duration of TB treatment), to treat MDR/XDR-TB cases and new therapeutic regimens are urgently required, and the Host-Directed Therapies provide new hope in this sense(5). An equitable Africa-Europe HDT-Network was recently created (http://www.unza-uclms.org/hdt-net) to evaluate a range of HDTs through randomized, controlled clinical trials (RCT), and the UTE is a partner of this network.

Recent basic science suggests new approaches that target human host biology, may improve outcomes (6,7). We plan to contribute to this field of host directed therapies (HDT), for which several drugs have been proposed, based on in vitro testing and/or theoretical concepts, but few have been tested, few are approved by the US Food and Drug Administration (FDA) (8), and several have severe adverse effects, (e.g. thalidomide, etanercept, IFN-γ). Low-risk options have not been explored in clinical trials, despite the urgency of the TB epidemic and high rates of mortality.

Dr Vilaplana et al., have shown the beneficial effect of two NSAIDs, ibuprofen and low - dose aspirin – COX2 and COX1 inhibitors, respectively - in a murine model of active TB (9,10). They showed that, in the absence of TB treatment, both aspirin and ibuprofen markedly and significantly increased survival and decreased both the bacillary load and granuloma in mice treated with ibuprofen. This effect is likely due to NSAIDs reducing pro-inflammatory mediators (TNF-a, IL -17, IL-6 and CXCL5) release rather than a direct toxic effect on mycobacteria. Few pre-clinical published studies have explored the effect of NSAIDs in combination with single antituberculous drugs (11,12), but we have some evidence of benefit on survival when used as an adjunct therapeutic agent to combination TB therapy in mice with TB disease (Vilaplana et al., unpublished data).

The relevant reviews on TB therapies and new approaches to tackle TB using host-directed therapies (HDT) (5,13–18) suggest that HDT might shorten treatment times, reduce lung damage caused, and lower the risk of relapse or reinfection. Moreover, they posit the beneficial effect that drugs with anti-inflammatory effect might have on TB treatment.

We are however, unaware of any studies reporting NSAID co-treatment in humans with pulmonary TB, neither DS or MDR-TB. We found two prior clinical trials of aspirin in patientswith TB meningitis. Firstly, in Cape Town, children were randomised to either: 75mg aspirin daily, 100mg/kg aspirin in three divided doses, or placebo. In 146 children, there were no differences in outcomes - but over 75% of all children had hydrocephalus and a similar proportion had hemiplegia at the time of admission(19). The second, from Lucknow India, randomised 118 adults with TB meningitis to either aspirin 150mg daily or placebo. The aspirin arm had statistically significant reduced mortality and incident stroke three months after randomisation - compared to the placebo arm, without an increase in adverse events(20). Moreover, indomethacin, an NSAID, has been commonly used by physicians to treat TB pleuritis and pericarditis, and its use with TB treatment may improve outcomes (21).

We feel that despite the absence of phase 1 data of Non-Steroid Anti-Inflammatory Drugs (NSAIDS) plus TB drug interactions, that a clinical trial in TB patients is warranted, and we do propose a pilot study to estimate the potential efficacy and safety of using adjunctive

ibuprofen for the treatment of pre-XDR and XDR tuberculosis.

3. OBJECTIVES

Primary Objective: To estimate the potential efficacy of using adjunctive ibuprofen for the Treatment of pre-XDR and XDR Tuberculosis.

Secondary Objectives:

- To explore the safety of adjunctive ibuprofen for the Treatment of pre-XDR and XDR Tuberculosis.
- To evaluate the appropriateness of the secondary measurements for evaluating the effect of adjunctive ibuprofen for the Treatment of pre-XDR and XDR Tuberculosis.

4. STUDY DESIGN & TIMELINE

This is a prospective, interventional, randomized, controlled, open, pilot trial, to estimate the potential efficacy and safety of using adjunctive ibuprofen for the treatment of pre-XDR and XDR-TB.

The diagnosis of pre-XDR and XDR-TB will be established according to WHO criteria (Definitions and reporting framework for tuberculosis – 2013 revision).

Patients identified with pulmonary pre-XDR and XDR-TB will be followed by standard visits and sputum examinations as per clinical routine.

Standard of Care (SoC) TB Treatment will be optimized and drugs selected according to sensitivity profile and per routine.

A total of 24 newly bacteriologically confirmed Pulmonary pre-XDR and XDR-TB TB cases (with and without Extrapulmonary involvement) will be recruited in the National Center for Tuberculosis and Lung Diseases (NCTLD). Potential participants will be offered participation by explaining the information sheet; after screening for in- and exclusion criteria, and appropriate time to consider participation, consenting individuals will receive SoC TB treatment (according to clinical routine and prescriber; n=12) or SoC TB plus ibuprofen (400mg/day/2 months; n=12).

Patients will be followed-up during 6 months for this study. The patients will continue to be followed-up by their physicians fort heir condition as per clinical routine. Initially, a

maximum of 2 years project is expected: setting up: 2 months (M); recruitment: 12M; follow-up of the last patient recruited: 6M; analysis of results and report: 4M. This timeline might vary on the clinical characteristics, treatment and disease course of the patients included, and the budget provisions/restrictions.

Patients will be included during the first year, and followed-up as per clinical routine and according to the medical doctors in charge.

5. STUDY POPULATION

A total number of 24 patients diagnosed with pre-XDR and XDR-TB and admitted to the National Center for Tuberculosis and Lung Diseases (NCTLD), Georgia will be asked to participate in this trial. In this TB hospital around 40 patients with pre-XDR and XDR-TB are treated annually.

5.1 Inclusion criteria

In order to be eligible to participate in this study, a subject must meet all of the following criteria:

1. Females and males aged ≥ 16
2. The patient must provide written informed consent
3. Females of childbearing potential (including females less than 2 years post-menopausal) must have a negative pregnancy test at enrolment and must agree to use highly effective methods of birth control
4. M.tuberculosis (Mtb) detected by culture with available drug susceptibility results for current isolate
5. New confirmed pre-XDR and XDR- TB confirmed by drug susceptibility testing (DST, or any other drug susceptibility test used as per routine in the NCTLD laboratory).

5.2 Exclusion criteria

A potential subject who meets any of the following criteria will be excluded from participation in this study:

1. Inability to provide written informed consent
2. First line drug treatment susceptible Mtb strain
3. Pregnancy/Breastfeeding at inclusion

4. Any of the following laboratory parameters: Aspartate aminotransferase (AST) or alanine aminotransferase (ALT) > 5 x upper limit of normal (ULN); total bilirubine > 2 x ULN; estimated glomerular filtration rate (eGFR) <60ml/hr; Neutrophil count ≤ 500 neutrophils / mm^3; Platelet count < 50,000 cells / mm^3
5. Receiving or anticipated to receive a daily dose of ≥ 10 mg of systemic prednisone or equivalent within the period starting 14 days prior to enrolment, or > 5 doses per week of any NSAID for ≥2 weeks in the month prior to randomisation.
6. History of sensitivity or allergy to ibuprofen.
7. A history suggestive of any of the following in past two years: peptic ulcer disease, gastro-intestinal bleeding, coagulopathy, or renal disease requiring hospitalisation, history of severe cardiovascular disease or symptoms suggestive of severe cardiovascular disease.
8. Co-treatment in the three months prior to randomisation with anticoagulant therapy or immune modulating therapy (cancer treatments, oral or inhaled steroids; etc.).
9. Patients with HIV infection
10. Alcohol use: Drinks more than an average of three units/day over a usual week's consumption.
11. Major co-morbid conditions precluding full evaluation or any other finding which in the opinion of the investigator would compromise the protocol compliance or significantly influence the interpretation of results.

6. INVESTIGATIONAL PRODUCT/TREATMENT

Ibuprofen is a 'repurposed' drug with anti-inflammatory effect and potential anti-TB activity which is cheap, has an excellent safety profile and have been widely used for decades as anti-inflammatory drugs for pain, arthritis (ibuprofen). Ibuprofen has been used since 1961, approved for over-the-counter sale (OTC) without prescription and included in the 18th World Health Organisation (WHO) Essential Medicines List (23).

6.1.1 Dose and dosage regimen:

Ibuprofen used will be as those usually administered as antiinflammatories per clinical routine and provided by the Pharmacy Department of the NCTLD.

Ibuprofen will be administered orally in a dose of 400mg, daily, during the first 2 months of SoC treatment.

The follow-up period will start on the day of inclusion and will continue with visits as prescribed per clinical routine.

6.1.2 Summary of known and potential risks and benefits:

Ibuprofen has been used since 1961, respectively, approved for over-the-counter sale (OTC) without prescription and included in the 18th World Health Organisation (WHO) Essential Medicines List (23).

NSAIDs are not innocuous. The most frequent adverse events (AE) are gastrointestinal (GI) damage or bleeding complications (both incident or exsacerbation of existing conditions). A systematic review of 20 studies concluded that the incidence of GI bleeding events with use of OTC ibuprofen was low, the frequency of a GI- related hospitalization being <0.2%, and the incidence rates among those using OTC -comparable doses ranged from 0 to 3.19 per 1000 patient-years review (24). Even short-term use (less than 14 days) results in dose- dependent gastric lesions for all NSAIDs; while long- term (3 months or more) endoscopy studies in patients show ulcer rates from 15%-35% with the various NSAIDs (25). NSAID treatment has an increased risk of thrombotic events, myocardial infarction, and stroke, which can be fatal, especially in patients with pre-exisitng cardiovascular disease or risk factors (26)(27). Borderline elevations of one or more liver tests may occur in up to 15% of patients taking NSAIDs but elevations of aspartate transaminase (ALT) or alanine transaminase (AST) (approximately three or more times the upper limit of normal) occur in 1% patients, especially at doses of 2,400-3,200mg of ibuprofen, but these resolve rapidly with discontinuation (28). Water and electrolyte imbalances have been reported. Long-term administration of NSAIDs has resulted in renal papillary necrosis and other renal injury, especially in those with renal disease. NSAIDs can cause serious rashes: exfoliative dermatitis, Stevens-Johnson syndrome, and toxic epidermal necrolysis, which can be fatal. The drug should be discontinued at the first appearance of skin rash or any other sign of hypersensitivity. Asthma and other hypersensitivity responses can occur with NSAIDs and anaemia is sometimes seen with NSAID treatment (26). Most AE's are dose-related and the dose of ibuprofen used in our study will be relatively low (400mg) but we will implement a process of symptom and objective screening for adverse events.

The present CT will provide direct information on safety of the interventions proposed for XDR-TB, and on feasibility of conducting a full-scale trial involving M/XDR-TB patients; and even proof of concept for a trial in Drug-Sensitive TB patients. Moreover, If successful, the experimental drug can show potential to improve clinical management and treatment outcomes of XDR-patients. The experimental drug also has the potential to prevent severe complications and possibly lower mortality in XDR-TB cases by decreasing the lung damage due to the disease.

7. OUTCOMES AND OUTCOME MEASUREMENTS

Primary outcome:

Proportion of pilot study participants who show clinical or microbiological benefit of the new regimen relative to controls.

Outcome measurements:

- Percentage of negative sputum culture at M2 following inclusion and Time to stable culture conversion up to M6: (≥ two consecutive cultures negative for *M. tuberculosis*) including all time in follow-up to month six on TB treatment. Will be considered positive if any of them increased by more than a 50%.
- Changes detected by X-ray during follow-up: at baseline and every 3 months (as per routine)
- Final treatment outcomes according to the WHO definitions including all time in follow-up to M6 on TB treatment

Secondary outcomes:

- Proportion of pilot study participants who tolerate the new regimen. Outcome measurements: incidence of safety-related events during the whole study period: clinical worsening of the disease, no sputum conversion (if AFS-), any worsening concerning vital parameters and routine blood work.

- Proportion of pilot study participants showing differences in Health Quality of Life (HQoL). Outcome measurements: HQoL measures at M2 and M6 relative to baseline.
- Proportion of pilot study participants showing differences in Immune Responses at M2 and M6 relative to baseline. Outcome measurements: changes detected in immune responses at M2 and M6.

8. STUDY PROCEDURES

Study participants with pre-XDR and XDR-TB will be identified using classical culture and drug susceptibility assays. Treatment will be tailored using either optimized treatment for XDR-TB based on molecular drug sensitivity testing (Line-Probe assays), or classical phenotypical Drug Susceptibility Testing. Drug dosing will be optimized, based on therapeutic drug monitoring when clinically indicated and available.

The attending physician will propose to a potential study participant that the study doctor or trial site coordinator comes to discuss inclusion in the trial. The patient will be informed by the study doctor and given enough time to consider his participation. An independent expert will be reachable for any questions. The clinical history will be taken and inclusion and exclusion criteria will be assessed. After exclusion of patients that cannot participate in this study and informed consent of participating patients, these consenting patients will be enrolled.

The enrolled patients will be treated with the best-personalized tailored second line regimens on the basis of the latest Drug susceptibility testing (DST) of *M.tuberculosis* isolates in accordance with national pre-XDR and XDR-TB guidelines.

After chemotherapy initiation patients are monitored routinely in accordance with national pre-XDR and XDR-TB guidelines.

<u>Patients' monitoring and evaluation:</u>

Patient management will be left to the discretion of the NCTLD team.

The panel of doctors from the investigator team will treat and follow up the patients as per clinical routine. They will be responsible for collecting the data requested in the appropriate forms (which will be created ad-hoc).

Subjects will be followed-up for 6M after recruitment and any impact of the interventions on safety, lung damage, Health Quality of Life (HQoL) and TB outcomes (according to WHO definitions' criteria).

Clinical examination will be done at treatment initiation then as recommended per clinical routine in the NCTL, or as required.

Adverse events (clinical, laboratory and radiological) will be recorded during the whole time the patient will be included, and are defined as:

- worsening and/or exacerbation of TB
- conversion from smear negative to smear positive
- conversion from culture negative to culture positive
- worsening in chest X-ray
- any alteration of blood tests (defined by reference values of the lab methods used at the NCTLD)
- any event worsening health status of the patients.

In case the physician in charge of a patient in the ibuprofen-group decides to suspend ibuprofen for any medical reason or presence of adverse event attributable to it, he/she will record in the crf the date of suspension, will notify the study coordinator and the PI, and will continue the follow-up (including the samples collection) as per protocol.

Sputum conversion monitoring:

Negative sputum culture at M2 following inclusion will be used to evaluate the efficacy of the interventions, as has been identified as a predictor to end-of-treament outcome and relapse and proposed to inform the required duration of treatment of new TB regimens (29). As negative sputum culture at 6 months has been described as marker of end-of-treatment outcome in patients with MDR/XDR tuberculosis, with stronger association with treatment success than 2 month conversion status (30) time to stable culture conversion has been listed as secondary outcome.

Microbiology data will be collected from the routine data provided by the correspondent Microbiology Department.

Radiology monitoring:

Chest X-rays will be performed as per routine: at start of chemotherapy and at every 3 months.

Blood tests monitoring:

Laboratory analysis (biochemical and haemato analysis) will be done as per clinical routine (per month). They will include:

• Absolute and differential blood counts (neutrophiles, monocytes, lymphocytes counts, etc)
• platelets,
• hemoglobin (Hb),
• erythrocytes sedimentation rate (ESR),
• total protein,
• albumin,
• total globulins,
• glucose,
• urea & electrolytes,
• creatinine,
• total (direct and indirect) bilirubin,
• ALT, AST, alkaline phosphatase

Samples for the analysis of the Immune Responses will be collected at baseline, at M2 and M6. A total volume of 18.5mL of pheripheral whole blood will be collected in RNA PaxGene tube (2.5mL) plus 2 CPT tubes (8mL each). Urine samples will also be collected. Samples will be stored frozen until its shipment and process. Dr Vilaplana (Unitat de Tuberculosi Experimental (UTE)) will be in charge of the evaluation of the immune responses with the collaboration of Pr. Helen Fletcher (LSHTM). Initially, the plan is to investigate pro and anti-inflammatory immune responses using RNA collected in PaxGene tubes and plasma, and PBMC will be used to investigate mycobacterial antigen specific cellular immunity. Other analysis might be done with the samples collected depending on the appropriateness according to Dr Vilaplana and Pr Fletcher, and to funding possibilities.

Impact on Health Quality of Life (HQoL) will be measured at baseline, at M2 and M6 with the SGUL Questionnaire (for respiratory diseases) and two questionnaires to evaluate socio-psychological factors: the Kessler 10+ and the questionnaire developed by Vilaplana et al (BCN-Q).

Interim (sputum conversion, chest X-ray improvement) and final (cure, treatment completed, treatment failed, lost to follow up, death) treatment outcomes are to be recorded.

The following chart (Table 1) summarizes the planned visits and study procedures:

Pilot Study of feasibility and methodology to use Adjunctive Ibuprofen for the Treatment of XDR Tuberculosis (**NSAIDS-XDR-TB)**							
	Baseline	Follow-up					
Month	0	1	2	3	4	5	6
Visit	1	2	3	4	5	6	7
Informed consent	x						
Inclusion	x						
Clinical examination	x	x	x	x	x	x	x
Sputum culture	x	x	x	x	x	x	x
Rx assay	x			x			x
Blood tests	x	x	x	x	x	x	x
Adverse events recording	x	x	x	x	x	x	x
Health Quality of Life Questionnaires	x		x				x
Sample collection (whole blood in 1 RNA PaxGene Tubes, whole blood in 2 PCT tubes and urine) for Evaluation of the Immune responses	x		x				x
Interim treatment outcomes assessment				x			x
Final outcomes assessment				x			x

9. SAMPLE SIZE CALCULATION AND OTHER STATISTICS CONSIDERATIONS

This is a pilot, thus to see the magnitude and direction of possible responses, and not powered to show smaller differences. We plan to use the data generated to design a larger trial.

The total n of 12 per group has been decided based on feasibility, gains in the precision about the mean and variance, and regulatory considerations according to literature on pilot studies (22). The results obtained will be expressed as percentages and the associated 95%

Confidence Interval (CI). Descriptive statistics for patient demographic data, clinical and radiographic parameters, comorbidities, co-infection, full laboratory data including blood indices, blood chemistries, as well as all microbiological data will be performed. Results of ibuprofen-treated group will be compared to those obtained in the group of patients treated with SoC alone, which will be considered controls. Patients offered to enrol the study and who preferred not to will might also be considered as controls.

10. RANDOMIZATION, ALLOCATION AND BLINDING

Participants will be randomly allocated to one of the experimental groups at an allocation ratio of 1:1, according to an allocation sequence generated randomly by the sponsor, in order to each participant to have equal probability of being allocated to the intervention group or the control group. The random allocation sequence will be generated using a random computer program. The random allocation sequence will remin concealed from those enrolling patients into the study; the individual recruiting the patient will contact a central methods center by phone or secure computer after the patient is enrolled.

This pilot trial will be open, no blinding will apply.

11. ETHICAL CONSIDERATIONS

11.1 Regulation statement

The clinical trial will be conducted according to the principles of the Declaration of Helsinki (October 2008), the Medical Research Involving Human Subjects Act and the ICH guidelines. The protocol of the study will be submitted to the appropriate Ethics Committee.

In all cases an informed consent form (attached as Annex 1) will be filled in and signed by each patient to be included to the present project prior to start.

11.2 Recruitment and consent

Potential participants will be informed by their reference medical doctor (pthisiologist/pulmonologist) in the NCTLD, about the purpose of the study, the investigational procedures and the potential risks (side effects) and benefits. Potential participants will have 48 hours to consider the decision to participate in this study.
Incentives

Participants are not going to receive any economic reward or any other kind of reward for participating.

11.3 Confidentiality

All samples collected will be recorded and labeled according to a specific code for the present project (NSAIDS-XDR) plus the number correspondent to the patient's number).

For more specifics, please check Section 10 (Handling and storage of data).

11.4 Withdrawal of individual subjects

Subjects can leave the study at any time for any reason if they wish to do so without any consequences. The investigator can decide to withdraw a subject from the study for urgent medical reasons or if there is any reason to think that completion of protocol or data validity is compromised. Study participants deciding to leave the study do not necessarily have to give a specific reason to stop, but the study team members will invite the participant leaving the study to explain their decision to do so. Usual standard care is offered to the patients who wish to stop the participation in this study. Data from withdrawn patients will be analysed. Withdrawn patients will be replaced unless the reason for withdrawal is the presence of advers effects related to the ibuprofen treatment and that this compromises the continuation of the study. Replacement subjects will receive the same treatment as the withdrawn patients they are replacing, and all their paperwork and samples will be labelled with the same code but adding and R (R for replacing). Exemple: if subject NSAIDS-XDR-003 was withdrawn, subject recruited as number 25 will replace it with the code NSAIDS-XDR-003R, and will be allocated to the same group as NSAIDS-XDR-003, and therefore will receive the same medication as NSAIDS-XDR-003.

12. ADMINISTRATIVE ASPECTS, MONITORING AND PUBLICATION

12.1 Handling and storage of data and documents, monitoring

Each patient will be given a sequential number coded as NSAIDS-XDR XXX (XXX meaning the correspondent number of patient).

For each patient, a non-anonymized form created ad-hoc for this project will correlate him/her with his/her projects' code. This form will be to be kept together with the Medical Record of each patient as the Hospital Records copy. A spreadsheet file will be filled-in with the following data of each patient included: name, the Hospital Record Number and the correspondent Project's code (NSAIDS-XDR XXX). Only persons involved in the study and

after prior training will receive access to the data. A Case Report Form (CRF) will be created ad-hoc and filled-in for each patient included with all the data required: Project's code (NSAIDS-XDR XXX), clinical characteristics and monitoring data. In order to comply with the patients' rights of confidentiality, this form will be anonymized, without the name and surnames of the patient and where only the project's code will be recorded, together with all the clinical data associated to the specific case.

In order to comply with the ethics requirements of samples' collections, both documents will be kept as 2 different documents and stored in 2 different computers.

The data, collected in a database will be kept for the period of 15 years. Human material will be kept for 5 years and, if considered appropriate, data and samples could be used for different studies promoted by the sponsor, as indicated in the patient consent form. After the period of 5 years samples will be destroyed.

12.2 Monitoring and Quality Assurance

Data from the study will be collected in the adhoc CRF.

During the study the Coordinating Investigator will visit the investigational sites to confirm that the facilities remain acceptable, that the investigational team is adhering to the protocol and that data are being accurately recorded in the CRFs. Source data verification (a comparison of the data in the CRF with the subjects laboratory test results and other source documents) will also be performed.

Authorized representatives of the regulatory authority may visit the center to perform inspections, including source data verification.

Clean File for the final database will be declared when all data have been entered and a quality check on a sample of the data has been performed. The database will be locked after Clean File has been declared and data extracted for statistical analysis. Study committee meetings will be held as needed prior to or during the study. The medical, nursing and other staff involved in the study will receive proper education/information on how to conduct the study according to the protocol.

12.3 Amendments

A 'substantial amendment' is defined as an amendment to the terms of the Ethics Committe application, or to the protocol or any other supporting documentation, that is likely to affect to a significant degree:

- the safety or physical or mental integrity of the subjects of the trial;
- the scientific value of the trial;
- the conduct or management of the trial; or
- the quality or safety of any intervention used in the trial.

All substantial amendments will be notified to the Ethics Committee and to the competent authority. Non-substantial amendments will not be notified to the accredited Ethics Committe and the competent authority, but will be recorded and filed by the sponsor.

12.4 End of study report

The coordinating investigator will notify the accredited Ethics Committe and the competent authority of the end of the study within a period of 90 days. The end of the study is defined as the last patient's last visit.

In case the study is ended prematurely, the sponsor will notify the accredited Ethics Committe and the competent authority within 15 days, including the reasons for the premature termination.

Within one year after the end of the study, the investigator/sponsor will submit a final study report with the results of the study, including any publications/abstracts of the study, to the accredited Ethics Committe and the Competent Authority.

12.5 Public disclosure and publication policy

A final report/communication of results is expected from the coordinating investigator in collaboration with the site PI and the investigator team for the end of the project, but other dissemination activities to share the results will be carried out if considered appropriate by the coordinating investigator without any restrictions to its content.

13.REFERENCES

1. Who. Global tuberculosis report 2014 (WHO/HTM/TB/2014.08). 2014;
2. World Health Organization. WHO Tuberculosis country profiles [Internet]. 2015 [cited 2015 Sep 30]. Available from: http://www.who.int/tb/country/data/profiles/en/
3. Zumla A, Abubakar I, Raviglione M, Hoelscher M, Ditiu L, McHugh TD, et al. Drug-Resistant tuberculosis-current dilemmas, unanswered questions, challenges, and priority needs. J Infect Dis. 2012;205(Suppl 2).
4. Zumla AI, Gillespie SH, Hoelscher M, Philips PPJ, Cole ST, Abubakar I, et al. New antituberculosis drugs, regimens, and adjunct therapies: Needs, advances, and future prospects. The Lancet Infectious Diseases. 2014.
5. Schito M, Migliori GB, Fletcher HA, Mcnerney R, Centis R, D 'ambrosio L, et al. Perspectives on Advances in Tuberculosis Diagnostics, Drugs, and Vaccines.
6. Zumla A, Chakaya J, Hoelscher M, Ntoumi F, Rustomjee R, Vilaplana C, et al. Towards host-directed therapies for tuberculosis. Nat Rev Drug Discov. 2015;
7. Zumla A, Rao M, Parida SK, Keshavjee S, Cassell G, Wallis R, et al. Inflammation and tuberculosis: host-directed therapies. J Intern Med. 2014 Apr;
8. Kaufmann SHE, Lange C, Rao M, Balaji KN, Lotze M, Schito M, et al. Progress in tuberculosis vaccine development and host-directed therapies-a state of the art review. Lancet Respir Med [Internet]. Elsevier Ltd; 2014;2(4):301–20. Available from: http://dx.doi.org/10.1016/S2213-2600(14)70033-5
9. Vilaplana C, Marzo E, Tapia G, Diaz J, Garcia V, Cardona PJ. Ibuprofen therapy resulted in significantly decreased tissue bacillary loads and increased survival in a new murine experimental model of active tuberculosis. J Infect Dis. 2013;208:199–202.
10. Marzo E, Vilaplana C, Tapia G, Diaz J, Garcia V, Cardona P-J. Damaging role of neutrophilic infiltration in a mouse model of progressive tuberculosis. Tuberculosis (Edinb). 2014 Jan;94(1):55–64.
11. Tobin DM, Roca FJ, Oh SF, Mcfarland R, Vickery TW, Ray JP, et al. Host genotype-specific therapies can optimize the inflammatory response to mycobacterial infections. February. 2012;3(1483):434–44612.
12. Byrne ST, Denkin SM, Zhang Y. Aspirin and ibuprofen enhance pyrazinamide treatment of murine tuberculosis. J Antimicrob Chemother. 2007;59(December 2006):313–6.
13. Zumla A, Chakaya J, Centis R, D'Ambrosio L, Mwaba P, Bates M, et al. Tuberculosis treatment and management—an update on treatment regimens, trials, new drugs,

and adjunct therapies. Lancet Respir Med [Internet]. 2015;3(March):220–34. Available from: http://linkinghub.elsevier.com/retrieve/pii/S2213260015000636

14. Zumla A, Maeurer M, Chakaya J, Hoelscher M, Ntoumi F, Rustomjee R, et al. Host-Directed Therapies for tackling Multi-Drug Resistant TB – learning from the Pasteur-Bechamp debates , and the Host-Directed Therapies Network (HDT-NET). 2015; Available from: http://cid.oxfordjournals.org/
15. Wallis RS, Hafner R. Advancing host-directed therapy for tuberculosis. Nat Publ Gr. 2015;15.
16. Hawn TR, Matheson AI, Maley SN, Vandal O. Host-Directed Therapeutics for Tuberculosis: Can We Harness the Host?
17. Ivanyi J, Zumla A. Nonsteroidal Antiinflammatory Drugs for Adjunctive Tuberculosis Treatment.
18. Kaufmann SHE, Lange C, Rao M, Balaji KN, Lotze M, Schito M, et al. Review Progress in tuberculosis vaccine development and host-directed therapies—a state of the art review. 2013; Available from: www.thelancet.com/respiratory
19. Schoeman JF, Janse van Rensburg A, Laubscher JA, Springer P. The role of aspirin in childhood tuberculous meningitis. J Child Neurol. 2011;
20. Misra UK, Kalita J, Nair PP. Role of aspirin in tuberculous meningitis: A randomized open label placebo controlled trial. J Neurol Sci. 2010;293:12–7.
21. Litvin LM, Novosad MK. [The treatment of patients with destructive pulmonary tuberculosis with indomethacin and tuberculostatic preparations]. Vrach Delo. 1990 May;(5):76–8.
22. Julious SA. Sample size of 12 per group rule of thumb for a pilot study. Pharm Stat. 2005;4:287–91.
23. WHO. 18th WHO Model List of Essential Medicines. 2013.
24. Michels SL, Collins J, Reynolds MW, Abramsky S, Paredes-Diaz A, McCarberg B. Over-the-counter ibuprofen and risk of gastrointestinal bleeding complications: a systematic literature review. Curr Med Res Opin. 2012 Jan;28(1):89–99.
25. Bjarnason I. Gastrointestinal safety of NSAIDs and over-the-counter analgesics. Int J Clin Pract Suppl. 2013 Jan;(178):37–42.
26. Food and Drug Administration. Medication Guide for Nonsteroidal Anti-inflammatory Drugs (NSAIDs).
27. US Food and Drug administration. FDA Drug Safety Communication: FDA strengthens warning that non-aspirin nonsteroidal anti-inflammatory drugs (NSAIDs)

can cause heart attacks or strokes [Internet]. [cited 2015 Dec 29]. Available from: http://www.fda.gov/Drugs/DrugSafety/ucm451800.htm

28. National Institute of Health. LiverTox.nih.gov [2015].

29. Wallis RS, Peppard T, Hermann D. Month 2 Culture Status and Treatment Duration as Predictors of Recurrence in Pulmonary Tuberculosis: Model Validation and Update.

30. Kurbatova E V, Cegielski JP, Dalton T, Ershova J, Gammino VM, Heilig CM, et al. Sputum culture conversion as a prognostic marker for end-of-treatment outcome in patients with multidrug-resistant tuberculosis: a secondary analysis of data from two observational cohort studies. Lancet Respir [Internet]. 2015;3:201–9. Available from: http://dx.doi.org/10.1016/

ANNEX 1. INFORMED CONSENT FORM

INFORMED CONSENT SHEET

This data sheet may contain terms that you don´t understand. Do not hesitate to ask your doctor or the attending personal any questions that you may have.

Title: "Pilot Study to estimate the potential efficacy and safety of using Adjunctive Ibuprofen for the Treatment of pre-XDR and XDR Tuberculosis."

We are writing to inform you about a research study we would like you to join. The study was approved by the Clinical Research Ethics Committee of the National Center for Tuberculosis and Lung Diseases (NCTLD) and is conducted according all the current European Laws on Ethical Research.

Our intention is just to provide you with the correct and necessary information to help you consider and decide if you want to participate or not. For this, we are asking you to read this information carefully. The study's doctor will clarify any doubts you may have after reading the explanation.

The aim of this document is to offer you information in order you can decide if you want to hand us your biological samples exclusively with research purposes, in the way we will explain you below. To conduct the present Project we do expect to enrol 24 patients suffering the same condition as you have.

Why is this study taking place?

Tuberculosis is still an important problem around the world. Every year 100 million people acquire this infection. Its prevention is currently very difficult, since this is a disease caused by an airborne bacterium (*Mycobacterium tuberculosis*). Of all people getting infected, only some will develop the disease and, to this date, we still don´t know why some people get sick and why other's don´t. Georgia has a high burden of people ill from tuberculosis (TB), half of them being due to bacilli resistant to common therapies (MDR/XDR-TB), and for this reason its physicians and surgeons are experts on TB management. The scientific community continuously tries to develop new and better

therapies for these patients suffering from MDR/XDR-TB to ameliorate their health of life, reduce the complications and consequences of their disease, and to shorten the treatment duration. One of the latest approaches is the belief of drugs with an anti-inflammatory effect could benefit MDR/XDR-TB patients helping them to cope with the disease.

A research team from Barcelona (Catalonia, Spain) leaded by Dr Vilaplana and in collaboration with the team of the NCTLD headed by Dr.Vashakidze want to study if patients suffering from pre-XDR and XDR-TB could benefit of adding a non-steroid anti-inflammatory drug (NSAID) to their treatment for a period time (the first 2 months).

What will you have to do in this study?

You have been approached because you will be treated for your condition.

If you do accept to participate, we will give you the routinely prescribed therapy regimen and if you are part of the intervention group, you will also receive a NSAID (ibuprofen) during the first 2 months. If you are part of the control group, you will only receive the routinely prescribed therapy regimen. Neither you or your doctor will decide in which group you will be.

This will not modify any procedure from the prescribed routine designed by your doctor. This will not represent any added procedure to your clinical management plan but extra sample collection (blood and urine) and the addition of another drug if you are part of the intervention group.

The study consists in having 2 groups of patients suffering of XDR-TB which will both receive the best therapy regimen according tot their condition: while one group (control) will receive this, the other group (intervention group) will also receive a non-steroid anti-inflammatory drug during the first 2 months. The samples will be collected in the NCTLD and sent to the Experimental Tuberculosis Unit lab to be kept and further processed by the investigator team (person in charge: Dr. Cristina Vilaplana); they being handled according to the Biomedical Research Law 14/2007. They will be labeled with the study's research code (a numeric one that does not identify you directly), and they will only be used for the study's purposes. Once the study will be finished, they will be kept during 10 years, and might be used for future research related to the disease by the investigator team, previously approved by an Ethics Committee.

The results will be introduced in a database and will be used for scientific purposes, and the data will always be handled by specialized personnel.

Which are the benefits, risks and alternatives to participate in this study?

The participation in this study is voluntary, and if you are not willing to collaborate in it, this will not represent any harm for you.

Your participation in this study won´t change the treatment you will receive for your condition.

If you do not participate in this study, you will still receive the more appropriate treatment for your condition, as if you would during the participation. Your participation will not modify any procedure from the prescribed routine designed by your physician. This will not represent any added procedure to your clinical management plan but an extra blood and urine sample collection.

If you are part of the control group, no intervention will be given to you besides the routine schedule to manage your condition, thus neither potential side effects nor extra-benefit are expected from this study.

If you are part of the intervention group, you will receive a non-steroid anti-inflammatory drug once a day during the first 2 months of treatment, thus you could experience some effects of the drug. NSAIDs are not innocuous, and their most frequent adverse events are gastrointestinal (GI) damage or bleeding complications. Other adverse events are increased risk of thrombotic events, myocardial infarction, and stroke; renal injury, elevation of hepatic transaminases, rashes, anaemia and hipersensitivity. However, ibuprofen has been used since 1961, respectively, approved for over-the-counter sale (OTC) without prescription and considered by the World Health Organisation as an essential medicine. Most adverse effects are dose-related, this meaning they happen more frequently when higher doses of NSAIDs are administered (>1200mg/daily), and the dose of ibuprofen you are going to receive if being part of the intervention group will be relatively low (400mg/daily). As you will be closely monitored by your physician, any adverse effect which might be due to the ibuprofen will be rapidly detected, and the drug suspended if your doctor considers it safer for you. Ibuprofen has also the potential to improve your clinical management and your final outcome, by preventing or decreasing the severity of the complications of your condition by decreasing the damage in your lung due to the disease.

The Research Foundation Germans Trias I Pujol (IGTP), a Research Institute from the

European Union located in Badalona, Catalonia (Spain), is financing this study.

Your participation in this study will have no cost for you and you are not going to receive any economic reward or any other kind of reward for participating.

In any case, your participation in this study may be of great help to understand and manage your disease and will contribute to provide valuable information on how to help in a short future other patients suffering the same condition as you .

Do you have to remain in the study?

Your participation is voluntary. If you do not want to participate in the study, tell the study's doctor. At any time you can decide not to continue without giving any reason for it. If you don´t want to participate in the study or if you leave the study, you will be treated as usual.

If you decide to stop participating in this study, please contact the study's physician.

All data and samples collected before your withdrawal from the study will be used for the purposes of the study.

Even if you are not interested in participating in the study anymore, your health information is very important for this study. We will ask you to let us gather information about your health till the completion of the study. For this reason, and with your permission, we would be in contact with you or, if it was not possible, with your relatives, friends or your physician. We will ask for the relevant contact information in your case.

What happens with your personal data?

The data will be coded to preserve your identity, according to the Organic Spanish Law 15/1999 from December 13th, devoted to personal data protection. According to this law you have the right to access, change, oppose and cancel your data. To do any of these you must contact your study's physician. You can rest assured that your clinical history information will be strictly confidential and your identity will be kept anonymous.

When you will accept to participate, a code will be generated and be used to match your samples with your clinical data without need to use your personal data. Only the people responsible of this study (principal investigator and investigator team) will have

access to these data if necessary. Your identity will not be revealed to anybody except when required by medical emergencies or legal requests.

Your identity will remain confidential even if some results of the study are published.

This research study will not generate any data directly applicable to you regarding your condition, as the value of the results obtained is expected at long-term.

Questions and Answers:

If you have any question or doubt related to this study please contact your physician; the physician in charge of the study, Dr. Vashakidze at the following address: 50 Maruashvilii str. 0101 Tbilisi, Georgia Tel: 995-599-176898, email: sergovashakidze@yahoo.com; or the Head of the Ethics Committee Dr. Leila Goginashvili at the following address: 50 Maruashvilii str. 0101 Tbilisi, Georgia Tel: 995-032-2910251, email: leila.goginashvili@tbgeo.ge.

INFORMED CONSENT FORM for the study entitled "Pilot Study to estimate the potential efficacy and safety of using Adjunctive Ibuprofen for the Treatment of pre-XDR and **XDR Tuberculosis"**

Patient's code number: ____________________

I ____ (complete name)

…have read the information sheet that was given to me

…have been able to make inquiries about the study

…have received enough information about the study

…understand that my participation is voluntary

…understand that I can withdraw myself from the study:

1. Whenever I want
2. Without giving any explanations
3. Without it affecting my medical care

For which I hereby consent to participate in the study and to give access to my data and samples related to the study under the conditions mentioned in the information sheet.

Date and place: __________________

Participant's Signature: ______________________

Date and place: __________________

Researcher's Signature: ______________________

This document will be signed in duplicate; one copy goes to the researcher and another one to the participant.

STATISTICAL ANALYSIS PLAN

“Pilot Study to estimate the potential efficacy and safety of using Adjunctive Ibuprofen for the Treatment of pre-XDR and XDR Tuberculosis”.

Code: NSAIDS-XDR-TB

Sample size was decided based on feasibility, gains in the precision about the mean and variance, and regulatory considerations according to literature on pilot studies (1,2). Results of ibuprofen-treated group will be compared to those obtained in the group of patients treated with SoC alone, which will be considered controls. Patients offered to enrol the study and who preferred not to will might also be considered as controls.

This is a pilot, thus to see the magnitude and direction of possible responses, and not powered to show smaller differences. We plan to use the data generated to design a larger trial. All hypothesis tests in this pilot will be prespecified as exploratory.

Both primary and secondary analyses followed an intention-to-treat (ITT) approach including all randomized participants with available data.

Descriptive statistics for patient demographic data, clinical and radiographic parameters, comorbidities, co-infection, full laboratory data including blood indices, blood chemistries, as well as all microbiological data will be performed. Continuous variables will be reported as medians and interquartile ranges (IQR) and compared with the Mann–Whitney U test; categorical data presented as counts and percentages. Absolute risk differences with 95% CIs will be calculated using the Newcombe method. For radiological outcomes ratios at month 3 (M3/BL) and month 6 (M6/BL) will be calculated, considering ratios <1 as favourable. Health-related quality-of-life good responders will be defined as normal range values (SGRQ ≤7 and K10 <20) at both months 2 and 6. Immune response changes will be expressed as ratios to baseline and compared with the Mann–Whitney U test.

REFERENCES

1. Julious SA. Sample size of 12 per group rule of thumb for a pilot study. Pharmaceutical Statistics 2005; 4: 287–91.

2. Whitehead AL, Julious SA, Cooper CL, Campbell MJ. Estimating the sample size for a pilot randomised trial to minimise the overall trial sample size for the external pilot and main trial for a continuous outcome variable. Statistical Methods in Medical Research 2016; 25. DOI:10.1177/0962280215588241.
