# Supplementary material for: Adjunctive ibuprofen in pre-extensively drug-resistant and extensively drug-resistant tuberculosis: a phase IIA open-label pilot clinical trial
Source: Nat Commun. 2026 Jul 1;17:8185. doi: 10.1038/s41467-026-75148-9 (PMC13462894; doi:10.1038/s41467-026-75148-9)
Supplement: Supplementary file 2 [file 41467_2026_75148_MOESM2_ESM.pdf]

nature portfolio

Corresponding author(s): Dr Cristina Vilaplana

Last updated by author(s): June 1st, 2026

Reporting Summary

Nature Portfolio wishes to improve the reproducibility of the work that we publish. This form provides structure for consistency and transparency in reporting. For further information on Nature Portfolio policies, see our Editorial Policies and the Editorial Policy Checklist.

Statistics

For all statistical analyses, confirm that the following items are present in the figure legend, table legend, main text, or Methods section.

n/a	Confirmed	
☐	☒	The exact sample size (*n*) for each experimental group/condition, given as a discrete number and unit of measurement
☒	☐	A statement on whether measurements were taken from distinct samples or whether the same sample was measured repeatedly
☐	☒	The statistical test(s) used AND whether they are one- or two-sided<br>*Only common tests should be described solely by name; describe more complex techniques in the Methods section.*
☒	☐	A description of all covariates tested
☐	☒	A description of any assumptions or corrections, such as tests of normality and adjustment for multiple comparisons
☐	☒	A full description of the statistical parameters including central tendency (e.g. means) or other basic estimates (e.g. regression coefficient) AND variation (e.g. standard deviation) or associated estimates of uncertainty (e.g. confidence intervals)
☐	☒	For null hypothesis testing, the test statistic (e.g. *F*, *t*, *r*) with confidence intervals, effect sizes, degrees of freedom and *P* value noted<br>*Give P values as exact values whenever suitable.*
☒	☐	For Bayesian analysis, information on the choice of priors and Markov chain Monte Carlo settings
☒	☐	For hierarchical and complex designs, identification of the appropriate level for tests and full reporting of outcomes
☒	☐	Estimates of effect sizes (e.g. Cohen's *d*, Pearson's *r*), indicating how they were calculated

Our web collection on statistics for biologists contains articles on many of the points above.

Software and code

Policy information about availability of computer code

Data collection	Clinical data were collected and managed using REDCap (Research Electronic Data Capture) electronic data capture tools hosted at the University of the Witwatersrand, South Africa (REDCap version 12.2.11)
Data analysis	Statistical analyses were performed using GraphPad Prism (version 10.0), R studio v.4.3.1 and Python v3.11.6.

For manuscripts utilizing custom algorithms or software that are central to the research but not yet described in published literature, software must be made available to editors and reviewers. We strongly encourage code deposition in a community repository (e.g. GitHub). See the Nature Portfolio guidelines for submitting code & software for further information.

Data

Policy information about availability of data

All manuscripts must include a data availability statement. This statement should provide the following information, where applicable:

- Accession codes, unique identifiers, or web links for publicly available datasets
- A description of any restrictions on data availability
- For clinical datasets or third party data, please ensure that the statement adheres to our policy

Protocol, including Patient information sheet, Consent form, and Statistical Analysis Plan, is available at https://doi.org/10.5281/zenodo.20342393. RNA-seq data have been deposited in the NCBI Gene Expression Omnibus (GEO) database: GSE298762 [https://www.ncbi.nlm.nih.gov/geo/query/acc.cgi?acc=GSE298762]. Metabolomics data have been deposited in the EMBL-EBI MetaboLights database: MTBLS13376 [https://www.ebi.ac.uk/metabolights/MTBLS13376]. Individual-

level patient data cannot be made available due to patient confidentiality requirements, the conditions of ethics approval granted by the Ethics Review Committee of the National Center for Tuberculosis and Lung Diseases (NCTLD), Georgia, the terms of written informed consent provided by study participants, and applicable data protection legislation. Source data are provided with this paper.

Research involving human participants, their data, or biological material

Policy information about studies with human participants or human data. See also policy information about sex, gender (identity/presentation), and sexual orientation and race, ethnicity and racism.

Reporting on sex and gender	Both male and female participants were eligible for inclusion.Sex was recorded as a binary variable (male/female) based on self-report at enrolment. Sex was not prespecified as an analytical variable in the study protocol given the small sample size and exploratory nature of the pilot; no sex-stratified analyses were carried out. Of the 28 participants enrolled, 75% were male.
Reporting on race, ethnicity, or other socially relevant groupings	Race and ethnicity were not collected as variables in this study. The trial was conducted at the National Center for Tuberculosis and Lung Diseases in Georgia and reflects the population served by this national referral centre for drug-resistant tuberculosis. No analyses were performed based on race, ethnicity, or other socially defined groupings.
Population characteristics	Eligible participants were males and females aged 16 years or older with bacteriologically confirmed pulmonary pre-XDR or XDR tuberculosis, with or without extrapulmonary involvement, according to WHO definitions. The median age was 44 years, and 75% of participants were male. Common comorbidities includded diabetes and hepatitis C virus co-infection. Participants had advanced drug-resistant TB and high baseline disease severity.
Recruitment	Participants were recruited prospectively a the National Center for Tuberculosis and Lung Diseases (NCTLD), Georgia, between October 2016 and February 2018. The NCTLD serves a a national referral centre for complex drug-resistant tuberculosis cases. All eligible participants who met inclusion criteria during the recruitment period were assessed and enrolled following written informed consent.
Ethics oversight	This study was conducted in accordance with the Declaration of Helsinki and all relevant ethical regulations. The study protocol and associated documents (approval code 870/01-17) was reviewed and approved by the Ethics Review Committee of the National Center for Tuberculosis and Lung Diseases (NCTLD), Georgia (IRB00007705, IORG0006411). All study participants received and reviewed the patient information sheet and provided written informed consent prior to enrolment. Participants were not compensated financially or otherwise for their participation in the study. The trial was registered at ClinicalTrials.gov (NCT02781909).

Note that full information on the approval of the study protocol must also be provided in the manuscript.

Field-specific reporting

Please select the one below that is the best fit for your research. If you are not sure, read the appropriate sections before making your selection.

☒ Life sciences ☐ Behavioural & social sciences ☐ Ecological, evolutionary & environmental sciences

For a reference copy of the document with all sections, see nature.com/documents/nr-reporting-summary-flat.pdf

Life sciences study design

All studies must disclose on these points even when the disclosure is negative.

Sample size	This was an exploratory phase IIa pilot study. No formal sample size calculation was performed. The sample size (n=28, n=14 per arm) was determined pragmaticalluy based on feasibility, regulatory considerations, and the objective of estimating the magnitude and direction of possible effects to inform the design of a subsequent adequately powered trial.
Data exclusions	No participants were excluded after allocation. Analyses followed and intention-to-treat approach including all allocated participants with available data. Missing data were due to sample availability at specific time points and were not excluded based on outcome.
Replication	This study was conducted as a single-centre pilot clinical trial. All experimental measurements -including cytokine assays, RNA sequencing, and lipid mediator analyses- were performed once per collected biological sample according to standardised protocols. No independent biological replication cohort was included, as the study was exploratory and hypothesis-generating. All planned assays were performed on available samples; sample availability varied across timepoints as reported in the manuscript. Findings are presented as preliminary results intended to inform future confirmatory studies.
Randomization	Although the approved protocol specified central concealed randomisation, allocation was implemented at the study site using simple alternation in a 1:1 ration due to an operational implementation error. The alternating sequence was applied consistently as recorded in the enrolment logs. Because allocation by alternation is predictable and does not ensure allocation concealment, selection bias cannot be excluded. This may have contributed to the observed baseline imbalance in disease extent and severity between arms, although such imbalances are also plausibly attributable to chance given the small sample size.
Blinding	The study was open-label. Participants and investigators were aware of treatment allocation, which may have introduced performance or assessment bias. Radiological assessments were performed blinded to treatment group. No placebo was used.

Reporting for specific materials, systems and methods

We require information from authors about some types of materials, experimental systems and methods used in many studies. Here, indicate whether each material, system or method listed is relevant to your study. If you are not sure if a list item applies to your research, read the appropriate section before selecting a response.

Materials & experimental systems

n/a	Involved in the study
☒	☐ Antibodies
☒	☐ Eukaryotic cell lines
☒	☐ Palaeontology and archaeology
☒	☐ Animals and other organisms
☐	☒ Clinical data
☒	☐ Dual use research of concern
☒	☐ Plants

Methods

n/a	Involved in the study
☒	☐ ChIP-seq
☒	☐ Flow cytometry
☒	☐ MRI-based neuroimaging

Clinical data

Policy information about clinical studies

All manuscripts should comply with the ICMJE guidelines for publication of clinical research and a completed CONSORT checklist must be included with all submissions.

Clinical trial registration

The trial was prospectively registered at ClinicalTrials.gov (NCT02781909) prior to participant enrolment.

Study protocol

The study protocol was approved (approval code 870/01-17) was reviewed and approved by the Ethics Review Committee of the National Center for Tuberculosis and Lung Diseases (NCTLD), Georgia (IRB00007705, IORG0006411). Protocol, including Patient information sheet, Consent form, and Statistical Analysis Plan, is available at https://doi.org/10.5281/zenodo.20342393.
The study was conducted in accordance with the Declaration of Helsinki and all relevant ethical regulations.

Data collection

Clinical and laboratory data were collected prospectively at the NCTLD, Georgia, using paper source documents and subsequenly entered ino an electronic case report form (REDCap version 12.2.11, hosted at the University of the Witwatersrand, South Africa). Microbiological and radiological assessments were performed as part of routine clinical management according to national and WHO guidelines. Laboratory assays for immunological analyses were conducted according to standardised protocols as described in the Methods section.

Outcomes

The primary efficacy outcome, as prespecified in the protocol, was the proportion of pilot study participants showing clinical and/or microbiological benefits from the new regimen relative to controls, evaluated using three prespecified outcome measurement sets: (i) microbiological (proportion with negative sputum culture at months 2 and 6, and time to stable sputum culture conversion up to month 6); (ii) radiological (chest X-ray changes at months 3 and 6); and (iii) final treatment outcomes per WHO definitions at month 6. Participants were considered cured and successfully treated when showing evidence of bacteriological clearance with no reversion and no evidence of failure. No composite endpoint was constructed, and each measurement set was analysed and reported independently. As a pilot study, all analyses were prespecified as exploratory and intended to indicate direction and magnitude of potential effects rather than to support formal hypothesis testing.

The secondary outcomes were to compare the proportion of study participants 1) who tolerated the new regimen, 2) showing differences in Health Quality of Life (HQoL), and 3) showing differences in immune responses.

Full details are described in the Methods section.

Plants

Seed stocks

N/A

Novel plant genotypes

N/A

Authentication

N/A
