# Supplementary material for: Adjunctive ibuprofen in pre-extensively drug-resistant and extensively drug-resistant tuberculosis: a phase IIA open-label pilot clinical trial
Source: Nat Commun. 2026 Jul 1;17:8185. doi: 10.1038/s41467-026-75148-9 (PMC13462894; doi:10.1038/s41467-026-75148-9)
Supplement: Supplementary file 3 [file 41467_2026_75148_MOESM3_ESM.pdf]

nature portfolio

Peer Review File

Adjunctive ibuprofen in pre-extensively drug-resistant and extensively drug-resistant tuberculosis: a Phase IIA open-label pilot clinical trial

Corresponding Author: Dr Cristina Vilaplana

This file contains all reviewer reports in order by version, followed by all author rebuttals in order by version.

Version 0:

Reviewer comments:

Reviewer #1

(Remarks to the Author)
This pilot randomized open-label study evaluates the safety and preliminary efficacy of adjunctive ibuprofen in pre-XDR and XDR TB. The authors rationale of immune modulation to reduce inflammation and potentially improve patient outcomes is interesting.

To further strengthen the manuscript for publication, a few issues require clarification and revision.

Major comments:
1. The standard of care for pre-XDR and XDR TB treatment is rather heterogenous. Comprising of both BPAL and non BPAL regime. (Do correct spelling error to pretomanid). I note that other drugs are also used with BPAL. And some patients used BL without Pto. This itself can skew the outcomes and limit the interpretations of this small trial.

This is exemplified in Supplementary table 2, Page 3: background regimens were not balanced between arms; linezolid-containing regimens were slightly more common in the ibuprofen arm (11/14) than in the control arm (9/14).

As patients with more severe TB often receive more aggressive, individualized regimens—and the ibuprofen group demonstrated worse baseline disease severity—the imbalance in regimen composition may reflect underlying clinical differences rather than random variation.

The current layout makes it difficult to compare background regimens between the two arms. Please consider reorganizing the table to present the control and ibuprofen regimens side-by-side (e.g., two parallel columns), so that regimen differences are easier to interpret.

2. Primary outcomes: There are actually 6 listed:
i) Proportion of negative sputum culture at month 2
ii) Proportion of negative sputum culture at month 6
iii) Time to sputum culture conversion
iv) Changes detected by Xray during follow-up at 3 months
v) Changes detected by Xray during follow-up at 6 months
vi) Final treatment outcomes up 6 months on TB treatment.
Can the authors clarify the above need for 6 primary outcomes?

3. What is the method of randomization? The method section (line 319): Alternation is not randomization, allocation is predictable and using the term “randomly assigned” is incorrect and misleading in CONSORT-compliant reporting. Likewise, how many patient were pre-screened or screened?

4. Supp Table 9 Baseline laboratory data. I note the ibuprofen arm seem to have lower albumin than the control arm, and this implies that the Ibu arm might have patient may have a worse baseline. This is also reflected in Supp Figure 1, showing a

slightly worse TB severity score in the ibuprofen arm. What is the baseline smear status, or time to positivity for both arms?

5. Please clarify if there is a data monitoring team and/or data safety monitoring board as this does not seem to be stated.

6. Page 14, line 329 onwards “Standard TB treatment”. Do note that standard TB treatment indicate the usual rifampicin, isoniazid, ethambutol and pyrazinamide for drug-susceptible TB. Which is not applicable for the preXDR/XDR population.

7. The authors did not mention how many doses of ibuprofen were taken and how these were accounted for each of the patients who are in the ibuprufen arm. (eg pill count etc).

8. Safety-related events, supplementary table 12, page 10: AEs were not formally graded using CTCAE. This reviewer currently does not know the severity of the AE. What does “lab results worsening” mean? I note most of the reported AEs seem to have resolved by month 2, which is when the intervention stopped. Some of these thus could possibly be related to Ibuprufen, such as anaemia and Acute Kidney Injury.

9. The study addresses an important gap in HDT for drug-resistant TB, and the safety and immune signatures results in particular, provide valuable insights. However, the manuscript would benefit from a stronger alignment between the stated limitations and the interpretation of findings.

While the authors clearly acknowledge the major methodological constraints (the open-label design, allocation by alternation, and heterogeneity of individualized second-line regimen), the Results and Discussion sections use phrasing such as “no differences were observed” for sputum conversion (line 128), radiological outcomes (line 136), and HQoL measures (line 158). Given the very small sample size, the substantial baseline imbalances outlined on page 5, and risk of selection/performance bias , such phrasing may inadvertently imply equivalence between treatment arms.

The median time to stable sputum culture conversion was four months in each group (Figure 3, Supplementary Table 11), though again these findings should be interpreted cautiously given the limited sample size and variability. I recommend reframing these statements to emphasize the imprecision and wide confidence intervals, rather than suggesting lack of effect. For example:

The wide confidence interval for the risk difference (RD = 18%, 95% CI –13 to 50) indicates substantial imprecision, and the small sample size limits meaningful between-group comparisons. By month 6, all participants with available data in both groups had negative sputum cultures (Figure 2).

10. Table 1, Page 22: Recommend marking statistically relevant imbalances (e.g., fever 67% vs 100%, SOB 40% vs 79%, baseline SGRQ scores, etc) with footnotes to improve clarity.

11. Figure 2, Page 25: Forest plot is informative but very small and small fonts for readers.

12. Minor Typo, Page 10 (line 244): To correct as “Similarly, trended toward” not “treaded”.

13. Throughout all Table and Figures, do define the acronyms. Eg. M2 - M6, Rx HQoL

14. Page 12 (line 284): “could have obscured modest adjunctive effects of ibuprofen” leans subtly toward assuming a beneficial effect that was masked. However there is no statistical significance and the authors should avoid overstating the effects of the adjunctive treatment.

15. Page 7, (line 172-178): The text currently states “decreases were more pronounced in the ibuprofen group” or “IFNG showed a more marked decrease”. Given the small sample size, baseline severity differences, and imbalance in linezolid-containing regimens, it is difficult to attribute observed differences in MLR or cytokine trajectories to ibuprofen itself. I recommend clarifying that these findings are exploratory and that reductions in inflammatory markers likely reflect underlying regimen effects and natural disease trajectory, rather than treatment-specific effects.

Reviewer #2

(Remarks to the Author)
I co-reviewed this manuscript with one of the reviewers who provided the listed reports. This is part of the Nature Communications initiative to facilitate training in peer review and to provide appropriate recognition for Early Career Researchers who co-review manuscripts.

Reviewer #3

(Remarks to the Author)
In this article, Fonseca and co-authors present the results of a pilot randomized controlled trial performed in Georgia to evaluate whether adding ibuprofen to standard treatment could improve outcomes in adults with pre-XDR or XDR TB. Overall, there was no difference between the two treatment arms in terms of treatment outcome, sputum culture conversion or radiological recovery. Safety profiles were also comparable. However, the addition of ibuprofen seemed to reduce several inflammatory markers.

The article is overall clear and well written. The study objective is well detailed and has extremely high relevance for the control of tuberculosis; identifying an effective HDT could represent a major breakthrough in ongoing efforts to improve TB treatment. This small, pilot study is not powered to draw any conclusion on efficacy or safety but supports moving forward with the investigation of ibuprofen in light of the absence of major safety signals and of the confirmed anti-inflammatory effect of ibuprofen.

I have the following major remarks for the authors:
- Trial outcomes and endpoints are not clearly defined. Their description should only be included in the Methods section and, for primary outcomes, in the Abstract: as of now, some details are included in the Results. For each outcome, a detailed endpoint should be described: for instance, you include time to culture conversion as a primary endpoint but do not define it in sufficient detail in the methods, where there is a reference to time to "stable" culture conversion. What does it mean? The definition of tolerance of the regimen is also complex and should be better defined.
- The rationale for only including pre-XDR and XDR-TB patients, and not all RR-TB, is not provided and should be added to the Introduction; please also add there the definitions of pre-XDR and XDR-TB that you used in the trial
- Details on the treatment regimens received by participants in the two arms should be presented in the Results, and eventual imbalances between the two groups should be discussed
- Please present Safety results, at least for the main endpoints, in the Result section; defining them as "well tolerated" pertains more to the discussion than to the results; similar remark for the Abstract
- please provide additional information on why the centralized allocation was not performed and replaced by simple alternation at site level; in the Discussion, I suggest that you elaborate on the bias that could have been introduced, for instance in explaining the imbalance in terms of disease extent between the two groups

In addition, some minor comments:
- Please add IQR for time to culture conversion
- in line 145, the % of cure in the control should be 71 (not 77)
- in the Discussion, you comment on the 4 months of time to culture conversion as what is commonly observed in DR-TB: this is not true with most modern DR-TB regimens, where time to culture conversion tends to be faster
- Lines 215-216: saying that "Radiological assessments revealed no differences in chest X-ray evolution" is not completely correct; i suggest to use "similar results" instead
- Overall, it is sometimes difficult to follow the Discussion due to repeated backs and forths between the discussion of efficacy and safety results; I suggest to reorganise it
- in the Conclusion, I suggest to be more straightforward: in a nuthsell, results support safety and immuno-modulating activity, but not clinical advantage

Reviewer #4

(Remarks to the Author)
To add cheap and effective host directed therapy to anti- tuberculosis drug treatment for pre-XDR and XDR tuberculosis to help achieve more efficient drug cure with less residual lung damage would be a useful adjunct to the drug regimens currently on offer. This small study used ibuprofen in a group of 14 patients with pre XDE/XDR tuberculosis compared to 14 controls. Although the patients receiving ibuprofen added to standard of care TB drug treatment did not show significant differences from the control group in terms of sputum clearance of Mtb or improvements seen on chest xrays, the ibuprofen group had more extensive disease at recruitment. Ibuprofen was able to reduce the concentration of some of the cytokines measured and modulate gene expression signatures. The trends seen for some of the parameters measured and the fact that the ibuprofen was found to be safe, indicate that perhaps further larger studies should be conducted.

Inflammatory markers were evaluated using the neutrophil:lymphocyte (NLR) and the monocyte:lymphocyte (MLR) ratios, and the systemic immune inflammation index (NLR multiplied by platelet count). Were any measurements of inflammatory markers such as CRP available? Why was a Millipore T cell panel used to measure the circulating pro-and anti-inflammatory inflammation markers? IFNg is the signature cytokine for Type 1 T cell responses and IL-6, IL-1b or even TNF might be better markers of inflammation.

In Figure 1, groups of 14 individuals were recruited but of these only 10 or 11 completed follow up. In the Analysis boxes the numbers analysed at 2 and 6 months could be included? Why were 14 individuals in each group available for analysis of final treatment outcomes at month 6, but sputum cultures or xrays were only measured on 10/11 individuals per group at months 2/3 and 6?

In Figure 3 please add the p value obtained in the statistical analysis.

Minor suggestions
Line 80, correct to COX-2.
Line 91, suggest rewording to “ibuprofen has never previously been tested”.

Line 107, suggest rewording to “the ibuprofen groups had a higher proportion of female…”
Line 108, suggest rewording to …”with the proportions…
Line 144, suggest rewording to…”with 71% achieving …”
Line 217, suggest rewording to “..initially presenting…
Line 240, suggest rewording to “progression in TB” (delete “also”).
Line 251, IL-1B
Line 314, does this mean, “the current trial”?
Line 565, the title of this figure could be changed to “Immune responses changes over time”.

Version 1:

Reviewer comments:

Reviewer #3

(Remarks to the Author)
I am grateful to the authors for their point by point replies. A few of my major comments are however still insufficiently addressed:
- in the Study Outcomes (Methods): thanks for the modifications. This is stil difficult to follow. The definition of the primary endpoint is not clear to me. I understand (more or less) the three components of the primary outome, but how do you incorporate them to define a final outcome? I would not be able to assign this endpoint, based on this description. For the secondary endpoint of tolerance: do you mean that experiencing any adverse event would define a bad tolerance? Which are the "protocol-defined" AEs?
- in the Study Outcomes (Methods): please clarify the sentence "Adverse events were not graded using CTCAE". Do you mean that they were not graded at all?
- in the Results, for Safety: you refer to Figure 2, which is not presenting safety. Please include a Table in the main manuscript with the safety results, This is a key information for the readership

Reviewer #4

(Remarks to the Author)
This small study suggests that Ibuprofen when added to drug treatment regimens for XDR/PreXDR TB, may modulate inflammatory responses and gene expression signatures, although no clinical benefit was shown. The revised manuscript has provided additional information on the laboratory tests that were performed, and why some other markers such as CRP that would have provided a direct measurement of inflammation, were not used.

In the revised ms the revision of the final section of the Discussion states "Finally, stronger evidence and consensus on patient selection will be essential before such strategies can be incorporated into clinical recommendations.mplies that Ibuprofen will subsequently be shown to have clinical benefit".
So far no evidence for clinical benefit has been shown, and this sentence should be rewritten, to indicate that if it is subsequently shown to induce clinical benefit, then it could be a useful adjunctive therapy.

Open Access This Peer Review File is licensed under a Creative Commons Attribution 4.0 International License, which permits use, sharing, adaptation, distribution and reproduction in any medium or format, as long as you give appropriate credit to the original author(s) and the source, provide a link to the Creative Commons license, and indicate if changes were made.
In cases where reviewers are anonymous, credit should be given to 'Anonymous Referee' and the source.
The images or other third party material in this Peer Review File are included in the article's Creative Commons license, unless indicated otherwise in a credit line to the material. If material is not included in the article's Creative Commons license and your intended use is not permitted by statutory regulation or exceeds the permitted use, you will need to obtain permission directly from the copyright holder.
To view a copy of this license, visit https://creativecommons.org/licenses/by/4.0/

Answers to the reviewers' comments

Reviewer #1:

This pilot randomized open-label study evaluates the safety and preliminary efficacy of adjunctive ibuprofen in pre-XDR and XDR TB. The authors rationale of immune modulation to reduce inflammation and potentially improve patient outcomes is interesting.

To further strengthen the manuscript for publication, a few issues require clarification and revision.

1. Comment

1. The standard of care for pre-XDR and XDR TB treatment is rather heterogenous. Comprising of both BPAL and non BPAL regime. (Do correct spelling error to pretomanid). I note that other drugs are also used with BPAL. And some patients used BL without Pto. This itself can skew the outcomes and limit the interpretations of this small trial.

This is exemplified in Supplementary table 2, Page 3: background regimens were not balanced between arms; linezolid-containing regimens were slightly more common in the ibuprofen arm (11/14) than in the control arm (9/14).

As patients with more severe TB often receive more aggressive, individualized regimens—and the ibuprofen group demonstrated worse baseline disease severity—the imbalance in regimen composition may reflect underlying clinical differences rather than random variation.

The current layout makes it difficult to compare background regimens between the two arms. Please consider reorganizing the table to present the control and ibuprofen regimens side-by-side (e.g., two parallel columns), so that regimen differences are easier to interpret.

Answer: We agree with the reviewer that the background DR-TB regimens were heterogeneous, reflecting programmatic individualisation for pre-XDR/XDR TB, and that in a small pilot study such heterogeneity and minor between-arm imbalances can limit interpretation of between-group comparisons.

To facilitate interpretation, we have reorganised Supplementary Table 2 to present background regimens side-by-side by trial arm, enabling direct comparison of regimen composition. As highlighted by the reviewer, linezolid-containing regimens were slightly more common in the ibuprofen arm (11/14) than in the control arm (9/14). Importantly, key agents such as bedaquiline were similarly represented between arms (7/14 in each group). Heterogeneity in background regimens is now described and clarified in the Results section *(page 5, lines 106-109 of the clean version of the manuscript)*). Moreover, we have clarified in the Methods that regimens were DST-guided and individualised in accordance with contemporaneous national and WHO guidance during a period of evolving access to newer DR-TB agents; therefore, a uniform BPAL/BPaL regimen backbone was not implemented in this pilot *(page 16, lines 360-365 of the clean version of the manuscript)*). We have also ensured that it is explicitly addressed in the Limitations section, where we have described treatment regimen heterogeneity in a neutral manner and emphasise that this introduces uncertainty in attributing the observed clinical and immunological differences specifically to ibuprofen *(page 13, lines 304-308 of the clean version of the manuscript).*

In addition, we have corrected the spelling of pretomanid throughout the manuscript and Supplementary Information.

2. Comment

Primary outcomes: There are actually 6 listed:

i) Proportion of negative sputum culture at month 2

ii) Proportion of negative sputum culture at month 6

iii) Time to sputum culture conversion

iv) Changes detected by Xray during follow-up at 3 months

v) Changes detected by Xray during follow-up at 6 months

vi) Final treatment outcomes up 6 months on TB treatment.

Can the authors clarify the above need for 6 primary outcomes?

Answer: We agree with the reviewer that the way outcomes were presented in the submitted manuscript could be misinterpreted as specifying multiple co-primary endpoints. This was not our intention.

As prespecified in the approved clinical trial protocol, the study had a single primary efficacy outcome: *the percentage of pilot study participants who show clinical or microbiological benefit of the new regimen relative to controls*. In the protocol, this primary outcome was assessed through a set of prespecified outcome measurements, rather than six independent primary endpoints. These outcome measurements include microbiological measures (percentage of negative sputum culture at month 2 and time to stable sputum culture conversion up to month 6 (≥2 consecutive negative cultures), radiological evaluation (chest X-ray at baseline and every 3 months), and interim/final treatment outcomes according to WHO definitions through month 6. For transparency, we note that in the ClinicalTrials.gov registration, the primary efficacy outcome is presented in a disaggregated format because the registry requires outcomes to be entered as separate items/fields. The listed items (microbiological measures at M2/M6, time to stable culture conversion, radiological follow-up assessments, and WHO treatment outcomes) correspond to the prespecified outcome measurements used to assess the single primary efficacy outcome (clinical and/or microbiological benefit) as described in the approved protocol, rather than representing multiple independent primary endpoints.

We have revised the manuscript accordingly to make this structure explicit in the M&M section *(page 17, lines 384-400 of the clean version of the manuscript*), we now clearly state that there is one primary outcome, assessed using multiple prespecified outcome measurements, and we have reorganised the "Study Outcomes" section to avoid any implication of six co-primary endpoints. We have also added a clarifying sentence in the manuscript noting that these outcome measurements were listed as separate items in the ClinicalTrials.gov registry due to registry formatting requirements, but collectively operationalise the single prespecified primary efficacy outcome.

3. Comment

What is the method of randomization? The method section (line 319): Alternation is not randomization, allocation is predictable and using the term "randomly assigned" is incorrect and misleading in CONSORT-compliant reporting. Likewise, how many patient were pre-screened or screened?

Answer: We thank the reviewer for highlighting this important point. We agree that allocation by alternation does not constitute true randomisation because allocation is predictable and not concealed.

The approved protocol specified central concealed randomisation; however, due to an operational implementation error at the single recruiting site, alternation was used in practice (protocol deviation). We reviewed site enrolment logs and confirmed that the alternating sequence was applied consistently as recorded. We have therefore revised the Methods section to explicitly state that allocation was implemented at the recruiting site by simple alternation in a 1:1 ratio and that the study was open-label (no placebo) (Participant allocation subsection; Page 15-16, lines 353-361). As alternation does not ensure allocation concealment and may increase the risk of selection bias, this limitation is now stated explicitly and discussed in the Limitations section *(page 13, lines 302-307 of the clean version of the manuscript*); and the term "randomly assigned" has been removed throughout the manuscript to ensure CONSORT-consistent reporting.

Regarding participant flow, we have clarified the number of patients screened and enrolled in the CONSORT flow diagram (Figure 1) and updated the Figure 1 legend accordingly. A total of 28 patients were screened, and all 28 were enrolled and allocated (14 per arm).

4. Comment

Supp Table 9 Baseline laboratory data. I note the ibuprofen arm seem to have lower albumin than the control arm, and this implies that the Ibu arm might have patient may have a worse baseline. This is also reflected in Supp Figure 1, showing a slightly worse TB severity score in the ibuprofen arm. What is the baseline smear status, or time to positivity for both arms?

Answer: We agree with the reviewer that there were baseline differences in disease severity between arms, including lower albumin levels in the ibuprofen arm, reinforcing evidence of worse baseline clinical status. These differences are now explicitly described in the Results section (*pages 5-6, lines 121-131 of the clean version of the manuscript;* Table 1, Supplementary Table 9) and are acknowledged in the Limitations paragraph of the Discussion (*page 13, lines 299-311*) as a potential source of confounding in this small pilot study; therefore, between-group comparisons should be interpreted cautiously. In line with CONSORT guidance, baseline characteristics are presented descriptively and no formal statistical testing of baseline differences was performed.

Regarding baseline microbiological burden, baseline sputum smear status (including smear grading) and baseline time-to-positivity were not recorded/available in this pilot trial; therefore, we are unable to report these parameters by arm. We have added this clarification to the Methods section (Demographic and Clinical Data Collection) *(page 18, lines 421-423 of the clean version of the manuscript*).

5. Comment

Please clarify if there is a data monitoring team and/or data safety monitoring board as this does not seem to be stated.

Answer: This was a small pilot trial conducted under severe funding constraints and no independent Data Safety Monitoring Board (DSMB) was constituted. Safety monitoring was instead conducted within the existing clinical governance structure of the NCTLD. Specifically, since 2010 the NCTLD has maintained a Central Clinical Council for the Treatment of Drug-Resistant Tuberculosis, whose responsibilities include oversight of MDR/XDR-TB management and safety-related monitoring. Adverse events and laboratory abnormalities were monitored throughout follow-up by the clinical trial team as part of routine clinical care and were reviewed within this framework. We have added this clarification to the Methods section *(page 17, lines 406-410 of the clean version of the manuscript*).

6. Comment

Page 14, line 329 onwards "Standard TB treatment". Do note that standard TB treatment indicate the usual rifampicin, isoniazid, ethambutol and pyrazinamide for drug-susceptible TB. Which is not applicable for the preXDR/XDR population.

Answer: We agree with the reviewer that the term "standard TB treatment" is inappropriate in the context of pre-XDR/XDR tuberculosis, as it is commonly used to refer to first-line drug-susceptible regimens (rifampicin, isoniazid, ethambutol, and pyrazinamide). To address this, we have revised the Methods section to clarify that that all participants received individualised background DR-TB regimens, prescribed by the treating physician in accordance with contemporaneous national and WHO guidance and tailored to drug-resistance patterns and clinical characteristics *(page 16, lines 363-371 of the clean version of the manuscript*). In addition, we have replaced the wording "standard TB treatment" throughout the manuscript with "background anti-TB regimen" (or "background DR-TB regimen", where appropriate) to avoid ambiguity and ensure accurate representation of the treatment context.

7. Comment

The authors did not mention how many doses of ibuprofen were taken and how these were accounted for each of the patients who are in the ibuprofen arm. (eg pill count etc).

Answer: Ibuprofen was administered as 400 mg (one tablet) orally once daily for 2 months and was provided by the Pharmacy Department of the NCTLD and delivered under directly observed therapy (DOT) as part of routine programmatic care; therefore, adherence was monitored via DOT rather than pill counts. We have clarified this in the Methods section *(page 16, lines 371-377 of the clean version of the manuscript*) and added the statement that completion or non-completion of the planned 2-month ibuprofen course is summarised in Supplementary Table 10, which reports course completion status for each participant.

8. Comment

Safety-related events, supplementary table 12, page 10: AEs were not formally graded using CTCAE. This reviewer currently does not know the severity of the AE. What does "lab results worsening" mean? I note most of the reported AEs seem to have resolved by month 2, which is when the intervention stopped. Some of these thus could possibly be related to Ibuprofen, such as anaemia and Acute Kidney Injury.

Answer: Safety monitoring in this pilot trial followed the protocol-defined tolerability/safety framework. As prespecified in the protocol, safety-related events were recorded throughout follow-up and included: 1) worsening and/or exacerbation of TB (clinical worsening), 2) conversion from smear-negative to smear-positive, 3) conversion from culture-negative to culture-positive, 4) worsening in chest X-ray, 5) any alteration of routine blood tests (defined using the reference values of the laboratory methods used at the NCTLD), and 6) any event worsening the patient's health status. Safety events were therefore captured as protocol-specified safety-related events. However, we agree that events were not graded using CTCAE, which limits standardised severity classification; we have added this clarification to the Methods section *(page 17-18, lines 400-410 of the clean version of the manuscript*).

We also agree that the term "lab results worsening" is non-specific. To improve clarity, we have revised Supplementary Table 12 (where affected parameters, timepoint, and recovery were already specified for each

event) to use the wording "laboratory test alteration", and we added a table definition in the legend indicating that this refers to clinically relevant abnormalities detected on routine monitoring.

Regarding potential relatedness to ibuprofen, we agree that some laboratory test alterations occurred during the 2-month ibuprofen administration period. However, events were observed in both arms, were not CTCAE graded, and causality was not formally adjudicated; given the small sample size and heterogeneous background regimens (including linezolid exposure), attribution to ibuprofen versus intercurrent illness or concomitant drugs remains uncertain. Even if, importantly, no safety-related event required intervention, and events were transient with recovery (documented in Supplementary Table 12), following the reviewer's comment we have revised the Discussion accordingly and included a paragraph to reflect this cautious interpretation *(pages 10-11, lines 236-240 of the clean version of the manuscript*).

9. Comment

The study addresses an important gap in HDT for drug-resistant TB, and the safety and immune signatures results in particular, provide valuable insights. However, the manuscript would benefit from a stronger alignment between the stated limitations and the interpretation of findings.

While the authors clearly acknowledge the major methodological constraints (the open-label design, allocation by alternation, and heterogeneity of individualized second-line regimen), the Results and Discussion sections use phrasing such as "no differences were observed" for sputum conversion (line 128), radiological outcomes (line 136), and HQoL measures (line 158). Given the very small sample size, the substantial baseline imbalances outlined on page 5, and risk of selection/performance bias, such phrasing may inadvertently imply equivalence between treatment arms.

The median time to stable sputum culture conversion was four months in each group (Figure 3, Supplementary Table 11), though again these findings should be interpreted cautiously given the limited sample size and variability. I recommend reframing these statements to emphasize the imprecision and wide confidence intervals, rather than suggesting lack of effect. For example:

The wide confidence interval for the risk difference (RD = 18%, 95% CI −13 to 50) indicates substantial imprecision, and the small sample size limits meaningful between-group comparisons. By month 6, all participants with available data in both groups had negative sputum cultures (Figure 2).

Answer: We thank the reviewer for this important comment. We agree that, given the small sample size, baseline imbalances, and potential selection and performance biases, phrasing such as "no differences were observed" could be misinterpreted as evidence of equivalence. We have therefore revised the Results and Discussion sections to explicitly emphasise the imprecision of the estimates and the limited capacity for between-group inference. In the Results section, we now highlight the wide confidence intervals for key outcomes and explicitly discuss their implications for sputum culture conversion and radiological changes, reframing these findings as descriptive observations rather than evidence of absence of effect.

10. Comment

Table 1, Page 22: Recommend marking statistically relevant imbalances (e.g., fever 67% vs 100%, SOB 40% vs 79%, baseline SGRQ scores, etc) with footnotes to improve clarity

Answer: Following the reviewer's suggestion, we have added footnote markers in Table 1 to highlight baseline variables showing notable between-arm differences (e.g., fever, shortness of breath, baseline SGRQ score, TB

severity score). As recommended by CONSORT, baseline characteristics are presented descriptively and no hypothesis testing is reported for baseline comparisons; this is now stated in the Table 1 legend.

11. Comment

Figure 2, Page 25: Forest plot is informative but very small and small fonts for readers.

Answer: We thank the reviewer for this comment. We agree that when Figure 2 is embedded in the manuscript Word/PDF for review, the forest plot labels may appear small due to scaling and document compression. To address this, we have ensured that Figure 2 is provided as an independent high-resolution figure file in the revised submission, in which all labels are clearly legible at full resolution. The figure content is unchanged.

12. Comment

Minor Typo, Page 10 (line 244): To correct as "Similarly, trended toward" not "treaded".

Answer: Thank you for pointing this out, we have corrected this typo.

13. Comment

Throughout all Table and Figures, do define the acronyms. Eg. M2 - M6, Rx HQoL

Answer: Figure and table captions have been updated to define all abbreviations, as per the reviewer's suggestion.

14. Comment

Page 12 (line 284): "could have obscured modest adjunctive effects of ibuprofen" leans subtly toward assuming a beneficial effect that was masked. However there is no statistical significance and the authors should avoid overstating the effects of the adjunctive treatment.

Answer: We do agree with the reviewer that the original phrasing could be interpreted as implying a beneficial adjunctive effect. Following his/her suggestion, as other reviewer's comments, we have therefore revised the text to avoid overstatement and to frame the findings more cautiously in light of the study's limitations. The specific sentence has been amended to:

"[…] background anti-tuberculosis regimens were heterogeneous and showed some imbalance between arms, which may have influenced sputum culture conversion and other outcomes and increases uncertainty in attributing observed clinical or immunological differences specifically to ibuprofen. Future trials using unified background regimens would better isolate adjunctive drug-specific effects." *(page 13, lines 306-311 of the clean version of the manuscript*).

15. Comment

Page 7, (line 172-178): The text currently states "decreases were more pronounced in the ibuprofen group" or "IFNG showed a more marked decrease". Given the small sample size, baseline severity differences, and imbalance in linezolid-containing regimens, it is difficult to attribute observed differences in MLR or cytokine trajectories to ibuprofen itself. I recommend clarifying that these findings are exploratory and that reductions in inflammatory markers likely reflect underlying regimen effects and natural disease trajectory, rather than treatment-specific effects.

Answer: In response to the reviewer's comment, we have revised the Results section to remove treatment-attributive wording (e.g., "more pronounced decrease", "more marked decrease") and replaced it with neutral, descriptive language (i.e., "numerically larger decreases observed in the ibuprofen arm at some timepoints"). *(Page 8, lines 182-186 of the clean version of the manuscript).* We have also revised the Discussion to explicitly state that these immunological patterns are hypothesis-generating and cannot be attributed specifically to ibuprofen in this small pilot trial, and that mechanistic interpretation has been reframed as a possible explanation rather than a treatment-specific conclusion *(Page 11, lines 265-266 of the clean version of the manuscript).* Finally, we have strengthened the Limitations section to more clearly articulate how the pilot sample size, baseline severity differences, and heterogeneity in background regimens constrain causal interpretation. *(Page 13, lines 299-311 of the clean version of the manuscript).*

Reviewer #2:

I co-reviewed this manuscript with one of the reviewers who provided the listed reports. This is part of the Nature Communications initiative to facilitate training in peer review and to provide appropriate recognition for Early Career Researchers who co-review manuscripts.

Manuscript reference: NCOMMS-25-84890

Reviewer #3:

In this article, Fonseca and co-authors present the results of a pilot randomized controlled trial performed in Georgia to evaluate whether adding ibuprofen to standard treatment could improve outcomes in adults with pre-XDR or XDR TB. Overall, there was no difference between the two treatment arms in terms of treatment outcome, sputum culture conversion or radiological recovery. Safety profiles were also comparable. However, the addition of ibuprofen seemed to reduce several inflammatory markers.

The article is overall clear and well written. The study objective is well detailed and has extremely high relevance for the control of tuberculosis; identifying an effective HDT could represent a major breakthrough in ongoing efforts to improve TB treatment. This small, pilot study is not powered to draw any conclusion on efficacy or safety but supports moving forward with the investigation of ibuprofen in light of the absence of major safety signals and of the confirmed anti-inflammatory effect of ibuprofen.

I have the following major remarks for the authors:

1. Major comment

Trial outcomes and endpoints are not clearly defined. Their description should only be included in the Methods section and, for primary outcomes, in the Abstract: as of now, some details are included in the Results. For each outcome, a detailed endpoint should be described: for instance, you include time to culture conversion as a primary endpoint but do not define it in sufficient detail in the methods, where there is a reference to time to "stable" culture conversion. What does it mean? The definition of tolerance of the regimen is also complex and should be better defined.

Answer:

We agree with the reviewer that outcomes and endpoint definitions should be clearly and consistently presented in the Methods, with the Results limited to reporting findings. This issue has been addressed through revisions made in response to this and other reviewers' comments.

Accordingly, we have revised the Study Outcomes subsection in Methods to clearly describe the outcome framework and to avoid any implication of multiple co-primary endpoints. As prespecified in the protocol, the study had a single primary efficacy outcome, operationalised through prespecified outcome measurements encompassing microbiological, radiological, and clinical domains. We have also added an explicit clarification that the disaggregated listing of outcomes in the ClinicalTrials.gov entry reflects registry formatting requirements rather than the presence of multiple independent primary endpoints (Pages 16–17, lines 380–396 of the clean manuscript).

In the same section, we have added a clear definition of time to stable sputum culture conversion (tSCC). Stable culture conversion is defined as the time from treatment initiation to the first of at least two consecutive sputum cultures negative for *Mycobacterium tuberculosis*, with follow-up censored at month 6.

We have also clarified the definition of tolerability/safety to improve readability and reproducibility. As prespecified in the protocol, tolerability was assessed using protocol-defined safety-related events recorded throughout follow-up, including clinical worsening, microbiological or radiological worsening, vital-parameter derangements, and clinically relevant alterations in routine laboratory tests. This framework is now described more explicitly in the Methods, where we have expanded the paragraph addressing tolerability assessment. We additionally state that adverse events were not graded according to CTCAE criteria (Pages 17–18, lines 400–410 of the clean manuscript).

Finally, we reviewed the Results section to ensure that endpoint definitions are not introduced there and are presented exclusively in the Methods. We have also completely rewritten the abstract to comply with journal requirements and to reflect the reviewers' recommendations.

2. Major comment

The rationale for only including pre-XDR and XDR-TB patients, and not all RR-TB, is not provided and should be added to the Introduction; please also add there the definitions of pre-XDR and XDR-TB that you used in the trial.

Answer: Following the reviewer's suggestion, we have revised the Introduction to clarify that, as a small pilot study primarily designed to assess feasibility/safety and biological activity in the most complex DR-TB setting managed at the national referral centre, we focused on patients with advanced drug resistance (pre-XDR/XDR-TB) *(Page 4, lines 92-94 of the clean version of the manuscript)*. We also ensured that the operational definitions used in the trial are clearly stated in the Methods section *(Page 16 lines 363-371 of the clean version of the manuscript)*.

3. Major comment

Details on the treatment regimens received by participants in the two arms should be presented in the Results, and eventual imbalances between the two groups should be discussed

Answer: Following the reviewer's suggestion, we have added an explicit description of the individualised background DR-TB regimens in the Results, including between-arm differences. Specifically, we now state that regimens were heterogeneous and we listed them (Supplementary Table 2), with linezolid-containing regimens slightly more frequent in the ibuprofen arm (11/14 vs 9/14), while bedaquiline use was similar between arms (7/14 in each group) *(Page 5, lines 103-108 of the clean version of the manuscript)*. To facilitate direct comparison, Supplementary Table 2 has also been reorganised to present background regimens side-by-side by trial arm. Finally, we discuss background regimen heterogeneity and minor between-arm imbalances as a limitation that increases uncertainty in attributing between-group differences to ibuprofen *(Page 13, lines 307-311 of the tracked changed version of the manuscript)*.

4. Major comment

Please present Safety results, at least for the main endpoints, in the Result section; defining them as "well tolerated" pertains more to the discussion than to the results; similar remark for the Abstract

Answer: We agree with the reviewer that the presentation of safety findings could be clearer and that interpretive wording should be reserved for the Discussion. Following this comment, we have revised the manuscript so that the main safety findings are summarised explicitly in the Results section, stating that the incidence of protocol-defined safety-related events was similar between arms, that laboratory test alterations were the most frequent events, and that events were transient and required no intervention, *(Page 7, lines 158-162 of the clean version of the manuscript)* with full details provided in Supplementary Table 12. In addition, we have clarified in the Methods that adverse events were not graded using CTCAE *(Page 17-18, lines 400-410 of the clean version of the manuscript)*, and we have expanded the Discussion to note that causality was not formally adjudicated and that attribution to ibuprofen versus background regimens and/or intercurrent illness is therefore uncertain *(Page 10, lines 263-240 of the clean version of the manuscript)*. The abstract has also been updated to consider all the reviewers' comments.

5. Major comment

Please provide additional information on why the centralized allocation was not performed and replaced by simple alternation at site level; in the Discussion, I suggest that you elaborate on the bias that could have been introduced, for instance in explaining the imbalance in terms of disease extent between the two groups

Answer: The approved protocol specified central concealed randomisation; however, due to an operational implementation error at the single recruiting site, central allocation was not implemented, and participants were allocated by simple alternation in a 1:1 sequence (protocol deviation). We have revised the Methods to describe this process transparently, clarified that allocation was open-label, and removed the term "randomly assigned" throughout to ensure CONSORT-consistent reporting *(Pages 15-16, lines 353-361 of the clean version of the manuscript).*

We have also expanded the limitations paragraph in the Discussion to explicitly acknowledge that alternation does not ensure allocation concealment, which may increase the risk of selection bias. We note that this could have contributed to the observed baseline imbalance in disease severity between arms; however, given the very small sample size of this pilot study, such imbalances are also consistent with chance variation *(Page 13, lines 302-307 of the clean version of the manuscript).*

1. Minor Comment

Please add IQR for time to culture conversion

Answer: Following the reviewer's suggestion, IQR for time to SCC has been included in Results section. *(Page 6, lines 133-140 of the clean version of the manuscript).*

2. Minor Comment

In line 145, the % of cure in the control should be 71 (not 77)

Answer: Following the reviewer's observation, we have amended the error. *(Page 7, line 154 of the clean version of the manuscript).*

3. Minor Comment

In the Discussion, you comment on the 4 months of time to culture conversion as what is commonly observed in DR-TB: this is not true with most modern DR-TB regimens, where time to culture conversion tends to be faster

Answer: We agree with the reviewer that time to sputum culture conversion is typically shorter with contemporary standardised DR-TB regimens, particularly bedaquiline-based short-course regimens. Our cohort consisted of adults with pre-XDR/XDR-TB managed with individualised, heterogeneous longer regimens and high baseline disease burden, which may contribute to longer conversion times. We have therefore revised the Discussion accordingly, which now reads:

"Similar percentages of participants in both treatment arms achieved negative sputum cultures at months 2 and 6. The median time to stable SCC was four months in each group, which is slower than typically reported with contemporary standardised DR-TB regimens, particularly those containing bedaquiline [15,16]. However, this finding may be consistent with patients with advanced drug resistance managed with individualised longer regimens and a high baseline disease burden, which may contribute to longer times to culture conversion[15]" *(Page 10, lines 226-232 of the clean version of the manuscript).*

4. Minor Comment

Lines 215-216: saying that "Radiological assessments revealed no differences in chest X-ray evolution" is not completely correct; i suggest to use "similar results" instead

Answer: We agree with the reviewer that the original wording could be interpreted as implying equivalence. We have therefore revised the text to state that radiological assessments showed “similar results” between groups, rather than “no differences” *(Page 10, line 232-233 of the clean version of the manuscript).*

5. Minor Comment

Overall, it is sometimes difficult to follow the Discussion due to repeated backs and forths between the discussion of efficacy and safety results; I suggest to reorganise it.

Answer: Following the suggestions of this reviewer and others, we have substantially revised and reorganised the Discussion section to improve clarity, coherence, and flow, incorporating all suggested changes to enhance overall readability.

6. Minor Comment

In the Conclusion, I suggest to be more straightforward: in a nuthsell, results support safety and immuno-modulating activity, but not clinical advantage

Answer: Following the reviewer’s suggestion, we have revised the Conclusion to provide a clearer summary in a nutshell. The updated text now emphasises that this pilot trial supports the feasibility and short-term safety of adjunctive ibuprofen and shows immunomodulatory signals, but did not demonstrate a clinical advantage, and therefore supports further evaluation in adequately powered placebo-controlled trials *(Page 14, lines 322-331 of the clean version of the manuscript).*

Reviewer #4

To add cheap and effective host directed therapy to anti- tuberculosis drug treatment for pre-XDR and XDR tuberculosis to help achieve more efficient drug cure with less residual lung damage would be a useful adjunct to the drug regimens currently on offer. This small study used ibuprofen in a group of 14 patients with pre XDE/XDR tuberculosis compared to 14 controls. Although the patients receiving ibuprofen added to standard of care TB drug treatment did not show significant differences from the control group in terms of sputum clearance of Mtb or improvements seen on chest xrays, the ibuprofen group had more extensive disease at recruitment. Ibuprofen was able to reduce the concentration of some of the cytokines measured and modulate gene expression signatures. The trends seen for some of the parameters measured and the fact that the ibuprofen was found to be safe, indicate that perhaps further larger studies should be conducted.

1. Major comment

Inflammatory markers were evaluated using the neutrophil:lymphocyte (NLR) and the monocyte:lymphocyte (MLR) ratios, and the systemic immune inflammation index (NLR multiplied by platelet count). Were any measurements of inflammatory markers such as CRP available? Why was a Millipore T cell panel used to measure the circulating pro-and anti-inflammatory inflammation markers? IFNg is the signature cytokine for Type 1 T cell responses and IL-6, IL-1b or even TNF might be better markers of inflammation.

Answer: We utilized cell-derived composite inflammation indices (NLR, MLR, and SII) because they are highly feasible in resource-limited programmatic settings, requiring only a standard full blood count. These markers are inexpensive, do not require additional laboratory infrastructure, and have been widely validated in literature as robust correlates of TB disease severity and treatment response. Furthermore, while CRP is a valuable marker, we could not include it in this analysis as it was not routinely available at the study site during the study period. Regarding the circulating cytokines assessed, these were quantified using a validated multiplex bead-based platform (MILLIPLEX MAP high sensitivity panel), selected for its suitability with limited plasma volume and for enabling simultaneous measurement of a broad panel of inflammatory and regulatory cytokines relevant to TB immunopathogenesis. The multiplex panel included IL-6, IL-1β and TNF, in addition to IFNG and other cytokines (IL-10, IL-12, IL-17A, IL-2, IL-4, IL-8). However, consistent with the reviewer's point, interpretation of cytokine trajectories remains exploratory/hypothesis-generating and is not presented as treatment-attributive.

In order to clarify all these issues raised by the reviewer, we have ensured the Methods section includes now all these points *(Page 18-19, lines 429-447 of the clean version of the manuscript).*

2. Major comment

In Figure 1, groups of 14 individuals were recruited but of these only 10 or 11 completed follow up. In the Analysis boxes the numbers analysed at 2 and 6 months could be included? Why were 14 individuals in each group available for analysis of final treatment outcomes at month 6, but sputum cultures or xrays were only measured on 10/11 individuals per group at months 2/3 and 6?

Answer: We thank the reviewer for highlighting this point. Figure 1 has been updated to include the number of individuals with available data at each time point, and an error has been corrected. In the control group, four participants were lost to follow-up, rather than three as previously stated; this is now consistently reported and aligns with Supplementary Table 10.

All analyses were conducted using all available data at each time point. The number of participants contributing sputum culture and radiological data at months 2/3 and 6 is lower than the number included in the final treatment outcome analysis because these intermediate assessments required on-schedule sample or imaging availability,

which was not achieved for all participants who remained in follow-up. By contrast, final treatment outcomes at month 6 could be ascertained for all 14 participants per group based on clinical records, even when intermediate microbiological or radiological measurements were missing.

We have clarified this in Figure 1 by explicitly reporting the number of participants assessed at each time point.

3. Major comment

In Figure 3 please add the p value obtained in the statistical analysis.

Answer: The p value for the analysis corresponds to 0.92, the value has been included in the figure caption.

1. Minor suggestion

Line 80, correct to COX-2.

Answer: The spelling of the protein name has been corrected as suggested.

2. Minor suggestion

Line 91, suggest rewording to "ibuprofen has never previously been tested".

Answer: The sentence has been updated as suggested.

3. Minor suggestion

Line 107, suggest rewording to "the ibuprofen groups had a higher proportion of female…"

Answer: The sentence has been updated as suggested.

4. Minor suggestion

Line 108, suggest rewording to …"with the proportions…

Answer: The sentence has been updated as suggested.

5. Minor suggestion

Line 144, suggest rewording to…"with 71% achieving …"

Answer: The sentence has been updated as suggested.

6. Minor suggestion

Line 217, suggest rewording to "..initially presenting…

Answer: The sentence has been updated as suggested.

7. Minor suggestion

Line 240, suggest rewording to "progression in TB" (delete "also").

Answer: The sentence has been updated as suggested.

8. Minor suggestion

Line 251, IL-1B

Answer: No correction done as the cytokine is listed as the Reviewer suggests.

9. Minor suggestion

Line 314, does this mean, "the current trial"?

Answer: The sentence has been corrected to clarify the description corresponds to the current trial.

10. Minor suggestion

Line 565, the title of this figure could be changed to "Immune responses changes over time".

Answer: Title has been changed as suggested.

Answers to Reviewer #3:

Comment 1:

In the Study Outcomes (Methods): thanks for the modifications. This is still difficult to follow. The definition of the primary endpoint is not clear to me. I understand (more or less) the three components of the primary outome, but how do you incorporate them to define a final outcome? I would not be able to assign this endpoint, based on this description.

Answer to reviewer:

We thank the reviewer for this comment. We agree that the previous wording did not make sufficiently clear how the primary outcome could be interpreted, and we have revised the section accordingly (lines 383–437 of the tracked-changes version). As now stated explicitly in the revised Methods, although the primary efficacy outcome was defined in the protocol as the proportion of participants showing clinical and/or microbiological benefit, it was evaluated using three prespecified measurement sets (microbiological, radiological, and final treatment outcomes), without a prespecified rule for integrating them into a single patient-level endpoint. No such rule was applied post hoc. Accordingly, no composite or final assignable endpoint was constructed, and each measurement set is analysed and reported independently.

Comment 2:

For the secondary endpoint of tolerance: do you mean that experiencing any adverse event would define a bad tolerance? Which are the "protocol-defined" AEs? In the Study Outcomes (Methods): please clarify the sentence "Adverse events were not graded using CTCAE". Do you mean that they were not graded at all?

Answer to reviewer:

Regarding the secondary endpoint of tolerance, we confirm that this was not defined by the occurrence of any adverse event. As clarified in the revised Methods, tolerance was defined based on the occurrence of prespecified protocol-defined safety events. These include worsening or exacerbation of TB, microbiological reversion, radiological worsening, clinically significant abnormalities in vital signs, clinically relevant laboratory abnormalities, or other clinically meaningful deterioration in health status. Participants experiencing any of these events were classified as not tolerating the regimen.

Regarding the grading of adverse events, they were recorded at routine study visits and reviewed but were not formally graded for severity using a standardised scale. The sentence added in the previous revision was intended to indicate the absence of Common Terminology Criteria for Adverse Events grading, but was ambiguous. We have therefore revised the text, specifically:

In the Methods section (lines 408–409 of the tracked-changes manuscript): 'Events were recorded at routine study visits, reviewed by the trial clinical team, but were not formally graded for severity'

In the Discussion (line 241 of the tracked-changes manuscript): 'The frequency of protocol-defined safety-related events was similar between study arms, and no event required intervention. Laboratory abnormalities were observed during follow-up in both arms, including during the first 2 months when ibuprofen was administered; however, adverse events were not formally graded for severity, and causality was not formally adjudicated, limiting attribution to ibuprofen versus background regimens or intercurrent illness.'

To ensure transparency and clarity, we have also added this information to the legend of Table 2 (formerly Supplementary Table 12, now moved to the main manuscript following the reviewer's comment 3)

Comment 3:

I the Results, for Safety: you refer to Figure 2, which is not presenting safety. Please include a Table in the main manuscript with the safety results, This is a key information for the readership

Answer to reviewer:

We thank the reviewer for this observation, which reflects a typographical oversight that we had not previously identified (the safety data were already fully reported in Supplementary Table 12). Following the reviewer's suggestion, and given the clinical relevance of these data, we have moved Supplementary Table 12 to the main manuscript as Table 2, with an updated legend as described above.

The in-text reference to Figure 2 for safety outcomes in the Results section has been replaced with a reference to Table 2. The supplementary table of contents has been updated accordingly.

Answers to Reviewer #4:

Comment 1:

In the revised ms the revision of the final section of the Discussion states "Finally, stronger evidence and consensus on patient selection will be essential before such strategies can be incorporated into clinical recommendations implies that Ibuprofen will subsequently be shown to have clinical benefit". So far no evidence for clinical benefit has been shown, and this sentence should be rewritten, to indicate that if it is subsequently shown to induce clinical benefit, then it could be a useful adjunctive therapy.

Answer to reviewer:

We thank the reviewer for this observation. We agree that the original wording of the final sentence of the Discussion could be interpreted as implying a subsequent demonstration of clinical benefit. This was not our intention, as no measurable clinical benefit was observed in this study. Following the reviewer's suggestion, we have revised the sentence to: "Finally, any consideration of incorporating such strategies into clinical recommendations will require robust evidence of clinical benefit and clearer definition of appropriate patient populations."
